# MicroRNAs as biomarkers for prostate cancer prognosis: a systematic review and a systematic reanalysis of public data

**DOI:** 10.1038/s41416-021-01677-3

**Published:** 2022-01-12

**Authors:** Sharmila Rana, Gabriel N. Valbuena, Ed Curry, Charlotte L. Bevan, Hector C. Keun

**Affiliations:** 1 Division of Cancer, Department of Surgery and Cancer, Imperial College London, London, W12 0NN UK; 2 Wellcome Centre for Human Genetics, University of Oxford, Oxford, OX3 7BN UK; 3 GSK, Stevenage, SG1 2NY UK

**Keywords:** Prostate cancer, Prognostic markers

## Abstract

**Background:**

Reliable prognostic biomarkers to distinguish indolent from aggressive prostate cancer (PCa) are lacking. Many studies investigated microRNAs (miRs) as PCa prognostic biomarkers, often reporting inconsistent findings. We present a systematic review of these; also systematic reanalysis of public miR-profile datasets to identify tissue-derived miRs prognostic of biochemical recurrence (BCR) in patients undergoing radical prostatectomy.

**Methods:**

Independent PubMed searches were performed for relevant articles from January 2007 to December 2019. For the review, 128 studies were included. Pooled-hazard-ratios (HRs) for miRs in multiple studies were calculated using a random-effects model (REM). For the reanalysis, five studies were included and Cox proportional-hazard models, testing miR association with BCR, performed for miRs profiled in all.

**Results:**

Systematic review identified 120 miRs as prognostic. Five (let-7b-5p, miR-145-5p, miR152-3p, miR-195-5p, miR-224-5p) were consistently associated with progression in multiple cohorts/studies. In the reanalysis, ten (let-7a-5p, miR-148a-3p, miR-203a-3p, miR-26b-5p, miR30a-3p, miR-30c-5p, miR-30e-3p, miR-374a-5p, miR-425-3p, miR-582-5p) were significantly prognostic of BCR. Of these, miR-148a-3p (HR = 0.80/95% CI = 0.68-0.94) and miR-582-5p (HR = 0.73/95% CI = 0.61-0.87) were also reported in prior publication(s) in the review.

**Conclusions:**

Fifteen miRs were consistently associated with disease progression in multiple publications or datasets. Further research into their biological roles is warranted to support investigations into their performance as prognostic PCa biomarkers.

## Background

Prostate cancer (PCa) is the most common cancer and second most lethal cancer in men in the UK over 40,000 cases were diagnosed and around 11,700 deaths occurred every year between 2015 and 2017 [1]. It is a heterogeneous disease and can manifest as either a low-risk, indolent tumour localised to the prostate or a high-risk, aggressive tumour that eventually metastasises and proves lethal if untreated. As many as 42–66% of patients present with the indolent form of PCa [2, 3]. Over the last three decades, PCa incidence has rapidly increased while the mortality rate has remained relatively stable [1]. This rise in incidence is attributed to the widespread use of the prostate specific antigen (PSA) test in diagnosing PCa. However, this test is not specific and results in a high proportion of false positives as well as detection of indolent disease [2, 4]. This has led to over-treatment of patients without any benefit in overall survival, evidenced by the steady mortality rate, and has led to an increase in PCa disease burden. Additionally, 15–45% of patients treated with radical prostatectomy (RP), one of the first-line curative treatments for localised PCa, experience biochemical recurrence (BCR) within 5-years [5–8]. Although BCR does not always equate to clinical recurrence, it is considered an initial event signifying disease progression and has shown to be associated with increased risk of PCa metastasis and cancer-specific mortality [7–11]. These problems highlight the importance of reliable and accurate identification of aggressive disease as distinct from indolent disease in order to limit over-treatment and provide appropriate treatment strategies for the management of PCa.

Current prognostic markers used for disease management decisions are based on risk stratification systems which incorporate clinicopathological variables Gleason score, pathological tumour stage and serum PSA at diagnosis [12, 13]. Although these variables are good indicators of disease severity and correlate with patient survival, their measurements are subject to sampling and random errors as the biopsies may miss tumours, resulting in a high proportion of misdiagnoses. More than 30% transrectal ultrasound biopsies are false negatives and higher than 45% of cancer patients have their Gleason scores underestimated [14, 15]. In addition to the risk stratification systems, there are various promising multi-omic biomarker panels currently being explored for prognostication such as the OncotypeDX Genomic Prostate Score, Decipher and Prolaris tests [16–18]. These panel-based tests have been validated in large external cohorts and often reach an AUC higher than 0.7 [17–21]. However, these tests are not widely available, for instance in the UK they are only commercially available in some private clinics, severely limiting accessibility both financially and geographically.

MiRs have been investigated for their potential to serve as alternative molecular markers for PCa. MiRs are small non-coding RNAs that negatively regulate gene expression at the post-transcriptional level. They do so by binding to complementary sequences in the 3′UTR of target mRNAs via a preserved ‘seed sequence’ region, which then represses translation of the target mRNAs [22]. Due to their regulatory role, these molecules have been implicated in various developmental, cellular and physiological processes and their dysregulation has been associated with various diseases [22, 23]. Differential miR expression profiles between tumour and normal tissues have been observed in various cancers, including PCa [24–27]. Other advantages as biomarkers inlcude: miRs are abundantly and stably expressed in-vivo, detected in biofluids such as blood, urine and saliva, and are highly stable in storage [26–31].

The first extensive miR expression profiling in PCa cell lines, xenograft samples and clinical tumour samples was published in 2007 by Porkka and colleagues [32]. Since then, numerous studies have characterised miR expression profiles in PCa tissues and bio-fluids at various stages of the disease and examined their prognostic potential [27, 30, 33–42]. A major caveat to these studies is that they often report inconsistent results, possibly due to heterogeneity between studies, including differences in study designs, methodologies, and clinically diverse populations. Thus, there is no general consensus to date on the miRs that truly associate with disease progression and have the potential to be utilised as prognostic biomarkers for PCa. Attempts at meta-analyses to combine results from multiple studies and appraise the current miR biomarker landscape are limited to only a handful of publicly available datasets [43]. A systematic review, which does not require the disclosure of sensitive clinical datasets, may be more useful in examining the prognostic miR biomarker landscape in PCa and identifying consistent patterns across the studies. As yet, no such systematic review covering the topic of prognostic miR biomarkers in PCa has been published.

In this study, we aimed to review the relevant existing publications in the scientific literature to date and identify consistently reported miRs with potential as prognostic biomarkers in PCa. First, a systematic review was performed of studies that investigated the prognostic potential of individual miRs or miR panels in PCa. A comprehensive approach was taken in which any publications evaluating prognostic miRs were included, irrespective of methodological or clinical diversity. The review revealed a considerable number of publications that investigated the association of tumour tissue-derived miRs with BCR in patients who have undergone RP. The only meta-analysis of primary data addressing prognostic miRs in PCa was performed in 2017 [43]. To account for new public datasets after this, an updated reanalysis was performed on studies with publicly accessible global miR expression datasets. Based on the results of the systematic review, we redefined the aim to focus on identifying miRs that are prognostic of BCR in patients that have undergone RP. Here, only tissue-specific miRs were considered as the majority of publications (∼88%) in the systematic review addressed tissue-derived miRs.

## Methods

The systematic review and data reanalysis were conducted in accordance with the PRISMA guidelines [44].

### Methodology for systematic review

#### Search strategy

A methodological search of electronic database PubMed was performed on 24th of January, 2020 for relevant studies published between January 2007 and December 2019. The keywords searched were ‘prostate cancer microRNAs prognosis relapse outcome’. This search included both free words and MeSH terms, ensuring all publications with the keywords and related terms in their title or body were included in the search result. The MeSH terms associated with the keywords were: (‘micrornas’[MeSH Terms] OR ‘micrornas’[All Fields] OR ‘mirnas’[All Fields] OR ‘miRs’[All fields] OR ‘microrna’[All Fields] OR ‘mirna’[All Fields] OR ‘miR’[All fields]) AND (‘prostatic neoplasms’[MeSH Terms] OR (‘prostatic’[All Fields] AND ‘neoplasms’[All Fields]) OR ‘prostatic neoplasms’[All Fields] OR (‘prostate’[All Fields] AND ‘cancer’[All Fields]) OR ‘prostate cancer’[All Fields]) AND (‘prognosis’[MeSH Terms] OR ‘prognosis’[All Fields] OR ‘recurrence’[MeSH Terms] OR ‘recurrence’[All Fields] OR ‘relapse’[All Fields] OR ‘mortality’[Subheading] OR ‘mortality’[All Fields] OR ‘survival’[All Fields] OR ‘survival’[MeSH Terms] OR ‘outcome’[All Fields]).

#### Study eligibility

Studies were selected according to the following criteria:

(i) the study measured expression of miRs in tissues or circulation of PCa patients (not xenograft or other animal models);
(ii) the study performed a survival analysis to examine the association of miRs with outcome: Cox PH regression model or Kaplan–Meier (KM) analysis, and appropriate test statistics such as hazard ratio (HR), 95% confident intervals (CI) and log-rank *p*-values were reported in the main text or supplementary section.

Studies were excluded if:

(i) the study tested the prognostic role of miR host genes or target genes instead of the miR itself;
(ii) the study tested the prognostic role of miR in combination with non-miR markers such as clinical factors, genes or proteins;
(iii) the study was in a different language with no English translation available;
(iv) the study was a meta-analysis, review, comment, letter or duplicate publication.

#### Data extraction

The following data were extracted from each eligible study: PMID, surname of first author, year published, title, miR(s) investigated, sample size, sample type, detection method, outcome endpoint, endpoint definition, test type (Cox PH/KM), effect estimates (HR, 95% CI or log-rank *p*-value), Cox PH test type (univariate/multivariate), adjusted variables (if multivariate Cox PH). If the study performed both Cox PH model and KM analysis, only the results for Cox PH model was extracted.

#### Statistical analysis

For the miRs that had multiple entries for the same endpoint and had their Cox PH test statistics reported, a meta-analysis was performed in order to calculate the summary effect size (pooled HR). For miR entries originating from the same study, a fixed-effects model (FEM) approach was employed. For miR entries from different studies, we hypothesised that due to biological and technological diversity, the true effect size varied across studies. Thus, a random-effects model (REM) approach was employed for these miRs. Low miR expression was set as the reference group, so for entries with high miR expression as the reference group, reciprocal of HR and 95% CI were calculated. Between study heterogeneity was assessed using Cochran’s Q-test and Higgins I^2^ statistic. Significance for the Q-test was defined as *p* *<* 0.05. Due to the very small number of studies considered in the meta-analysis, publication bias was not assessed. The meta-analysis and heterogeneity tests were performed in statistical software R using package metafor (version 2.4.0) [45].

#### MiR annotation

As the search spanned more than a decade, the miR annotation was outdated in many of the studies. For such cases, the article was screened in order to obtain strand information for the miR of interest. If strand information was not stated in the article, the miR was assumed to be the dominant strand. The miR name was then cross-referenced with its entry in the miRBase database, which contains an archive of miR annotations and sequences for all species and updated to the most recent version (version 22) [46]. MiR names were left unchanged if the dominant/ passenger strand in miRBase was not specified.

### Methodology for systematic reanalysis of public miR datasets

#### Search strategy

A methodological search of electronic database PubMed was performed on 23rd of April, 2020 in order to identify relevant studies published between January 2007 and December 2019. The keywords searched were ‘prostate cancer relapse microRNA expression’. The MeSH terms associated with the keywords were: (‘prostatic neoplasms’[MeSH Terms] OR (‘prostatic’[All Fields] AND ‘neoplasms’[All Fields]) OR ‘prostatic neoplasms’[All Fields] OR (‘prostate’[All Fields] AND ‘cancer’[All Fields]) OR ‘prostate cancer’[All Fields]) AND (‘recurrence’[MeSH Terms] OR ‘recurrence’[All Fields] OR ‘relapse’[All Fields]) AND (‘micrornas’[MeSH Terms] OR ‘micrornas’[All Fields] OR ‘mirna’[All Fields]) AND (‘gene expression’[MeSH Terms] OR (‘gene’[All Fields] AND ‘expression’[All Fields]) OR ‘gene expression’[All Fields] OR ‘expression’[All Fields]).

#### Study eligibility

Studies were selected according to the following criteria:

(i) the study measured miR expression in tissues of PCa patients who underwent RP and no other curative therapy (no studies with miRs profiled in circulation);
(ii) the study generated global miR expression profiling dataset which was available in public data
  repositories;
(iii) the study contained follow-up data, i.e. BCR status of patients and time to BCR.

Studies were excluded if:

(i) the study was in a different language with no English translation available;
(ii) the study was a meta-analysis, review, comment, letter, or duplicate publication.

For studies with publicly accessible expression datasets and insufficient follow-up information, corresponding authors were directly contacted for additional clinical information. Studies that examined miR expression profile without generating novel data were also included in order to examine if the datasets they used were suitable for our reanalysis.

#### Data extraction and normalisation

Five studies, which included six datasets, were eligible for the data reanalysis (Table 1). For the TCGA-PRAD dataset, access to raw miR-sequencing data was granted through the NIH database of Genotypes and Phenotypes, and the raw miR-sequencing data and associated clinical data were downloaded from the Genomic Data Commons data portal via the data transfer tool and Bioconductor package TCGAbiolinks (version 2.12.6) [47–49]. Raw miR expression data was normalised using the trimmed mean of M-values method using the *edgeR* package (version 3.26.8) [50]. MiRs were then filtered to include only those with normalised read counts ≥1 counts per million in at least 80% of samples, which left 328 miRs. For the rest of the datasets, normalised miR expression data and associated clinical data were obtained from the NCBI Gene Expression Omnibus database [51]. For GSE21036, clinical information was supplemented with clinical data obtained from the data repository in the MSKCC computational biology centre website [https://cbio.mskcc.org/cancergenomics/prostate/data/]. For GSE26245 and GSE26247, clinical information was supplemented with clinical data provided in the supplementary section of their corresponding paper [52]. For GSE46738 and GSE88958, the corresponding authors directly provided follow-up data (Leite K., written communication, 27 June 2018; Ozen M., written communication, 18 January 2019). The normalised datasets were standardised according to *z*-score transformation. MiR annotation in each dataset was also updated to miRBase version 22 using package miRBaseConverter (version 1.8.0) [53].

**Table 1:** Characteristics of the studies included in the data reanalysis.

Study ID	Profiling technology	Endpoint definition	Surgery	Sample type	n with follow-up	No. miRs	Ref.
GSE21036	Agilent-019118 Human miRNA Microarray 2.0 G4470B	PSA ≥ 0.2 ng/ml on two occasions	RP	Tissue	99	373	[116]
GSE26245	llumina Custom Prostate Cancer DASL Panel miRNA & 2 detectable PSA readings	two detectable PSA readings (> 0.2 ng/mL)	RP	Tissue FFPE	63	733	[52]
GSE26247	llumina Custom Prostate Cancer DASL Panel miRNA & 2 detectable PSA readings	two detectable PSA readings (> 0.2 ng/mL)	RP	Tissue FFPE	40	1145	[52]
GSE46738	Affymetrix Multispecies miRNA-1 Array & PSA	PSA > 0.2 ng/ml	RP	Tissue frozen	50	847	[117]
GSE88958	Agilent 8x15 K Human V3 microRNA Microarray	PSA ≥ 0.2 ng/ml on two occasions & RP & tissue	RP	Tissue	30	847	[41]
TCGA-PRAD	Illumina GAIIx or HiSeq 2000 miRNA Sequencing	PSA > 0.2 ng/ml at two or more occasions & RP	RP	Tissue frozen	433	328	[118]

*FFPE* fresh-frozen paraffin-embedded, *PSA* prostate-specific antigen, *RP* radical prostatectomy.

#### Statistical analyses

Serum PSA at diagnosis, Gleason score sum and clinical tumour stage were available in five of the six datasets. Only GSE88958 did not contain tumour stage information. To account for this, firstly, a univariate Cox PH analysis was performed in each of the six datasets, where the only predictor being tested for association with disease relapse was miR expression. Secondly, a multivariate Cox PH analysis was performed in each of the five datasets with all three clinicopathological features available. Here, the Cox PH model included miR expression as the main predictor with PSA, Gleason score sum and tumour stage as confounders. Cox PH regressions were performed using R package survival (version 3.1.12) [54]. Analysis of variance (ANOVA), Kruskal–Wallis (KW) and Chi-squared (*X*^2^) tests were also performed to test whether the distribution of the clinical variables differed between the datasets.

Following univariate/multivariate Cox PH analysis, a REM meta-analysis was performed to calculate the pooled HR of the miRs across the studies. The meta-analysis was performed only for miRs that were present in all the datasets. Subsequently, a total of 162 and 164 miRs were evaluated in the univariate and multivariate analyses, respectively. The significance threshold was set at *p*-value < 0.05. As very few studies were included in this meta-analysis, publication bias was not assessed.

## Results

### Prognostic miRs in localised prostate cancer: a systematic review

#### Study selection and characteristics

**Fig. 1:**
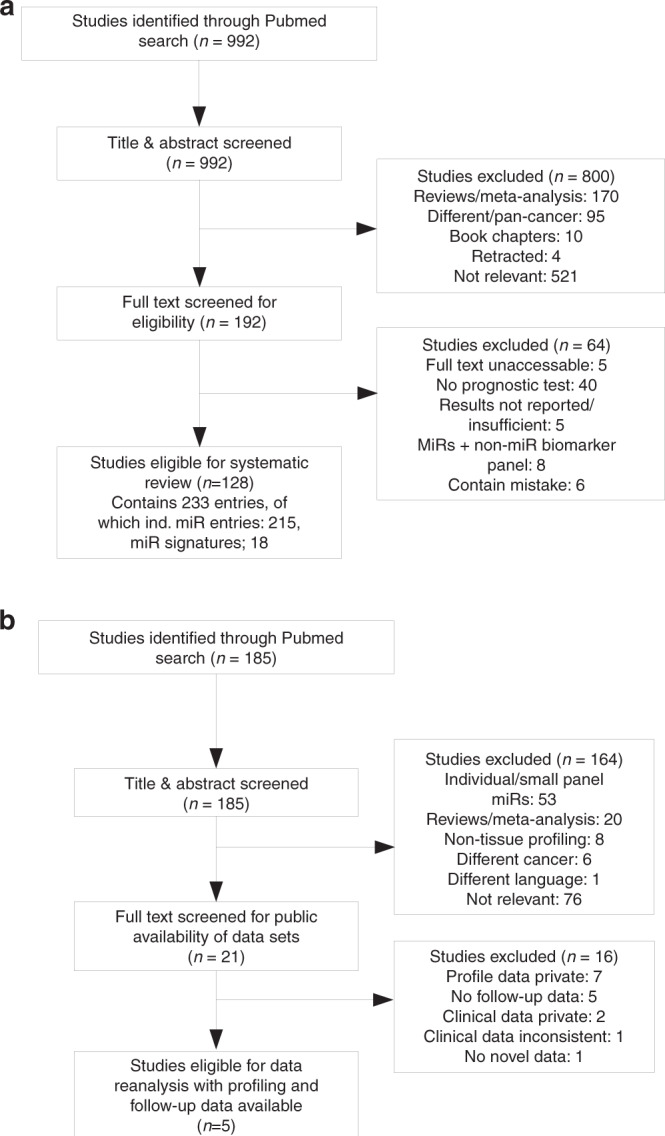
Workflow for selecting eligible studies in the review. Workflow for the systematic review (**a**) and data reanalysis (**b**). Majority of the studies in the initial searches had themes on cancer, miRs and/or molecular biomarkers but did not directly address the primary subjects of the paper (i.e. PCa recurrence and miR biomarkers), so did not qualify for the analysis and were labelled as ‘not relevant’.

A total of 992 studies were retrieved from the initial literature search. Title and abstract screening removed 800 non-relevant studies such as meta-analyses, book chapters, reviews and other irrelevant publications. Full-text screening removed a further 64 studies for reasons such as inaccessibility of full text, insufficient reporting of results, no prognostic test performed and containing mistakes such as incorrect CIs or female PCa sample population. Ultimately, 128 studies were eligible and included 215 entries for individually prognostic miRs (containing 120 unique miRs) and 18 entries for miR signatures panels (containing 8 unique miR signatures). Workflow for study selection is detailed in Fig. 1a.

The majority of miR biomarkers were detected using variations of the PCR technique (qPCR, RT-PCR, qRT-PCR). Less frequent detection methods were microarrays, (small-)RNAseq, NanoString, in situ hybridization, mass spectrometry and BeadChip based technologies. The review included biomarkers extracted from a variety of sources such as tissues (*n* = 204), blood (whole blood, peripheral blood, serum, plasma; *n* = 23), exosomes (*n* = 2), urine (*n* = 2) and cells (epithelial and stromal, fibroblast) (*n* = 2). The different clinical trial endpoints used by the studies in the review are listed in Table 2. The most common endpoint used as a surrogate for progression was biochemical recurrence-free survival (BPFS; 44.64%), followed by overall survival (OS; 20.17%). The remaining endpoints each accounted for less than 10% of the studies in the review. The study characteristics, statistical results, endpoint definitions and additional variables included in the survival analysis (if a multivariate Cox PH was performed) are summarised in Supplementary Tables S1 and S2 for individually prognostic miRs and in Supplementary Tables S3 and S4 for prognostic miR signatures.

**Table 2:** Progression endpoints considered in the systematic review.

Endpoint	Abbreviation	Number of entries (%)
Bone metastasis-free survival	bone MFS	8 (3.43)
Biochemical progression/ recurrence-free survival	BPFS	104 (44.64)
Clinical failure-free survival	CFFS	7 (3.00)
Castration resistant prostate cancer-free survival	CRPC FS	6 (2.58)
Cancer-specific survival	CSS	6 (2.58)
Disease-free survival	DFS	11 (4.72)
Disease-specific survival	DSS	2 (0.86)
Metastasis-free survival	MFS	6 (2.58)
Overall suvival	OS	47 (20.17)
Progression-free survival	PFS	5 (2.17)
Percentage survival	PS	13 (5.58)
Recurrence/relapse-free survival	RFS	18 (7.73)

Twelve different endpoints were considered. After verifying endpoint definitions in respective studies, endpoints with redundant meanings were categorised into the same group. If the studies did not provide definitions or the definitions were different between studies, endpoint with redundant meanings were not categorised together.

#### Individually prognostic miR biomarkers in PCa

We accumulated 215 entries reporting 120 unique prognostic miRs in PCa (Supplementary Table S1). 44 unique miRs had multiple entries in the review. These miRs were either evaluated against different endpoints/ cohorts in the same study or were evaluated more than once in separate studies. Of these, 36 miRs had Cox PH output available. A REM meta-analysis was performed for the miRs evaluated against the same endpoint to determine their overall association (Supplementary Fig. S1). Seven miRs - let-7b-5p, miR-128a-3p, miR-188-5p, miR-224-5p, miR-23a-3p, miR-23b-3p and miR-34b/c consistently and significantly associated with progression. High expression of miR-34b/c and miR-23a-3p associated with poor disease outcome, while for the remaining 5 miRs high expression associated with better disease outcome. The Q-test for heterogeneity was not significant for these miRs (where meta-analysis was performed). Similarly, the I^2^ statistic ranged between 0.00 and 4.30%, suggesting absence of statistical heterogeneity. A forest plot was also generated for the remaining miRs with single entries (Supplementary Fig. S2).

Considering the effect sizes of both Cox PH and KM outputs for the 120 unique miRs, 57 miRs negatively associated with progression, 43 miRs positively associated with progression and 20 miRs had an inconsistent direction of association. Only four miRs, let-7b-5p, miR-152-3p, miR-195-5p and miR-224-5p, significantly and consistently associated with progression in multiple patient cohorts in the same study or in at least two independent studies. Additionally, miR-145-5p had an insignificant but consistent trend in association with progression in five independent studies. Low expression of all five miRs consistently associated with shorter time to disease progression (Table 3, Supplementary Fig. S1). These miRs are the strongest prognostic biomarker candidates for PCa based on current literature.

**Table 3:** The miRs with consistent direction of association to disease progression, irrespective of different endpoints, that have been validated in multiple cohorts or independent studies.

Prognostic miR	Prognostic test			Reference group	Association after reference standardisation	Sample size	Sample type	PMID	Ref
	Test: endpoint	HR (95 % CI)	*p*						
let-7b-5p	Multivariate: BPFS	0.44 (0.19–01.02)	0.05	Low	Negative	98 (cohort A)	Tissue	23798998	[102]
	Multivariate: BPFS	0.30 (0.15–0.61)	<0.010	Low	Negative	92 (cohort B)	Tissue		
	Multivariate: CFFS	0.23 (0.08–0.70)	<0.010	Low	Negative	92 (cohort B)	Tissue		
	Multivariate: CFFS	0.46 (0.15–1.41)	0.17	Low	Negative	98 (cohort A)	Tissue		
miR-145-5p	Multivariate: BPFS	4.47 (1.27–15.74)	0.02	High	Negative	36 (low + intermediate risk)	Tissue	23703249	[103]
	Multivariate: BPFS	4.43 (1.11–17.61)	0.035	High	Negative	29 (intermediate risk)	Tissue		
	Multivariate: PFS	0.40 (0.17–0.94)	0.036	Low	Negative	106	Tissue	20332242	[104]
	Univariate/KM: OS	3.00 (1.60–7.00)	<0.010	High	Negative	49	Tissue	25969144	[105]
	Univariate: BPFS	0.74 (0.23–2.34)	0.609	Low	Negative	76	Tissue	19676045	[34]
	Univariate: BPFS	0.68 (0.222.14)	0.51	Low	Negative	73	Tissue	22864280	[106]
	Multivariate: DFS	1.26 (0.49–3.27)	0.629	High	Negative	73	Tissue	23703249	[103]
miR-152-3p	KM: BPFS	–	<0.001	Low	Negative	n/s (MSKCC)	Tissue	25004396	[107]
	Multivariate: DFS	0.23 (0.07–0.72)	0.012	Low	Negative	494 (TCGA)	Tissue	29599847	[108]
miR-195-5p	Multivariate: BPFS	5.96 (1.18–30.02)	0.031	High	Negative	140	Tissue	26338045	[109]
	Multivariate: OS	4.46 (1.35–14.72)	0.014	High	Negative	140	Tissue		
	Multivariate: BPFS	0.61 (0.41–0.93)	0.022	Low	Negative	107 (MSKCC)	Tissue	26080838	[110]
	KM: BPFS	–	0.009	Low	Negative	131 (MSKCC)	Tissue	30032144	[111]
	KM: RFS	–	0.049	Low	Negative	98 (MSKCC)	Tissue	26650737	[112]
	KM: DFS	–	<0.010	Low	Negative	n/s (MSKCC)	Tissue	27175617	[113]
miR-224-5p	Multivariate: BPFS	0.25 (0.08–0.74)	0.01	Low	Negative	114	Tissue	24382668	[114]
	Multivariate: BPFS	0.64 (0.14–2.39)	0.525	Low	Negative	58	Tissue	23136246	[115]

KM, univariate and multivariate tests stand for Kaplan–Meier analysis, and univariate and multivariate Cox PH regressions, respectively. For the test entry ‘univariate/ KM’, both univariate Cox PH and KM analysis were performed, but the *p*-value for the univariate Cox PH regression was not reported. Thus, the HR and 95% CI corresponds to outputs of the univariate Cox PH regression and the *p*-value corresponds to the KM log-rank test. For studies that performed multivariate analysis, the different variables adjusted for are reported in Table S2. The values in the ‘Prognostic test’ and ‘Reference group’ columns refer to the statistics and the reference group used for comparison as reported in respective papers. In contrast, the ‘Association after reference standardisation’ column refers to the association of the miRs to progression after standardising the comparisons to ‘low’ miR expression as the reference group. n/s represents not-specified.

#### Prognostic miR signatures as biomarker panels in PCa

Eight miR signatures, comprised of 36 unique miRs, were reported as prognostic in eight independent studies (Supplementary Table S3). The majority of these studies performed independent clinical validations and/or have large sample sizes (& 100), making their findings robust. Interestingly, only Feng et al. (2017) investigated a panel of miRs that were biologically related, in this case the miRs in the signature panel were all part of the miR-17/92 cluster [55]. The remaining studies grouped miRs into signature panels if they were significantly differentially expressed between recurrent and nonrecurrent cases or individually had significant predictive power to distinguish between recurrent and non-recurrent cases.

Within the eight signatures, only miRs let-7a-5p and miR-223 were present in multiple miR signatures. In Mihelich et al., both were grouped into a panel with five other miRs and their expression levels were significantly downregulated in recurrent patients compared to non-recurrent patients [56]. In Nam et al. miR-223, and in Fredsoe et al. let-7a-5p, were grouped into signature panels for their predictive power to significantly distinguish between recurrent and non-recurrent PCa cases [57, 58]. Interestingly, although prognostic as part of miR signatures, neither let-7a-5p nor miR-223 have been reported as individually prognostic predictors. However, 16 out of the 36 unique miRs in the signature panels (miR-10b-5p, -130b-3p, -139-5p, -145-5p, -17-5p, -19a-3p, -200b-3p, -20a-5p, -221-3p, -23a-3p, -301a3p, -326, -374b-5p, -375, -652-3p and -96-5p) were reported as individually prognostic in multiple studies (Supplementary Table S1). For 11 out of these 16 (miR-10b-5p, -130b-3p, -145-5p, -17-5p, -19a-3p, -23a-3p, -301a-3p, -326, -374b-5p, -652-3p and -96-5p), their individual association with progression in corresponding studies was consistent with the direction of expression in signature panel studies.

### Identification of miR biomarkers for prostate cancer recurrence following radical prostatectomy: a systematic reanalysis of publicly available miR profile data

#### Study selection and sample characteristics of eligible datasets

A total of 185 studies were retrieved from the initial literature search. After title and abstract screening, 164 ineligible articles such as meta-analyses, reviews and studies based on non-tissue datasets or non-PCa studies were removed. Full-text screening removed a further 16 studies as their datasets were not publicly available (*n* = 7), did not have follow-up information (*n* = 5), could not share clinical information due to patient confidentiality (*n* = 2), contained inconsistent clinical information (*n* = 1) or were categorised as duplicate due to using public datasets already included in this reanalysis (*n* = 1). Ultimately, five studies, containing six datasets, were eligible for the reanalysis. The workflow for the selection of studies is detailed in Fig. 1b and study characteristics are reported in Table 1.

MiRs were profiled from tissue samples collected from men who underwent RP in all datasets. The endpoint for the datasets was BCR, which was defined by the majority of the datasets as a rise in serum PSA levels ≥0.2ng/ml on two or more occasions, consistent with the European Association of Urology guidelines [59]. Only GSE36738 did not specify the number of rising PSA measurements required to classify a BCR event. The majority of the datasets contained accompanying clinical variables: age at diagnosis, PSA at diagnosis, Gleason score and tumour stage. Only GSE88958 did not contain tumour stage information. The sample characteristics for these studies are provided in Supplementary Table S5.

#### Association of clinicopathological features with disease relapse

The associations of clinicopathological features (age, serum PSA at diagnosis, Gleason score sum and tumour stage) with disease relapse were tested in each dataset, and a REM meta-analysis model was employed to summarise the overall effect across the datasets (Supplementary Fig. S3). Although non-significant, higher age and PSA levels at diagnosis associated with a higher risk of BCR (Supplementary Fig. S3a, S3b). Higher Gleason score sum (≥8) and higher tumour stages (T3+T4) had a significant and stronger association with BCR (pooled HR *>*3; Supplementary Fig. S3c, S3d). Gleason score sum, tumour stage and PSA at diagnosis are the standard prognostic features as per the National Institute for Healthcare and Excellence and European Association of Urology guidelines [12, 13]. Thus, the multivariate models testing the association of miR expression with BCR were adjusted for these three confounding variables.

#### MiRs that consistently associate with disease relapse: a univariate analysis

Univariate Cox PH regression followed by a REM meta-analysis was performed for 162 miRs that were common in all six datasets. Pooled HR estimates for 18 miRs were significantly associated with BCR (Table 4, Supplementary Fig. S4). Of these, 17 miRs (let-7a-5p, miR-125b-5p, -133a-3p, -135a-5p, -148a-3p, -155-5p, -203a-3p, -204-5p, -218-5p, -222-3p, -26b-5p, -30a-3p, -30c-5p, -30e-3p, -374a-5p, -455-5p and miR-582-5p) had negative association, while only miR-425-3p had positive association with BCR. The Q-test for heterogeneity was not significant for any of the miRs and I^2^ statistic ranged from 0-40%, suggesting moderate levels of heterogeneity between the datasets.

**Table 4:** MiRs significantly associated with biochemical recurrence in both univariate and multivariate meta-analyses.

miRs	Univariate Cox PH		Multivariate Cox PH		Systematic review				
	Pooled HR	CI (95%)	Pooled HR	CI (95%)	Test: endpoint	HR	CI (95%)	Sample size	Reference
let-7a-5p	0.81	0.69–0.96	0.82	0.68–0.98					
miR-1-3p	–	–	0.81	0.68–0.97					
miR-125b-5p	0.86	0.74–1.00	–	–					
miR-130b-3p	–	–	1.37	1.10–1.71					
miR-133a-3p	0.81	0.71–0.94	–	–					
miR-135a-5p	0.82	0.70–0.96	–	–					
miR-148a-3p	0.83	0.71–0.97	0.8	0.68–0.94	Multivariate: BPFS	0.6	0.44–0.81	207	[60]
miR-155-5p	0.82	0.67–1.00	–	–					
miR-181b-5p	–	–	1.21	1.01–1.44					
miR-20a-5p	–	–	0.85	0.73–0.99					
miR-203a-3p	0.79	0.67–0.93	0.8	0.68–0.94	KM: PS	2.52	1.11–4.88	44	[63]
miR-204-5p	0.83	0.71–0.97	–	–					
miR-218-5p	0.85	0.72–1.00	–	–					
miR-221-3p	–	–	0.86	0.74–1.00					
miR-222-3p	0.76	0.64–0.89	–	–					
miR-26b-5p	0.86	0.74–1.00	0.82	0.68–0.99					
miR-30a-3p	0.73	0.59–0.91	0.81	0.66–1.00					
miR-30c-5p	0.82	0.69–0.97	0.81	0.67–0.98	Multivariate: BPFS	0.34	0.17–0.68	103	[62]
					Multivariate: BPFS	0.49	0.28–0.85	207	[60]
					Univariate: PS	2.38	1.09–5.22	44	[63]
miR-30e-3p	0.71	0.60–0.86	0.78	0.66–0.92					
miR-30e-5p	–	–	0.81	0.69–0.96					
miR-374a-5p	0.8	0.68–0.94	0.82	0.69–0.98					
miR-425-3p	1.25	1.05–1.48	1.27	1.05–1.53					
miR-455-5p	0.78	0.64–0.96	–	–					
miR-582-5p	0.68	0.57–0.80	0.73	0.61–0.87	KM: bone MFS	0.21	0.10–0.45	94	[61]

A set of 18 and 16 miRs were significant in the univariate and multivariate analyses, respectively. Ten miRs were significant in both analyses. Four miRs (miR-148a-3p, miR-203a-3p, miR-30c-5p and miR-582-5p) out of these ten have been identified as prognostic in independent publications, although the direction of association with progression is not consistent for miR-203a-3p and miR-30c-5p between my findings and the independent publications. KM, univariate and multivariate tests refer to Kaplan–Meier analysis, univariate Cox PH regression and multivariate Cox PH regression respectively. In the multivariate Cox PH, the adjusted variables were Gleason score, tumour stage, and PSA. A total of five and six datasets were included in the univariate and multivariate meta-analyses, respectively. *KM* Kaplan–Meier. For the full form of the abbreviated endpoints, refer to Table 2.

#### MiRs that consistently associate with disease relapse: a multivariate analysis

A total of 164 miRs were common between the five datasets considered for the multivariate analysis.

The analysis revealed only 16 miRs significantly associated with BCR (Table 4, Supplementary Fig. S5). 13 miRs (let-7a-5p, miR-1-3p, -148a-3p, -203a-3p, -20a-5p, -221-3p, -26b-5p, -30a-3p, -30c-5p, -30e-3p, -30e-5p, -374a-5p and -582-5p) had negative association and three miRs (miR-130b-3p, -181b-5p and -425-3p) had positive association with disease relapse. The Q-tests for heterogeneity for these miRs were non-significant and the I^2^ value ranged from 0 to 30%. These values represent moderate to no heterogeneity between the datasets. Overall, ten miRs (let-7a-5p, miR-148a-3p, -203a-3p, -26b5p, -30a-3p, -30c-5p, -30e-3p, -374a-5p, -425-3p and -582-5p) were significantly prognostic in both univariate and multivariate meta-analyses (Table 4). The consistent trend in association with relapse of these 10 miRs, despite clinical and methodological differences between the datasets and even after being adjusted for confounding clinicopathological features, demonstrates replicability and robustness, and supports these miRs as ideal candidates for further investigation as prognostic PCa biomarkers.

### MiRs with consistent association with prostate cancer progression: agreement between systematic review and data reanalysis

In the systematic review, five miRs, let-7b-5p, miR-145-5p, miR-152-3p, miR-195-5p and miR-224-5p, were identified as consistently individually prognostic, of which the latter four miRs were evaluated in the multivariate meta-analysis. However, the association of these four miRs with BCR were non-significant and inconsistent in the data reanalysis (Supplementary Fig. S6).

**Fig. 2:**
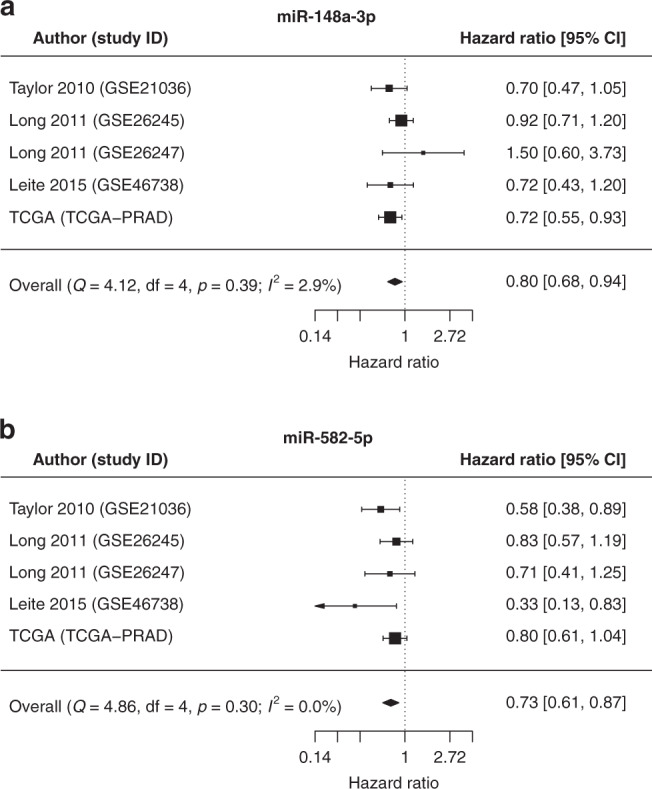
Association of miR-148a-3p and miR-582-5p with biochemical relapse (BCR). MiR-148a-3p (**a**) and miR-582-5p (**b**) expression levels show significant association to BCR in the multivariate meta-analysis.

In the reanalysis overall, ten miRs, let-7a-5p, miR-148a-3p, miR-203a-3p, miR-26b-5p, miR-30a-3p, miR-30c-5p, miR-30e-3p, miR-374a-5p, miR-425-3p and miR-582-5p, were validated as significantly prognostic of BCR post-RP. Among these, only four miRs (miR-148a-3p, miR-582-5p, miR-30c-5p and miR-203a-3p) were identified as individually prognostic in the systematic review (Table 4). The direction of association of miR-148a-3p and miR-582-5p with progression endpoints BPFS and bone metastasis-free survival, respectively, in the review were consistent with the direction of association of the miRs with BCR in the reanalysis [60, 61]. MiR-30c-5p was reported as prognostic in three independent studies; Ling et al. and Zhao et al. reported negative association of miR-30c-5p expression with BPFS, which were consistent with the results from the meta-analysis [60, 62]. However, the findings of Huang et al. were inconsistent as they reported positive association of miR-30c expression with PCa patient survival [63]. For miR-203a-3p, its direction of association with survival also conflicted with the findings of the reanalysis [63]. The inconsistencies for miR-30c-5p and miR-203a-3p could potentially be due to differences in endpoints or statistical approaches, such as inclusion of different confounder variables in the multivariate models. Although there were no overlaps between miRs identified as of interest in the systematic review and reanalysis, two miRs: miR-148a-3p and miR-582-5p (Fig. 2, Table 4), were identified as consistently predictive of BCR in the reanalysis and had at least one publication in the systematic review verifying their association [60, 61]. Therefore, these two miRs are ideal candidates to follow-up as individual prognostic markers for PCa.

## Discussion

In this work, we addressed the problem of inconsistent and conflicting reports of prognostic miRs in PCa in the literature by undertaking a systematic review that summarised and identified consistently reported prognostic miR biomarkers in localised PCa. We next performed a systematic reanalysis of six publicly available miR profile datasets, which identified tumour tissue-derived miRs consistently associated with BCR in post RP samples. Two miRs miR-148a-3p and miR-582-5p were validated as independently prognostic of PCa progression in the review and reanalysis, despite significant heterogeneity between studies, and thus present as promising prognostic biomarkers for PCa progression.

### miR-148a-3p

MiR-148a-3p is one of the most commonly dysregulated miRs in human cancers. Its downregulation has been observed in various cancers such as bladder, oesophageal, gastric, breast, colorectal and ovarian cancers [64–70]. Upregulation of miR-148a has also been detected in osteocarcinoma and glioblastoma [71, 72]. In PCa, upregulation of miR-148a-3p levels has been shown in prostate tumour tissue in comparison to adjacent normal tissue [33]. Upregulation was also observed in serum and urine of PCa patients in comparison to healthy controls [40, 73]. In contrast, levels of miR-148a-3p have been reported as lower in CRPC cell lines PC3 and DU145 compared to lines representing therapy-responsive disease [74, 75]. Similarly, in PCa patients, downregulation of the miR has been reported in CRPC cases compared to BPH cases and in high-grade tumours compared to low-grade tumours [32, 76].

Although expression of miR-148a-3p is variably reported in the literature, studies investigating its biological role in PCa generally suggest a tumour suppressive role. Sengupta et al. showed downregulation of miR-148a-3p in CRPC and identified DNA methylatransferase *DNMT1*, a gene upregulated in several cancers, as a target of the miR [75]. They reported that the two molecules exhibit a negative loop in PCa: while *DNMT1* enzyme methylates the miR promoter and silences miR expression, miR148a directly targets *DNMT1*, whose repression leads to induction of apoptosis and repression of cell proliferation and migration. They also demonstrated that ectopic expression of miR-148a-3p repressed anti-apoptotic *BCL2* in PC3 cells promoting apoptosis. Suppression of *DNMT1* by miR-148-3p has been reported in pancreatic, liver, bladder, oesophageal and gastric cancers [64–67, 77, 78]. Targeting of *BCL2* by miR-148a-3p has also been reported in colorectal and pancreatic cancers [69, 79]. Additionally, a study by Fujita et al. showed miR-148a-3p expression increased chemosensitivity in PC3 cells by directly targeting mitogen-and stress-activated protein kinase, *MSK1* [74]. These studies demonstrate that miR-148a-3p plays a role in promoting an anti-survival, tumour suppressive phenotype via similar mechanisms in various cancers including PCa and its loss is not only a good indicator of tumour progression but also shows potential to serve as a biomarker for therapeutic response in PCa.

### miR-582-5p

Similar to miR-148a-3p, miR-582-5p is reported to act as both an oncogene and a tumour suppressor in various cancers. In gastric, bladder, non-small cell lung cancers and endometrial carcinoma, miR582-5p levels are downregulated and shown to suppress proliferation, migration, invasion and promote apoptosis [80–83]. Conversely, in colorectal cancer and pituitary adenomas, it is over-expressed and promotes proliferation [84, 85]. The clinical significance of miR-582-5p in PCa is not yet elucidated and the literature presents conflicting evidence. The most recent research on miR-582-5p in PCa investigated its role in promoting bone metastasis; lower miR-582-5p expression was reported in PCa tissues with bone metastasis compared to PCa tissues without bone metastasis [61]. The study reported that lower miR-582-5p expression was significantly associated with shorter bone metastasis-free survival. They also demonstrated that over-expression of the miR in mice bearing PC3 tumour xenografts repressed bone metastasis and over-expression in PCa cell lines PC3, VCaP and C42B repressed cell invasion and migration. Mechanistically, the study proposed that miR-582-3p exerted its anti-invasion and migration properties by directly inhibiting components of the TGFβ signalling pathway (*SMAD2*, *TGFBRI* and *TGFBRII*) and subsequently the pathway itself. In a separate study, Maeno et al. developed an AR-positive, androgen-independent xenograft model KUCaP2 and cell line AILNCaP#1, and observed upregulation of miR-582-5p in these models in comparison to their androgen dependent counterparts [86]. They also demonstrated that suppression of the miR decreased cell proliferation in AILNCaP#1, suggesting an oncomiRic role of miR-582-5p in the transition of PCa from hormonesensitive to more aggressive castration-resistant phenotypes. These limited studies on miR-582-3p report conflicting roles in tumour progression, which indicate a dual role of the miR at different stages of progression from invasion and metastasis to the bone, to transition from androgen-dependent to aggressive CRPC. Given the identification of miR-582-5p as a potential prognostic candidate for PCa, further research into its exact role in PCa tumour progression is warranted.

### Limitations

One of the major issues highlighted by this study is the inconsistent findings between studies and datasets despite their common aim to identify prognostic miR biomarkers in PCa. These inconsistencies are mainly due to clinical and methodological heterogeneities that can arise at many points during the study. Due to the nature of retrospective cohort studies, clinical heterogeneity which encompasses factors such as race, family history, co-morbidity, treatment history, time to outcome and loss of follow up, was unavoidable. In the systematic review, a potential contributor to clinical heterogeneity was outcome endpoints. There were 12 different endpoints included in the systematic review as surrogates of disease progression. Further, many of the studies did not provide endpoint definitions. For studies that considered the same endpoints and provided endpoint definitions, definition heterogeneity still existed. This is evident in studies by Hulf et al. [87] and Nordby et al. [88] oth of which examined the association of miR-205 with BPFS but used different criteria to define BPFS. To minimise clinical heterogeneity in the reanalysis, we only used studies examining association of miRs with endpoint BPFS and samples originating from tumour tissues of patients who underwent RP and no other curative treatment.

It should be noted that BPFS and other PSA-based endpoints may not be ideal surrogate endpoints for disease progression. The ICECaP study, a large meta-analysis that aimed to determine clinically relevant endpoints for localised PCa, determined metastasis-free survival (MFS) as the most appropriate surrogate for PCa specific survival [89, 90]. However, BCR has been shown to be associated with increased risk of PCa metastasis and cancer-specific mortality and further is the most common endpoint recorded and reported in publications [7–11]. This is evident in the systematic review where almost half the studies (44%) considered BPFS, while only 6% of studies considered bone-/metastasis free survival, as endpoints. Moving forward, studies should consider evidence-based clinically relevant endpoints for studies focusing on disease progression. Similar to miR expression profile after RP, miR expression profiles also change at recurrence after radical radiotherapy, another curative treatment option for primary PCa. Several studies have identified miRs that change in expression in response to radiation and miRs that are involved in regulation of radiosensitivity in PCa [91–95]. It would be interesting to investigate and compare miR expression profile at recurrence after different curative treatments and their association with MFS [96–98].

Methodological heterogeneity, due to differences in study design, sample preparation methods, sample types, profiling technologies and threshold values for a positive result, was also present in the analyses. Besides these factors, one of the sources of methodological heterogeneity that may have influenced results were the different statistical tests (KM analysis or Cox PH regression) performed by different studies. The KM analysis only allows categorical variables as predictors, which can lead to weakening or loss of potential signal. It also cannot adjust to multiple predictors. Cox PH regression, on the other hand, is more flexible and allows for both categorical and continuous variables as predictors. Multiple predictors can also be added into a Cox PH model, allowing for adjustment of confounding variables. For this reason, when a study in the systematic review reported outcomes of both Cox PH and KM analyses, only the Cox PH results were extracted. However, even with adjustment for confounders in the Cox PH regression, there was potential for further heterogeneity to be introduced as different studies adjusted for different confounders. For example, Amankwah et al. [99], MelboJorgensen et al. [100] and Guan et al. [101] examined the association of miR-21-5p with progression using a multivariate Cox PH model, but each study considered different confounders in their model (Supplementary Table S2). Although appropriate measures were taken to reduce heterogeneity, it cannot be completely eliminated. This highlights the need for standardization of methodology and protocols in the field of biomarker discovery in order to derive more accurate conclusions from future investigations.

Overall, the minimisation of heterogeneity will require cannot be completely eliminated. This calls for the need for standardization of methodology and protocols at the pre-analytical level (such as sample collection, storage, profiling techniques and reagents used) and at the post-analytical level (such as data normalisation, processing tools/ pipelines and statistical analyses) in order to reduce as much technical variability as possible and facilitate more accurate conclusions from future investigations. Prospective studies with well- and pre-defined experimental design and research questions need to be devised to address clinical heterogeneity.

Besides heterogeneity, another major limitation in the systematic reanalysis was the limited number of publicly available datasets. Numerous studies generate novel miR expression data, but most do not make their data publicly available. This led to the inclusion of only six datasets for the systematic reanalysis. Additionally, the studies included in the reanalysis had a class imbalance problem whereby the proportion of samples that experienced the outcome were disproportionately lower than the samples that did not (Table S5). Insufficient datasets and class imbalance is a major problem of working with biomedical data, reducing the power of the study and potentially leading to biased conclusions specific to the cohorts in the analyses rather than the general population. Our research prompts for a more transparent system whereby researchers make their datasets available to other researchers. This will not only be useful for providing external validation cohorts but will also allow for scrutiny of the methodologies and analyses employed, which will improve scientific rigour and expedite biomarker discovery research.

The bottleneck in miR biomarker discovery seems to be the analytical and clinical validation step; this is highlighted in our results where we identified methodological heterogeneity between studies and lack of clinical validation in independent studies. Given the requirement for high quality and high-volume data, these are major challenges hindering progress in the field; addressing them is paramount for expediting miR biomarker research and successful translation of miR markers from the bench to the clinic.

## Conclusion

This is the first systematic review and only the second meta-analysis of miR profile data, updated and expanded with newer datasets and larger sample sizes compared to the first meta-analysis performed in 2017 [43], to focus on prognostic miR markers in PCa. It reveals considerable research undertaken in the field of biomarker discovery in PCa and reports all credible prognostic miRs reported so far. These findings present a valuable reference point for future studies and will be useful for regrouping strategies in investigations into prognostic miR biomarker research. This investigation also highlighted the lack of validation or inconsistent evidence for miRs frequently suggested to have prognostic biomarker potential. Only miR-148a-3p and miR-582-5p were consistently associated with disease progression in multiple publications and datasets, indicating reliability in predicting prognosis. Nevertheless, their biological significance in PCa progression is still uncertain. Further research to verify the biological roles of these miRs is warranted to support investigations into their performance as prognostic PCa biomarkers.

## Supplementary information

Supplemental Tables

## Data Availability

All data analysed in the study are publicly available, no new data were generated.
