# Supplementary material for: MicroRNAs as biomarkers for prostate cancer prognosis: a systematic review and a systematic reanalysis of public data
Source: Br J Cancer. 2022 Jan 12;126(3):502–13. doi: 10.1038/s41416-021-01677-3 (PMC8810870; doi:10.1038/s41416-021-01677-3)
Supplement: Supplementary file 1 — Supplemental Tables [file 41416_2021_1677_MOESM1_ESM.pdf]

Supplementary Tables

Prognostic miR	Prognostic test: test: endpoint	Prognostic test: HR (95% CI)	Prognostic test: p	Reference group	Association after reference standardisation	Sample size	Sample type	PMID	Ref
let-7b-5p	multivariate: BPFS	0.44 (0.193-1.022)	0.05	low	negative	98 (cohort A)	tissue	23798998	[1]
	multivariate: BPFS	0.30 (0.15-0.61)	<0.010	low	negative	92 (cohort B)	tissue		
	multivariate: CFFS	0.23 (0.08-0.70)	<0.010	low	negative	92 (cohort B)	tissue		
	multivariate: CFFS	0.46 (0.15-1.41)	0.17	low	negative	98 (cohort A)	tissue		
let-7c	multivariate: CFFS	0.53 (0.19-1.48)	0.22	low	negative	98 (cohort A)	tissue	23798998	[1]
miR-1-3p	univariate/ KM: DFS	0.29 (0.10-0.90)	0.008	low	negative	99 (MSKCC)	tissue	22210864	[2]
miR-100-5p	multivariate: BPFS	3.65 (1.38-9.62)	0.009	low	positive	49	tissue	21255804	[3]
miR-103a-3p	multivariate: BPFS	0.41 (0.21-0.79)	0.008	low	negative	93	serum	24583788	[4]
miR-106b-5p	univariate/ KM: DFS	2.70 (1.10-7.30)	0.014	low	positive	113 (MSKCC)	tissue	22986525	[5]
miR-10b-5p	multivariate: BPFS	2.15 (1.02-4.51)	0.044	low	positive	52	tissue	21769427	[6]
miR-1193	multivariate: BPFS	5.00 (1.60-15.60)	0.006	low	positive	43	tissue	25760964	[7]
miR-1207-3p	multivariate: RFS	2.50 (1.60-4.00)	<0.010	low	positive	368	tissue	27267842	[8]
	multivariate: CSS	1.80 (0.80-4.30)	0.060	low	positive	368	tissue		
miR-1231	multivariate: OS	2.17 (1.03-4.56)	0.041	low	positive	118	tissue	31822000	[9]
miR-125b-5p	multivariate: BPFS	1.79 (1.10-2.91)	0.018	low	positive	93	serum	24583788	[4]
	multivariate: BPFS	0.23 (0.01-3.75)	0.304	low	negative	76	tissue	19676045	[10]
miR-126-3p	multivariate: BPFS	3.68 (0.99-6.83)	0.010	high	negative	128	tissue	24350576	[11]
miR-128-3p	multivariate: BPFS	3.96 (1.02-8.12)	0.010	high	negative	128	tissue	26339409	[12]
	multivariate: BPFS	3.32 (0.92-6.91)	0.010	high	negative	128	serum		
miR-129-5p	multivariate: OS	2.77 (0.93-8.17)	0.048	high	negative	84	peripheral blood	31788094	[13]
	multivariate: DSS	6.12 (1.56-24.07)	0.009	low methylation	positive	180 (cohort 1)	tissue	28143614	[14]
miR-1290	univariate: OS	8.04 (2.36-27.33)	<0.001	high	negative	100	exosome (blood)	25129854	[15]
miR-1303	KM: OS	-	0.031	high	positive	30	tissue	31772644	[16]
miR-130b-3p	multivariate: OS	22.4 (2.30-222.40)	0.008	high	negative	36 (African Americans)	tissue	31266828	[17]

	multivariate: OS	1.1 (0.10-8.30)	0.910	high	negative	57 (European American)	tissue		
miR-133a-3p	multivariate: bone MFS	0.37 (0.19-0.74)	0.005	low	negative	223 (TCGA)	tissue	30021600	[18]
	multivariate: OS	1.07 (0.41-2.78)	0.886	low	positive	245 (TCGA)	tissue		
miR-133b	multivariate: BPFS	1.78 (1.01-3.11)	0.040	low	positive	135	tissue	24610824	[19]
	univariate: bone MFS	0.08 (0.03-0.22)	0.001	low	negative	176	tissue	30006541	[20]
	multivariate: OS	6.90 (0.72-66.60)	0.095	low	positive	202	tissue		
miR-139-5p	multivariate: OS	0.25 (0.07-0.93)	0.038	high	positive	84	peripheral blood	31788094	[13]
	multivariate: BPFS	0.77 (0.58-1.04)	0.091	low	negative	540	tissue	31269290	[21]
	multivariate: MFS	0.60 (0.28-1.28)	0.188	low	negative	540	tissue		
miR-141-3p	multivariate: BPFS	1.07 (1.00-1.14)	0.05	low	positive	463	epithelial and stromal	30674952	[22]
	multivariate: BPFS	1.92 (1.32-2.79)	0.001	low	positive	207	tissue	31640261	[23]
miR-143-5p	univariate: RFS	0.32 (0.12-0.81)	0.016	low	negative	49	tissue	28628624	[24]
	KM: OS	-	0.047	high	positive	n/s (TCGA)	tissue	30027097	[25]
miR-145-5p	multivariate: BPFS	4.47 (1.27-15.74)	0.020	high	negative	36 (low + intermediate risk)	tissue	23703249	[26]
	multivariate: BPFS	4.43 (1.11-17.61)	0.035	high	negative	29 (intermediate risk)	tissue		
	multivariate: PFS	0.40 (0.17-0.94)	0.036	low	negative	106	tissue	20332243	[27]
	univariate/ KM: OS	3.00 (1.60-7.00)	<0.010	high	negative	49	tissue	25969144	[28]
	univariate: BPS	0.74 (0.23-2.34)	0.609	low	negative	76	tissue	19676045	[10]
	univariate: BPFS	0.68 (0.22-2.14)	0.510	low	negative	73	tissue	22864280	[29]
	multivariate: DFS	1.26 (0.49-3.27)	0.629	high	negative	73	tissue	23703249	[26]
miR-146-3p	multivariate: BPFS	1.16 (1.03-1.31)	0.017	low	positive	16	serum	23846169	[30]
miR-146a-5p	KM: BPFS	-	0.048	low	negative	98 (MSKCC)	tissue	26306811	[31]
miR-146a-5p rs2910164	multivariate: BPFS	0.83 (0.30-2.32)	0.722	CC vs GG/GC	negative	72	peripheral blood	25526182	[32]
miR-148a-3p	multivariate: BPFS	0.60 (0.44-0.81)	0.001	low	negative	207	tissue	31640261	[23]
miR-149-5p	multivariate: BPFS	0.68 (0.22-2.15)	0.510	low	negative	76	tissue	19676045	[10]
miR-150-5p	KM: OS	-	0.035	low	negative	86	tissue	30009782	[33]

miR-152-3p	KM: BPFS	-	<0.001	low	negative	n/s (MSKCC)	tissue	25004396	[34]
	multivariate: DFS	0.23 (0.07-0.72)	0.012	low	negative	494 (TCGA)	tissue	29599847	[35]
miR-153	multivariate: OS	2.48 (1.58-10.73)	0.019	low	positive	143	tissue	31490362	[36]
miR-15b-5p	univariate: OS	0.69 (0.14-3.51)	0.658	high	positive	387 (TCGA)	tissue	29363862	[37]
	multivariate: RFS	1.16 (0.68-1.98)	0.583	high	negative	387 (TCGA)	tissue		
miR-16-5p	KM: BPFS	-	0.003	low	negative	n/s (MSKCC)	tissue	30032144	[38]
	multivariate: BPFS	48.4 (1.26-1858)	0.037	low	positive	76	tissue	19676045	[10]
miR-17-5p	KM: BPFS	-	0.013	high	positive	268 (TCGA)	tissue	31122242	[39]
miR-181b-5p	multivariate: BPFS	1.97 (0.21-18.70)	0.553	low	positive	76	tissue	19676045	[10]
miR-182-3p	multivariate: BPFS	0.43 (0.01-17.80)	0.658	low	negative	76	tissue	19676045	[10]
miR-182-5p	KM: OS	-	0.002	high	positive	63	tissue	27179774	[40]
	KM: OS	-	0.012	high	positive	52	tissue	23383207	[41]
	multivariate: BPFS	2.00 (1.00-3.50)	0.009	low	positive	204	tissue	24518785	[42]
	multivariate: PFS	2.50 (1.00-5.00)	0.013	low	positive	204	tissue		
	univariate: BPFS	1.50 (1.00-2.00)	0.147	low	positive	137	tissue		
	multivariate: PFS	1 (0.50-2.00)	0.387	low	positive	137	tissue		
	multivariate: BPFS	0.36 (0.01-17.90)	0.608	low	negative	76	tissue	19676045	[10]
miR-183-3p	KM: OS	-	0.001	high	positive	n/s (TCGA)	tissue	30027097	[25]
	multivariate: BPFS	27.40 (0.46-1622)	0.112	low	positive	76	tissue	19676045	[10]
miR-184	multivariate: BPFS	3.48 (0.16-73.90)	0.423	low	positive	76	tissue	19676045	[10]
miR-186-5p	KM: PS	-	0.028	low	negative	38	tissue	27121312	[43]
miR-188-5p	multivariate: BPFS	2.11 (1.34-3.33)	0.001	high	negative	180	tissue	25714029	[44]
	multivariate: OS	3.01 (1.74-5.21)	<0.001	high	negative	180	tissue		
miR-190a	KM: DFS	-	0.035	low	negative	35	tissue	26314494	[45]
miR-191-5p	multivariate: OS	2.31 (1.67-9.01)	0.027	low	positive	146	tissue	31335671	[46]
miR-192-5p	KM: BPFS	-	0.007	high	positive	n/s (TCGA)	tissue	30544100	[47]
miR-194-5p	multivariate: BPFS	1.08 (0.91-1.28)	0.399	low	positive	16	serum	23846169	[30]
miR-195-5p	multivariate: BPFS	5.96 (1.18-30.02)	0.031	high	negative	140	tissue	26338045	[48]
	multivariate: OS	4.46 (1.35-14.72)	0.014	high	negative	140	tissue		
	multivariate: BPFS	0.61 (0.41-0.93)	0.022	low	negative	107 (MSKCC)	tissue	26080838	[49]
	KM: BPFS	-	0.009	low	negative	131 (MSKCC)	tissue	30032144	[38]
	KM: RFS	-	0.049	low	negative	98 (MSKCC)	tissue	26650737	[50]
	KM: DFS	-	<0.010	low	negative	n/s (MSKCC)	tissue	27175617	[51]
miR-19a-3p	KM: BPFS	-	0.034	high	positive	328 (TCGA)	tissue	29416742	[52]

miR-200b-3p	KM: BPFS	-	0.049	low	negative	51	tissue	25409297	[53]
miR-203a-3p	univariate/ KM: PS	2.52 (1.14-5.55)	0.023	low	positive	44	tissue	26499781	[54]
miR-204-5p	KM: bone MFS	0.25 (0.14-0.48)	<0.001	low	negative	136	serum	31678733	[55]
miR-205-5p	multivariate: BPFS	1.70 (1.23-2.36)	0.001	high	negative	535	tissue	29176717	[56]
	univariate/ KM: OS	2.33 (1.11-4.88)	0.030	high	negative	49	tissue	23571738	[57]
	multivariate: BPFS	2.23 (0.99-5.00)	0.05	low methylation	negative	149	tissue	22869146	[58]
	multivariate: CSS	6.88 (1.66-28.53)	0.001	high	negative	105 (cohort train)	tissue	24173237	[59]
	multivariate: CSS	6.55 (1.29-33.10)	0.023	high	negative	78 (cohort validation)	tissue		
	multivariate: BPFS	1.96 (0.80-4.80)	0.141	high	negative	105 (cohort train)	tissue		
	multivariate: CFFS	0.86 (0.08-9.16)	0.900	high	positive	78 (cohort validation)	tissue		
	multivariate: BPFS	0.76 (0.35-2.62)	0.472	high	positive	78 (cohort validation)	tissue		
	univariate: BPFS	0.37 (0.10-1.40)	0.128	low	negative	76	tissue	19676045	[10]
miR-20a-5p	KM: BPFS	-	<0.001	high	positive	268 (TCGA)	tissue	31122242	[39]
miR-20b-5p	KM: BPFS	-	0.180	high	positive	268 (TCGA)	tissue	31122242	[39]
miR-21-5p	multivariate: BPFS	2.40 (1.06-5.49)	0.037	low	positive	167 (Gleason == 6)	stromal	25401698	[60]
	multivariate: BPFS	1.40 (1.0-1.90)	0.089	low	positive	170 (all cohort)	stromal		
	multivariate: BPFS	2.06 (1.08-3.94)	0.029	low	positive	168	tissue	22341810	[61]
	multivariate: PFS	1.99 (1.03-3.82)	0.040	low	positive	85	tissue	27040772	[62]
	multivariate: RFS	6.15 (1.04-36.48)	0.045	high	negative	65 (obese)	tissue	23353719	[63]
	multivariate: RFS	1.99 (0.70-5.64)	0.200	high	negative	65 (obese + non-obese)	tissue		
	multivariate: RFS	1.28 (0.3-5.49)	0.740	high	negative	45 (non-obese)	tissue		
miR-210-3p	multivariate: CFFS	2.76 (1.25-6.09)	0.012	low	positive	535	fibroblast	27824162	[64]
miR-212-3p	KM: PS	-	<0.050	low	negative	72	tissue	29917185	[65]
miR-218-5p	univariate/ KM: bone MFS	0.44 (0.21-0.90)	0.015	low	negative	107	serum	30870834	[66]
	univariate/ KM: bone MFS	0.38 (0.19-0.78)	0.009	low	negative	107	tissue		
	univariate/ KM: OS	0.82 (0.19-3.62)	0.875	low	negative	109	serum		
	univariate/ KM: OS	0.79 (0.18-3.38)	0.757	low	negative	109	serum		

miR-221-3p	multivariate: CRFS	0.53 (0.29-0.95)	0.032	low	negative	92	tissue	19585579	[67]
	KM: CRPC FS	-	0.012	high	positive	45 (Gleason >= 8)	whole blood	24760272	[68]
	multivariate: BPFS	0.74 (0.61-0.90)	0.002	low	negative	207	tissue	31640261	[23]
	univariate: BPFS	0.36 (0.17-1.90)	0.570	low	negative	73	tissue	22864280	[29]
	KM: CRPC FS	-	0.147	low	negative	52	tissue	26325107	[69]
	multivariate: BPFS	0.5 (0.01-39.10)	0.757	low	negative	76	tissue	19676045	[10]
	multivariate: RFS	0.56 (0.21-1.50)	0.250	high	positive	63 (all cases)	tissue	23353719	[63]
	multivariate: RFS	0.40 (0.09-1.84)	0.240	high	positive	44 (non-obese)	tissue		
	multivariate: RFS	0.46 (0.10-2.22)	0.330	high	positive	19 (obese)	tissue		
miR-222-3p	multivariate: BPFS	2.80 (1.29-6.20)	0.009	low	positive	93	serum	24583788	[4]
	multivariate: CRPC FS	0.21 (0.07-0.64)	0.006	low	negative	52	tissue	26325107	[69]
	multivariate: BPFS	5.04 (0.03-940)	0.544	low	positive	76	tissue	19676045	[10]
	multivariate: RFS	0.39 (0.14-1.15)	0.090	high	positive	60 (all cases)	tissue	23353719	[63]
	multivariate: RFS	0.37 (0.09-1.59)	0.180	high	positive	42 (non-obese)	tissue		
	multivariate: RFS	0.46 (0.07-3.19)	0.440	high	positive	18 (obese)	tissue		
miR-224-5p	multivariate: BPFS	0.25 (0.08-0.74)	0.010	low	negative	114	tissue	24382668	[70]
	multivariate: BPFS	0.64 (0.14-2.39)	0.525	low	negative	58	tissue	23136246	[71]
miR-23a-3p	multivariate: PS	1.78 (1.12-2.83)	0.015	low	positive	123	tissue	25714010	[72]
miR-23a-3p rs3745453	multivariate: OS	9.67 (2.83-33.09)	0.001	CT/TT vs CC	positive	156	peripheral blood	31876746	[73]
miR-23b-3p	univariate/ KM: OS	8.10 (4.00-19.00)	<0.001	high	negative	151	tissue	23074286	[74]
	univariate/ KM: RFS	6.20 (3.00-13.00)	<0.001	high	negative	151	tissue		
	KM: OS	-	0.042	high	positive	n/s (TCGA)	tissue	30027097	[25]
miR-26a-3p	KM: OS	-	0.038	low	negative	140	tissue	27449037	[75]
miR-27a-3p	KM: PS	-	<0.050	high	positive	60	serum	30250598	[76]
miR-27b-3p	multivariate: CRPC FS	0.26 (0.07-0.94)	0.041	low	negative	49	tissue	25115396	[77]
miR-301a-3p	multivariate: BPFS	1.42 (1.06-1.90)	0.019	low	positive	609	tissue	26990571	[78]
miR-30c-5p	multivariate: BPFS	0.34 (0.17-0.68)	0.002	low	negative	103	tissue	24452717	[79]
	univariate: PS	2.38 (1.09-5.22)	0.015	low	positive	44	tissue	26499781	[54]
	multivariate: BPFS	0.49 (0.28-0.85)	0.011	low	negative	207	tissue	31640261	[23]
miR-30d-5p	multivariate: BPFS	5.93 (1.75-20.09)	0.003	low	positive	56	tissue	23231923	[80]

	multivariate: BPFS	1.94 (0.71-5.29)	0.198	high	negative	113 (MSKCC)	tissue	28241827	[81]
miR-31-5p	multivariate: BPFS	0.78 (0.67-0.91)	0.001	low	negative	207	tissue	31640261	[23]
	multivariate: BPFS	15 (0.19-1179)	0.224	low	positive	76	tissue	19676045	[10]
miR-320e	multivariate: BPFS	3.20 (1.10-9.60)	0.034	low	positive	43	tissue	25760964	[7]
miR-326	KM: OS	-	0.027	low	negative	58	tissue	30243091	[82]
	KM: BPFS	-	0.020	low	negative	58	tissue		
miR-335-5p	KM: OS	-	0.339	low	negative	20	tissue	23456549	[83]
	KM: MFS	-	0.185	low	negative	20	tissue		
	KM: BPFS	-	0.713	low	negative	20	tissue		
miR-338-3p	univariate/ KM: BPFS	0.78 (0.54-1.14)	0.020	low	negative	25 (MSKCC)	tissue	26907180	[84]
miR-34b-3p	univariate/ KM: BPFS	3.30 (1.30-8.70)	0.020	high	negative	74	tissue	23147995	[85]
miR-34b/c	multivariate: DFS	2.76 (1.24-6.15)	0.013	low methylation	negative	74	tissue	28143614	[14]
	multivariate: DSS	3.84 (1.27-11.60)	0.017						
miR-34c-5p	KM: PS	-	<0.001	low	negative	49	tissue	21351256	[86]
miR-3607-5p	KM: PS	-	0.046	low	negative	100	tissue	24817628	[87]
miR-3622a-5p	KM: OS	-	0.049	low	negative	124 (TCGA)	tissue	28498363	[88]
miR-3622b-5p	KM: BPFS	-	0.032	low	negative	124	tissue	27611943	[89]
	KM: OS	-	0.262	low	negative	94	tissue		
miR-373-3p	KM: OS	-	0.038	low	negative	56	tissue	30338790	[90]
miR-374b-5p	multivariate: BPFS	0.38 (0.17-0.85)	0.018	low	negative	99	tissue	24191917	[91]
miR-375	univariate: OS	2.69 (1.52-4.77)	<0.001	low	positive	100	exosomes (blood)	25129854	[15]
	multivariate: BPFS	0.42 (0.03-5.60)	0.544	low	negative	76	tissue	19676045	[10]
miR-378-3p	multivariate: DFS	4.79 (1.31-15.52)	0.018	gain vs loss	negative	27 (high + v. high risk)	tissue	25153390	[92]
	multivariate: DFS	1.72 (0.82-3.63)	0.152	gain vs loss	negative	26	tissue		
miR-379-5p	KM: DFS	-	0.012	high	positive	107 (MSKCC)	tissue	25324143	[93]
miR-409-3p	KM: DFS	-	<0.001	high	positive	107 (MSKCC)	tissue	24963047	[94]
miR-410-3p	KM: OS	-	0.011	high	positive	82	tissue	29969630	[95]
miR-423-3p rs6505162	multivariate: CSS	0.64 (0.40-1.01)	0.054	CC vs CA/AA	negative	601	peripheral blood	21149617	[96]
miR-424-3p	multivariate: CFFS	0.44 (0.22-0.87)	0.018	low	negative	404	tissue	31337863	[97]

miR-4288	KM: OS	-	0.070	low	negative	74	tissue	30874288	[98]
miR-4319	KM: OS	-	<0.050	low	negative	40	tissue	29633185	[99]
miR-449b-5p	multivariate: BPFS	1.90 (1.25-2.85)	0.003	low	positive	163	tissue	25416653	[100]
miR-4516	multivariate: BPFS	3.60 (1.30-10.00)	0.013	low	positive	43	tissue	25760964	[7]
miR-452-5p	KM: CRPC FS	-	0.041	low	negative	52	tissue	27070713	[101]
miR-4534	univariate/ KM: OS	6.00 (3.00-17.00)	0.040	low	positive	84	tissue	27634912	[102]
miR-455-5p	KM: RFS	-	0.006	low	negative	107 (MSKCC)	tissue	31111062	[103]
miR-466	KM: RFS	-	0.010	low	negative	75	tissue	28125091	[104]
miR-4723-5p	KM: PS	-	0.043	low	negative	57	tissue	24223753	[105]
miR-500a-5p	KM: OS	-	<0.050	high	positive	148	tissue	28631332	[106]
miR-503-5p	KM: PS	-	<0.010	low	negative	82	tissue	27267060	[107]
miR-505-3p	univariate/ KM: bone MFS	0.25 (0.12-0.56)	0.002	low	negative	81	tissue	30365141	[108]
	univariate/ KM: OS	0.50 (0.10-2.46)	0.002	low	negative	127	tissue		
miR-508-3p	multivariate: BPFS	3.00 (1.10-8.00)	0.030	low	positive	43	tissue	25760964	[7]
miR-515-5p	KM: OS	-	0.018	low	negative	96	tissue	30685303	[109]
miR-548c-3p	KM: RFS	-	0.039	low	negative	n/s (MSKCC)	tissue	25234358	[110]
miR-563	multivariate: BPFS	0.30 (0.10-0.80)	0.023	low	negative	43	tissue	25760964	[7]
miR-573	KM: MFS	-	0.041	low	negative	55	tissue	26451614	[111]
miR-582-3p	univariate/ KM: bone MFS	0.31 (0.15-0.66)	0.002	low	negative	94 (TCGA)	tissue	30852380	[112]
	univariate/ KM: OS	0.43 (0.19-1.88)	0.26	low	negative	157 (TCGA)	tissue		
miR-582-5p	univariate/ KM: bone MFS	0.21 (0.10-0.45)	<0.001	low	negative	94 (TCGA)	tissue	30852380	[112]
	univariate/ KM: OS	0.74 (0.17-3.27)	0.696	low	negative	157 (TCGA)	tissue		
miR-598	multivariate: BPFS	0.30 (0.10-0.90)	0.030	low	negative	43	tissue	25760964	[7]
miR-601	multivariate: BPFS	4.60 (1.60-12.70)	0.004	low	positive	43	tissue	25760964	[7]
miR-605 rs2043556	multivariate: BPFS	1.96 (1.16-3.30)	0.010	GG vs AA/AG	positive	846	peripheral blood	24740842	[113]
miR-615-3p	multivariate: CSS	2.66 (1.29-5.49)	0.008	low	positive	734 (cohort 1+2+3)	tissue	31539518	[114]
	multivariate: BPS	1.38 (0.84-2.26)	0.210	low	positive	239 (cohort 1)	tissue		
	multivariate: BPS	1.05 (0.67-1.66)	0.820	low	positive	222 (cohort 2)	tissue		
	multivariate: BPS	1.31 (0.86-2.01)	0.210	low	positive	273 (cohort 3)	tissue		

	multivariate: BPS	1.46 (0.78-2.73)	0.240	low	positive	387 (cohort 4)	tissue		
miR-626	multivariate: BPFS	0.30 (0.10-0.90)	0.039	low	negative	43	tissue	25760964	[7]
miR-628-3p	multivariate: BPFS	6.60 (1.90-23.50)	0.004	low	positive	43	tissue	25760964	[7]
miR-652-3p	multivariate: BPFS	1.47 (1.09-1.98)	0.013	low	positive	585	tissue	29721191	[115]
	multivariate: MFS	1.16 (0.54-2.48)	0.710	low	positive	585	tissue		
miR-663	multivariate: PFS	2.92 (1.98-4.32)	<0.001	low	positive	127	tissue	24243035	[116]
miR-7-5p	KM: CRPC FS	-	0.004	high	positive	45 (Gleason >= 8)	whole blood	24760272	[68]
miR-708-5p	KM: PS	6.00 (2.20-16.40)	0.006	high	negative	134	tissue	22552290	[117]
miR-744-5p	multivariate: BPFS	8.27 (1.85-37.06)	0.006	low	positive	98 (MSKCC)	tissue	28107193	[118]
miR-93-5p	KM: RFS	2.01 (1.49-2.71)	<0.001	low	positive	n/s (TCGA)	tissue	30582208	[119]
	KM: MFS	0.79 (0.65-0.57)	0.701	low	negative	n/s (TCGA)	tissue		
	KM: OS	2.11 (0.96-4.67)	0.064	low	positive	n/s (TCGA)	tissue		
miR-95-3p	KM: OS	-	0.012	high	positive	n/s (TCGA)	tissue	30027097	[25]
miR-96	KM: OS	2.20 (1.04-4.46)	0.039	low	positive	50	tissue	23951320	[120]
	multivariate: BPFS	3.91 (0.99-15.60)	0.053	low	positive	76	tissue	19676045	[10]
	uni: BPFS	0.71 (0.23-2.24)	0.560	low	negative	73	tissue	22864280	[29]

Table S1: **A table of all individual miRNAs that have been investigated for their prognostic potential in PCa so far, built by performing a systematic review of relevant publications in the Pubmed database.** KM, univariate and multivariate tests stand for Kaplan-Meier test, and univariate and multivariate Cox PH regressions respectively. For test entries "univariate/ KM", both univariate Cox PH and KM analysis were performed but there was no associated p-value for the Cox analysis. Thus the HR and 95% CI corresponds to outputs of the univariate Cox PH and the p-value corresponds to KM log-rank test. The values in the "Prognostic test" and "Reference group" columns refer to the statistics and the reference group used for comparison as reported in respective papers. In contrast, the "Association after reference standardisation" column refers to the association of the miRs to progression after standardising the comparisons to "low" miR expression as the reference group. Refer to Supplementary Table S2 for endpoint definitions and adjusted variables included in the multivariate analyses. Refer to Table 2 for the full form of the abbreviated endpoints. n/s represents not-specified.

PMID	miR	Endpoint	Endpoint definition	variables in multivariate Cox PH analysis					Ref
				Gleason	T stage	PSA	age	others	
23798998	let-7b-5p	BPFS	PSA $\geq$ 0.2 ng/ml on 2 consecutive follow-up visits	x	x	x			[1]
23798998	let-7b-5p	CFFS	clinical failure declared when either local or distant metastases histologically proven or confirmed by CT or bone scan	x	x	x			[1]
23798998	let-7c	CFFS	clinical failure declared when either local or distant metastases histologically proven or confirmed by CT or bone scan	x	x	x			[1]
22210864	miR-1-3p	DFS	no definition	x			x		[2]
21255804	miR-100-5p	BPFS	PSA $\geq$ 0.2 ng/ml			x		% tumour volume	[3]
24583788	miR-103a-3p	BPFS	serum PSA of 0.2 ng/mL or greater (obtained 6 weeks – 3 months post-operatively), with a second confirmatory level of PSA greater than 0.2 ng/mL	x		x	x	body-mass index	[4]
22986525	miR-106b-5p	DFS	no definition						[5]
21769427	miR-10b-5p	BPFS	the first post-operative PSA of >0.1 ng/ml, as confirmed by at least 1 subsequent increasing value (persistent PSA increase) after achieving undetectable PSA post-operatively, defined as a detection limit of <0.04 ng/ml	x	x	x		surgical margin status	[6]
25760964	miR-1193	BPFS	recurrence after salvage radiation at least twice consecutively following the nadir	x				lymph node status	[7]
27267842	miR-1207-3p	RFS	time from the date of PCa diagnosis to PCa recurrence or non-recurrence death, whichever comes first		x		x		[8]
27267842	miR-1207-3p	CSS	PCa death		x		x		[8]
31822000	miR-1231	OS	no definition	x	x	x	x	differentiation, lymph node met	[9]
24583788	miR-125b-5p	BPFS	serum PSA of 0.2 ng/mL or greater (obtained 6 weeks – 3 months postoperatively), with a second confirmatory level of PSA greater than 0.2 ng/mL (N=31, classified as progressors)	x		x	x	body-mass index	[4]

19676045	miR-125b-5p	BPFS	post-operative PSA value >0.1 lg/l confirmed by at least one subsequent rising value after the patients had reached an undetectable PSA level (detection limit <0.04 lg/l) after surgery	x	x	x	x	surgical margin status	[10]
24350576	miR-126-3p	BPFS	the period between surgical treatment and the measurement of two successive values of serum PSA level ≥ 0.2 ng/ml		x			lymph node met, angiolymphatic invasion	[11]
26339409	miR-128-3p	BPFS	the period between surgical treatment and the measurement of two successive values of serum PSA level ≥ 0.2 ng/ml		x			lymph node met, angiolymphatic invasion	[12]
31788094	miR-129-5p	OS	no definition	x	x	x		met,TNM,miR139	[13]
28143614	miR-129-5p	DSS	the time elapsed since diagnosis until death or the last follow-up	x					[14]
25129854	miR-1290	OS	no definition						[15]
31772644	miR-1303	OS	no definition						[16]
31266828	miR-130b-3p	OS	the time of surgery until time of the death or last follow-up	x	x	x	x	PSA failure	[17]
31266828	miR-130b-3p	OS	the time of surgery until time of the death or last follow-up	x	x	x	x		[17]
30021600	miR-133a-3p	bone MFS	no definition	x	x	x	x	lymph node status	[18]
30021600	miR-133a-3p	OS	no definition	x	x	x	x	lymph node status	[18]
24610824	miR-133b	BPFS	the time from the date of surgery to that of BCR (postoperative serum PSA concentration $\geq 0.\ 2$ ng/mL)	x		x		RB1CC1 gene, surgical tumour margins	[19]
30006541	miR-133b	bone MFS	no definition						[20]
30006541	miR-133b	OS	no definition	x	x	x	x	lymph node status	[20]
31788094	miR-139-5p	OS	overall survival rate	x	x	x		lymph node met, distant met, miR-129	[13]
31269290	miR-139-5p	BPFS	PSA increase of at least 0.2 ng/mL on at least two separate consecutive measurements that are at least 3 months apart	x	x	x	x	surgical margin status, lymph node status	[21]

31269290	miR-139-5p	MFS	lesions within the bone identified on radionuclide bone scan and lymphadenopathy or visceral lesions identified by computed tomography imaging of the abdomen, pelvis and chest	x	x	x			[21]
30674952	miR-141-3p	BPFS	the time from surgery to PSA threshold (no definition of PSA threshold given)	x	x		x	positive surgical margins, apical positive surgical margins, perineural infiltration	[22]
31640261	miR-141-3p	BPFS	no definition					miR-30c-5p, miR-30d-5p, miR-31-5p, miR-148a-3p, miR-221-3p	[23]
28628624	miR-143-5p	RFS	the time from surgery to BCR or death of any cause						[24]
30027097	miR-143-5p	OS	no definition						[25]
20332243	miR-145-5p	PFS	the time from definitive diagnosis to any of the following events after initial treatment: prostate-specific antigen elevation, local progression, metastasis, or disease-specific death as failure of treatment	x	x	x			[27]
23703249	miR-145-5p	BPFS	two consecutive measurements of serum PSA $\geq$ 0.2 ng/ml	x	x	x	x	digital rectal examination	[26]
23703249	miR-145-5p	BPFS	two consecutive measurements of serum PSA $\geq$ 0.2 ng/ml	x	x	x	x	digital rectal examination	[26]
25969144	miR-145-5p	OS	no definition						[28]
22864280	miR-145-5p	BPFS	PSA $\geq$ 0.2 ng ml at two consecutive follow-up visits						[29]
23703249	miR-145-5p	DFS	interval between the radical prostatectomy and the time of biochemical relapse, or the time period between the surgery and the most recent measurement of serum PSA for the patients who did not present biochemical recurrence	x	x	x	x	digital rectal examination	[26]

19676045	miR-145-5p	BPFS	post-operative PSA value >0.1 lg/l confirmed by at least one subsequent rising value after the patients had reached an undetectable PSA level (detection limit <0.04 lg/l) after surgery						[10]
23846169	miR-146-3p	BPFS	biochemical disease progression with a serum PSA concentration of 0.2 ng/ml increasing over a 3-month period	x	x	x		surgical margin status, seminal vesicle invasion	[30]
26306811	miR-146a-5p	BPFS	no definition						[31]
25526182	miR-146a-5p	BPFS	post-operative PSA level $\geq$ 0.2 ng/mL	x	x	x	x	positive surgical margins, perineural infiltration	[32]
31640261	miR-148a-3p	BPFS	no definition					miR-30c-5p, miR-30d-5p, miR-31-5p, miR-141-3p, miR-221-3p	[23]
19676045	miR-149-5p	BPFS	post-operative PSA value >0.1 lg/l confirmed by at least one subsequent rising value after the patients had reached an undetectable PSA level (detection limit <0.04 lg/l) after surgery						[10]
30009782	miR-150-5p	OS	no definition						[33]
25004396	miR-152-3p	BPFS	no definition						[34]
29599847	miR-152-3p	DFS	the date of the radical prostatectomy to the date of relapse, or date of last follow-up or death if relapse-free	x	x	x	x	surgical margin status, lymph node status	[35]
31490362	miR-153	OS	no definition	x	x	x	x	TNM staging, family history, lymph node met, bone met, type of surgery	[36]
29363862	miR-15b-5p	OS	no definition						[37]
29363862	miR-15b-5p	RFS	no definition	x	x	x			[37]
30032144	miR-16-5p	BPFS	no definition						[38]

19676045	miR-16-5p	BPFS	post-operative PSA value >0.1 lg/l confirmed by at least one subsequent rising value after the patients had reached an undetectable PSA level (detection limit <0.04 lg/l) after surgery	x	x	x	x	surgical margin status	[10]
31122242	miR-17-5p	BPFS	no definition						[39]
19676045	miR-181b-5p	BPFS	post-operative PSA value >0.1 lg/l confirmed by at least one subsequent rising value after the patients had reached an undetectable PSA level (detection limit <0.04 lg/l) after surgery	x	x	x	x	surgical margin status	[10]
19676045	miR-182-3p	BPFS	post-operative PSA value >0.1 lg/l confirmed by at least one subsequent rising value after the patients had reached an undetectable PSA level (detection limit <0.04 lg/l) after surgery	x	x	x	x	surgical margin status	[10]
27179774	miR-182-5p	OS	no definition						[40]
24518785	miR-182-5p	BPFS	PSA 0.4 ng/ml or greater during followup	x	x	x		lymph node status, surgical margin status	[42]
24518785	miR-182-5p	PFS	local (prostatic fossa), regional (lymph nodes) or distant (metastasis) progression	x	x	x		lymph node status, surgical margin status	[42]
23383207	miR-182-5p	OS	no definition						[41]
24518785	miR-182-5p	BPFS	PSA 0.4 ng/ml or greater during followup						[42]
24518785	miR-182-5p	PFS	local (prostatic fossa), regional (lymph nodes) or distant (metastasis) progression	x				surgical margin status	[42]
19676045	miR-182-5p	BPFS	post-operative PSA value >0.1 lg/l confirmed by at least one subsequent rising value after the patients had reached an undetectable PSA level (detection limit <0.04 lg/l) after surgery	x	x	x	x	surgical margin status	[10]
30027097	miR-183-3p	OS	no definition						[25]
19676045	miR-183-3p	BPFS	post-operative PSA value >0.1 lg/l confirmed by at least one subsequent rising value after the patients had reached an undetectable PSA level (detection limit <0.04 lg/l) after surgery	x	x	x	x	surgical margin status	[10]

19676045	MiR-184	BPFS	post-operative PSA value >0.1 lg/l confirmed by at least one subsequent rising value after the patients had reached an undetectable PSA level (detection limit <0.04 lg/l) after surgery	x	x	x	x	surgical margin status	[10]
27121312	miR-186-5p	PS	patient survival						[43]
25714029	miR-188-5p	BPFS	the period between surgical treatment and the measurement of two successive values of serum PSA level $\geq$ 0.2 ng/ml	x		x		seminal vesicle invasion	[44]
25714029	miR-188-5p	OS	no definition	x	x	x			[44]
26314494	miR-190a	DFS	no definition						[45]
31335671	miR-191-5p	OS	no definition	x	x	x	x	pelvic lymph node met, bone met, surgical margin status	[46]
30544100	miR-192-5p	BPFS	no definition						[47]
23846169	miR-194-5p	BPFS	biochemical disease progression with a serum PSA concentration of 0.2 ng/ml increasing over a 3-month period	x	x	x		surgical margin status, seminal vesicle invasion	[30]
26080838	miR-195-5p	BPFS	no definition	x	x	x	x		[49]
26650737	miR-195-5p	RFS	no definition						[50]
27175617	miR-195-5p	DFS	no definition						[51]
30032144	miR-195-5p	BPFS	no definition						[38]
26338045	miR-195-5p	BPFS	no definition	x				lymph node met	[48]
26338045	miR-195-5p	OS	no definition	x	x				[48]
29416742	miR-19a-3p	BPFS	no definition						[52]
25409297	miR-200b-3p	BPFS	PSA >0.02 ng/mL						[53]
26499781	miR-203a-3p	PS	no definition						[54]
31678733	miR-204-5p	bone MFS	no definition						[55]
22869146	miR-205-5p	BPFS	biochemical disease progression with a serum PSA concentration $\geq$ 0.2ng/ml increasing over a 3-month period or local recurrence on digital rectal examination confirmed by biopsy or by a subsequent rise in PSA	x	x				[58]

29176717	miR-205-5p	BPFS	Post-operative PSA $\geq$ 0.4 or intervention with salvage therapy					CAPRA-S score, tumour size, perineural infiltration, lymphovascular infiltration	[56]
23571738	miR-205-5p	OS	no definition						[57]
24173237	miR-205-5p	CSS	PCa specific death	x	x				[59]
24173237	miR-205-5p	BPFS	PSA $\geq$ 0.2 ng/mL on two consecutive follow-up visits	x	x				[59]
24173237	miR-205-5p	CSS	PCa specific death	x	x				[59]
24173237	miR-205-5p	CFFS	histologically proven local recurrence or distant metastasis confirmed by CT or bone-scan	x	x				[59]
24173237	miR-205-5p	BPFS	PSA $\geq$ 0.2 ng/mL on two consecutive follow-up visits	x	x				[59]
19676045	miR-205-5p	BPFS	post-operative PSA value >0.1 lg/l confirmed by at least one subsequent rising value after the patients had reached an undetectable PSA level (detection limit <0.04 lg/l) after surgery						[10]
31122242	miR-20a-5p	BPFS	no definition						[39]
31122242	miR-20b-5p	BPFS	no definition						[39]
22341810	miR-21-5p	BPFS	Post-operative serum PSA 0.2 ng/ml or greater	x	x	x	x	surgical margin status, lymph node metastasis, capsular invasion	[61]
23353719	miR-21-5p	RFS	either an elevated PSA level ($\geq$ 0.2 ng/ml) after surgical treatment, clinical metastasis or disease specific death	x	x				[63]
25401698	miR-21-5p	BPFS	PSA>0.4 ng/ mL and rising in a minimum of two different blood samples postoperatively	x	x			non-apical positive surgical margin, apical positive surgical margin	[60]
27040772	miR-21-5p	PFS	no definition		x				[62]
23353719	miR-21-5p	RFS	either an elevated prostate-specific antigen level ($\geq$ 0.2 ng/ml) after surgical treatment, clinical metastasis or disease specific death	x	x				[63]

23353719	miR-21-5p	RFS	either an elevated prostate-specific antigen level ($\geq$ 0.2 ng/ml) after surgical treatment, clinical metastasis or disease specific death	x	x				[63]
25401698	miR-21-5p	BPFS	PSA $\geq$ 0.4 ng/mL and rising in a minimum of two different blood samples post-operatively	x	x			non-apical positive surgical margin, apical positive surgical margin	[60]
27824162	miR-210-3p	CFFS	symptomatic, locally advanced progression or metastasis to bone, visceral organs or lymph nodes verified by radiology	x	x	x	x	tumour size, perineural infiltration, lymphovascular infiltration, non-apical positive surgical margin	[64]
29917185	miR-212-3p	PS	no definition						[65]
30870834	miR-218-5p	bone MFS	no definition						[66]
30870834	miR-218-5p	OS	no definition						[66]
30870834	miR-218-5p	bone MFS	no definition						[66]
30870834	miR-218-5p	OS	no definition						[66]
19585579	miR-221-3p	CFFS	histologically proven local recurrence or distant metastasis confirmed by CT or bone scan	x	x	x	x		[67]
24760272	miR-221-3p	CRPC FS	castration resistance was evaluated through PSA recurrence, which was defined as two consecutive increasing PSA values of more than 1.0 ng/mL and differing by more than 0.2 ng/mL						[68]
31640261	miR-221-3p	BPFS	no definition					miR-30c-5p, miR-30d-5p, miR-31-5p, miR-141-3p, miR-148a-3p	[23]
22864280	miR-221-3p	BPFS	PSA $\geq$ 0.2 ng/ml at two consecutive follow-up visits						[29]
23353719	miR-221-3p	RFS	either an elevated prostate-specific antigen level ($\geq$ 0.2 ng/ml) after surgical treatment, clinical metastasis or disease specific death	x	x				[63]

23353719	miR-221-3p	RFS	either an elevated prostate-specific antigen level (≥ 0.2 ng/ml) after surgical treatment, clinical metastasis or disease specific death	x	x				[63]
23353719	miR-221-3p	RFS	either an elevated prostate-specific antigen level (≥ 0.2 ng/ml) after surgical treatment, clinical metastasis or disease specific death	x	x				[63]
26325107	miR-221-3p	CRPC FS	CRPC is defined as castrate serum testosterone <50 ng/dl or 1.7 nmol/l plus one of the following types of progression: biochemical progression, radiologic progression						[69]
19676045	miR-221-3p	BPFS	post-operative PSA value >0.1 lg/l confirmed by at least one subsequent rising value after the patients had reached an undetectable PSA level (detection limit <0.04 lg/l) after surgery	x	x	x	x	surgical margin status	[10]
24583788	miR-222-3p	BPFS	serum PSA of 0.2 ng/mL or greater (obtained 6 weeks – 3 months postoperatively), with a second confirmatory level of PSA greater than 0.2 ng/mL	x		x	x	body-mass index	[4]
26325107	miR-222-3p	CRPC FS	CRPC is defined as castrate serum testosterone <50 ng/dl or 1.7 nmol/l plus one of the following types of progression: biochemical progression, radiologic progression	x	x	x	x	lymph node met, distant met	[69]
23353719	miR-222-3p	RFS	either an elevated prostate-specific antigen level (≥ 0.2 ng/ml) after surgical treatment, clinical metastasis or disease specific death	x	x				[63]
23353719	miR-222-3p	RFS	either an elevated prostate-specific antigen level (≥ 0.2 ng/ml) after surgical treatment, clinical metastasis or disease specific death	x	x				[63]
23353719	miR-222-3p	RFS	either an elevated prostate-specific antigen level (≥ 0.2 ng/ml) after surgical treatment, clinical metastasis or disease specific death	x	x				[63]

19676045	miR-222-3p	BPFS	post-operative PSA value >0.1 lg/l confirmed by at least one subsequent rising value after the patients had reached an undetectable PSA level (detection limit <0.04 lg/l) after surgery	x	x	x	x	surgical margin status	[10]
24382668	miR-224-5p	BPFS	PSA $\geq$ 0.2 ng/mL on two occasions.	x	x	x	x		[70]
23136246	miR-224-5p	BPFS	the period between surgery and the persistent increase of serum PSA concentrations, evidenced by 2 consecutive PSA results $\geq$ 0.2 ng/mL	x	x	x			[71]
31876746	miR-23a-3p	OS	no definition		x		x	CRPC occurance time, survival time, outcome, body-mass index, tobacco smoking, family history of cancer, alcohol consumption	[73]
25714010	miR-23a-3p	PS	no definition	x	x			distant met	[72]
23074286	miR-23b-3p	OS	no definition						[74]
23074286	miR-23b-3p	RFS	no definition						[74]
30027097	miR-23b-3p	OS	no definition						[25]
27449037	miR-26a-3p	OS	no definition						[75]
30250598	miR-27a-3p	PS	no definition						[76]
25115396	miR-27b-3p	CRPC FS	CRPC is defined as castrate serum testosterone <50 ng/dl or 1.7 nmol/l plus one of the following types of progression: biochemical progression, radiologic progression	x	x	x	x	lymph node met, distant met	[77]
26990571	miR-301a-3p	BPFS	PSA increase $\geq$ 0.2 ng/ml on at least two occasions, at least 3 months apart	x	x	x	x	surgical margin status, lymph node status	[78]
24452717	miR-30c-5p	BPFS	the time interval between the initial surgery and the day of postoperative PSA 0.2 ng/ml or greater	x	x	x		surgical margin status	[79]
26499781	miR-30c-5p	PS	no definition						[54]

31640261	miR-30c-5p	BPFS	no definition					miR-30d-5p, miR-31-5p, miR-141-3p, miR-148a-3p, miR-221-3p	[23]
23231923	miR-30d-5p	BPFS	continuous elevation with a PSA level >0.2 ng/mL	x	x	x	x	SOCS1	[80]
28241827	miR-30d-5p	BPFS	no definition		x	x	x		[81]
31640261	miR-31-5p	BPFS	no definition					miR-30c-5p, miR-30d-5p, miR-141-3p, miR-148a-3p, miR-221-3p	[23]
19676045	miR-31-5p	BPFS	post-operative PSA value >0.1 lg/l confirmed by at least one subsequent rising value after the patients had reached an undetectable PSA level (detection limit <0.04 lg/l) after surgery	x	x	x	x	surgical margin status	[10]
25760964	miR-320e	BPFS	recurrence after salvage radiation at least twice consecutively following the nadir	x				lymph node status	[7]
30243091	miR-326	OS	the period from radical prostatectomy to death or at the end of the last follow-up						[82]
30243091	miR-326	BPFS	two continuous values of serum PSA level $\geq$ 0.2 ng/ml after radical prostatectomy						[82]
23456549	miR-335-5p	OS	no definition						[83]
23456549	miR-335-5p	MFS	no definition						[83]
23456549	miR-335-5p	BPFS	no definition						[83]
26907180	miR-338-3p	BPFS	time from radical prostatectomy to PSA recurrence						[84]
23147995	miR-34b-3p	BPFS	the first postoperative PSA value greater than 0.1 ng/mL, confirmed by at least 1 undetectable PSA level (detection limit <0.04 ng/mL) after surgery						[85]
28143614	miR-34b/c	DFS	the date of the radical prostatectomy or other curative treatment to the date of biochemical relapse, date of last follow-up, or death if relapse-free		x				[14]

28143614	miR-34b/c	DSS	the time elapsed since diagnosis until death or the last follow-up		x				[14]
21351256	miR-34c-5p	PS	survival time was measured from the time of TURP						[86]
24817628	miR-3607-5p	PS	no definition						[87]
28498363	miR-3622a-5p	OS	no definition						[88]
27611943	miR-3622b-5p	BPFS	no definition						[89]
27611943	miR-3622b-5p	OS	no definition						[89]
30338790	miR-373-3p	OS	no definition						[65]
24191917	miR-374b-5p	BPFS	no definition	x	x	x			[91]
25129854	miR-375	OS	no definition						[15]
19676045	MiR-375	BPFS	post-operative PSA value >0.1 lg/l confirmed by at least one subsequent rising value after the patients had reached an undetectable PSA level (detection limit <0.04 lg/l) after surgery	x	x	x	x	surgical margin status	[10]
25153390	miR-378-3p	DFS	no definition	x	x	x	x	digital rectal examination	[92]
25153390	miR-378-3p	DFS	no definition	x	x	x	x	digital rectal examination	[92]
25324143	miR-379-5p	DFS	no definition						[93]
24963047	miR-409-3p	DFS	no definition						[94]
29969630	miR-410-3p	OS	no definition						[95]
21149617	miR-423-3p	CSS	no definition					KIF3C SNP, PALLD SNP,GABRA1 SNP, SYT6 SNP, ZDHHC7 SNP	[96]
31337863	miR-424-3p	CFFS	clinically palpable tumor recurrence or metastasis verified by radiology	x				vascular infiltration	[97]
30874288	MiR-4288	OS	no definition						[98]
29633185	miR-4319	OS	no definition						[99]
25416653	miR-449b-5p	BPFS	two consecutive measurements of PSA >0.2 ng/mL	x	x	x	x	surgical margin status	[100]
25760964	miR-4516	BPFS	The recurrence after salvage radiation at least twice consecutively following the nadir	x				lymph node status	[7]

27070713	miR-452-5p	CRPC FS	CRPC described as castrate serum levels of testosterone (testosterone <50 ng/dl); Three consecutive rises of prostate-specific antigen (PSA), 1 wk apart, resulting in two 50% increases over the nadir with PSA >2.0 ng/ml; Antiandrogen withdrawal for at least 4 wk for flutamide and for at least 6 wk for bicalutamide; PSA progression, despite consecutive hormonal manipulations; Progression of osseous lesions			[101]
27634912	miR-4534	OS	no definition			[102]
31111062	miR-455-5p	RFS	no definition			[103]
28125091	miR-466	RFS	no definition			[104]
24223753	miR-4723-5p	PS	no definition			[105]
28631332	miR-500a-5p	OS	no definition			[106]
27267060	miR-503-5p	PS	no definition			[107]
30365141	miR-505-3p	bone MFS	no definition			[108]
30365141	miR-505-3p	OS	no definition			[108]
25760964	miR-508-3p	BPFS	The recurrence after salvage radiation at least twice consecutively following the nadir	x	lymph node status	[7]
30685303	miR-515-5p	OS	no definition			[109]
25234358	miR-548c-3p	RFS	no definition			[110]
25760964	miR-563	BPFS	The recurrence after salvage radiation at least twice consecutively following the nadir	x	lymph node status	[7]
26451614	miR-573	MFS	no definition			[111]
30852380	miR-582-3p	bone MFS	no definition			[112]
30852380	miR-582-3p	OS	no definition			[112]
30852380	miR-582-5p	bone MFS	no definition			[112]
30852380	miR-582-5p	OS	no definition			[112]
25760964	miR-598	BPFS	The recurrence after salvage radiation at least twice consecutively following the nadir	x	lymph node status	[7]
25760964	miR-601	BPFS	The recurrence after salvage radiation at least twice consecutively following the nadir			[7]

24740842	miR-605	BPFS	the period of time elapsed between the date of RP and two consecutive PSA values of at least 0.3 ng/ml, one PSA value of at least 0.3 ng/ml followed by androgen-deprivation therapy or radiation therapy, and a single last-recorded PSA value of at least 0.3 ng/ml after RP	x	x	x	x	surgical margin status	[113]
31539518	miR-615-3p	CSS	PCa specific death					Capra-S score	[114]
31539518	miR-615-3p	BPFS	PSA $\geq$ 0.2 ng/mL					Capra-S score	[114]
31539518	miR-615-3p	BPFS	PSA $\geq$ 0.2 ng/mL					Capra-S score	[114]
31539518	miR-615-3p	BPFS	PSA $\geq$ 0.2 ng/mL					Capra-S score	[114]
31539518	miR-615-3p	BPFS	PSA $\geq$ 0.2 ng/mL					Capra-S score	[114]
25760964	miR-626	BPFS	The recurrence after salvage radiation at least twice consecutively following the nadir	x				lymph node status	[7]
25760964	miR-628-3p	BPFS	The recurrence after salvage radiation at least twice consecutively following the nadir	x				lymph node status	[7]
29721191	miR-652-3p	BPFS	PSA increase of at least 0.2 ng/mL on at least two separate consecutive measurements that are at least 3 months apart	x	x	x	x	margin status	[115]
29721191	miR-652-3p	MFS	lesions within the bone identified on radionuclide bone scan and lymphadenopathy or visceral lesions identified by computed tomography imaging of the abdomen, pelvis and chest.	x	x	x			[115]
24243035	miR-663a	PFS	histologically proven local recurrence or distant metastasis confirmed by CT or bone scan	x	x	x	x		[116]
24760272	miR-7-5p	CRPC FS	castration resistance was evaluated through prostate-specific antigen (PSA) recurrence, which was defined as two consecutive increasing PSA values of more than 1.0 ng/mL and differing by more than 0.2 ng/mL						[68]
22552290	miR-708-5p	PS	no definition						[117]

28107193	miR-744-5p	BPFS	no definition	x	x	x	x	lymph node invasion, surgical margin status, extracapsular extension, seminal vesicle invasion	[118]
30582208	miR-93-5p	RFS	no definition						[119]
30582208	miR-93-5p	MFS	no definition						[119]
30582208	miR-93-5p	OS	no definition						[119]
30027097	miR-95-3p	OS	no definition						[25]
23951320	miR-96-5p	OS	no definition						[120]
19676045	miR-96-5p	BPFS	post-operative PSA value >0.1 lg/l confirmed by at least one subsequent rising value after the patients had reached an undetectable PSA level (detection limit <0.04 lg/l) after surgery	x	x	x	x	surgical margin status	[10]
22864280	miR-96-5p	BPFS	PSA $\geq$ 0.2 ng ml at two consecutive follow-up visits						[29]

Table S2: **A table of endpoint definitions and adjusted variables included in multivariate Cox PH analyses for the studies in the systematic review; accompanying table for Supplementary Table S1.** "x" represents the variable was included in the multi-variate analysis. Abbreviations: BMI=Body mass index; CRPC=Castration Resistant Prostate Cancer; DRE=Digital Rectal Examination; PSA=Prostate specific antigen; SNP=Single-nucleotide polymorphism, TNM=Tumour, node, metastasis. For the full form of the abbreviated endpoints, refer to Table 2.

Prognostic miR	Prognostic test			Sample size	Sample type	PMID	Ref
	**test: endpoint**	**HR (95% CI)**	**p**				
miR-185-5p, miR-221-3p, miR-326	multivariate: BPFS	1.36 (1.03-1.79)	0.031	126 (cohort 1)	tissue	27120795	[121]
	multivariate: BPFS	1.28 (1.00-1.64)	0.048	110 (cohort 2)	tissue		
	multivariate: BPFS	1.91 (1.26-2.91)	0.012	99 (cohort 3)	tissue		
let-7a-5p, miR-125-5p,miR-151a-5p	multivariate: BPFS	0.61 (0.41-0.90)	0.013	122 (cohort 1)	urine	28753866	[122]
	multivariate: BPFS	0.47 (0.28-0.77)	0.003	133 (cohort 2)	urine		
miR-10b-5p, miR-133a, miR-23a-3p, miR-374b-5p	multivariate: BPFS	2.43 (1.45-4.07)	0.008	123 (cohort PCA123)	tissue	30010760	[123]
	multivariate: BPFS	1.44 (1.04-2.00)	0.029	352 (cohort PCA352)	tissue		
	multivariate: BPFS	1.89 (1.08-3.32)	0.027	476 (cohort PCA476)	tissue		
	multivariate: CSS	2.43 (1.45-4.07)	0.021	352 (cohort PCA476)	tissue		
miR-145-5p, miR-183-5p, miR-96-5p, miR-221-5p	univariate: PS	6.50 (n/s)	0.001	49 (cohort 1)	tissue	23184647	[124]
	univariate: PS	6.20 (n/s)	0.001	71 (cohort 2)	tissue		
	univariate: BPFS	2.70 (n/s)	0.007	71 (cohort 2)	tissue		
miR-139-5p, miR-223, miR-301a-3p, miR-454-3p, miR-652-3p	multivariate: BPFS	2.60 (1.80-3.60)	<0.001	491	tissue	26516365	[125]
	multivariate: MFS	4.30 (1.60-11.10)	0.002	491	tissue		
miR-132-3p, miR-200a-3p, miR-200b-3p, miR-200c-3p, miR-375, miR-429	multivariate: OS	3.20 (1.81-5.91)	<0.001	97 (cohort 1)	plasma	28278515	[126]
	multivariate: OS	3.30 (1.64-6.63)	0.001	85 (cohort 2)	plasma		
miR-17-5p, miR-18a-5p, miR-19a-3p, miR-19b-3p, miR-20a-5p, miR-92a-3p	KM: BPFS	-	<0.050	29	tissue	29163712	[127]
let-7a-5p, miR-106a-5p, miR-107, miR-130b-3p, miR-26b-5p, miR-223, miR-451a	KM: BPFS	-	0.031	100	serum	25874774	[128]

Table S3: **A table of signature miRs of prognostic importance in PCa identified in the systematic review.** KM, univariate and multivariate tests stand for Kaplan-Meier test, and univariate and multivariate Cox PH regressions respectively. Refer to Supplementary Table S4 for endpoint definitions and adjusted variables included in the multivariate analyses. Refer to Table 2 for the full form of the abbreviated endpoints. n/s represents not-specified.

PMID	Endpoint	Endpoint definition	Variables in multivariate Cox PH analysis					Ref
			Gleason	T stage	PSA	age	others	
27120795	BPFS	PSA cut-off $\geq$ 0.2 ng/ml	x	x	x	x	SMS	[121]
28753866	BPFS	PSA $\geq$ 0.2 ng/ml	x		x			[122]
30010760	BPFS	a postoperative PSA test $\geq$ 0.2 ng/ml					Capra-s	[123]
23184647	PS	patient/ percent survival						[124]
26516365	BPFS	a PSA increase of at least 0.2 ng/mL on at least two separate consecutive measurements that are at least 3 months apart	x	x	x			[125]
28278515	OS	the time from initiation of the first cycle of docetaxel to the time of death or last follow-up			x		haemoglobin, alkaline phosphatase	[126]
29163712	BPFS	no def						[127]
25874774	BPFS	no def						[128]

Table S4: **A table of endpoint definitions and adjusted variables for the studies in the systematic review; accompanying table for Supplementary Table S3**. "x" represents the variable was included in the multi-variate analysis. Abbreviations: BMI=Body mass index; CRPC=Castration Resistant Prostate Cancer; DRE=Digital Rectal Examination; PSA=Prostate specific antigen; SNP=Single-nucleotide polymorphism, TNM=Tumour, node, metastasis. For the full form of the abbreviated endpoints, refer to Table 2.

Characteristics		TCGA-PRAD	GSE88958	GSE46738	GSE26247	GSE26245	GSE21036
**number of samples**	**total**	433	30	50	40	63	99
	**BCR**	63	19	12	13	25	19
	**non-BCR**	370	11	38	27	38	80
**follow-up time (months)**	**median**	28.73	69.83	25.50	49.50	49.97	46.39
	**range**	0.00-165.17	1.38-118.16	1.35-120.20	1.00-164.00	0.99-163.99	1.35-128.42
**PSA at diagnosis (ng/mL)**	**$<$10**	364	12	36	14	41	80
	**10-20**	3	4	13	17	18	12
	**$>$ 20**	26	3	0	4	2	6
	**NA**	40	11	1	5	2	1
**age at diagnosis**	**median**	61	63	66	64	63	57
	**range**	41-78	40-75	49-77	49-82	45-79	37-83
**tumour stage**	**version**	pathological	NA	pathological	pathological	pathological	clinical
	**T1**	2	NA	0	0	12	0
	**T2**	168	NA	22	27	42	69
	**T3**	251	NA	28	10	6	25
	**T4**	9	NA	0	3	3	5
	**$\geq$ 6**	44	8	15	12	18	32
	**7**	212	17	13	18	39	54
	**$\leq$ 8**	177	5	22	10	6	12

Table S5: **Sample characteristics of the datasets included in the data reanalysis.** Abbreviations: BCR=Biochemical recurrence; NA=Not available; PSA=Prostate specific antigen.

Supplementary Figures

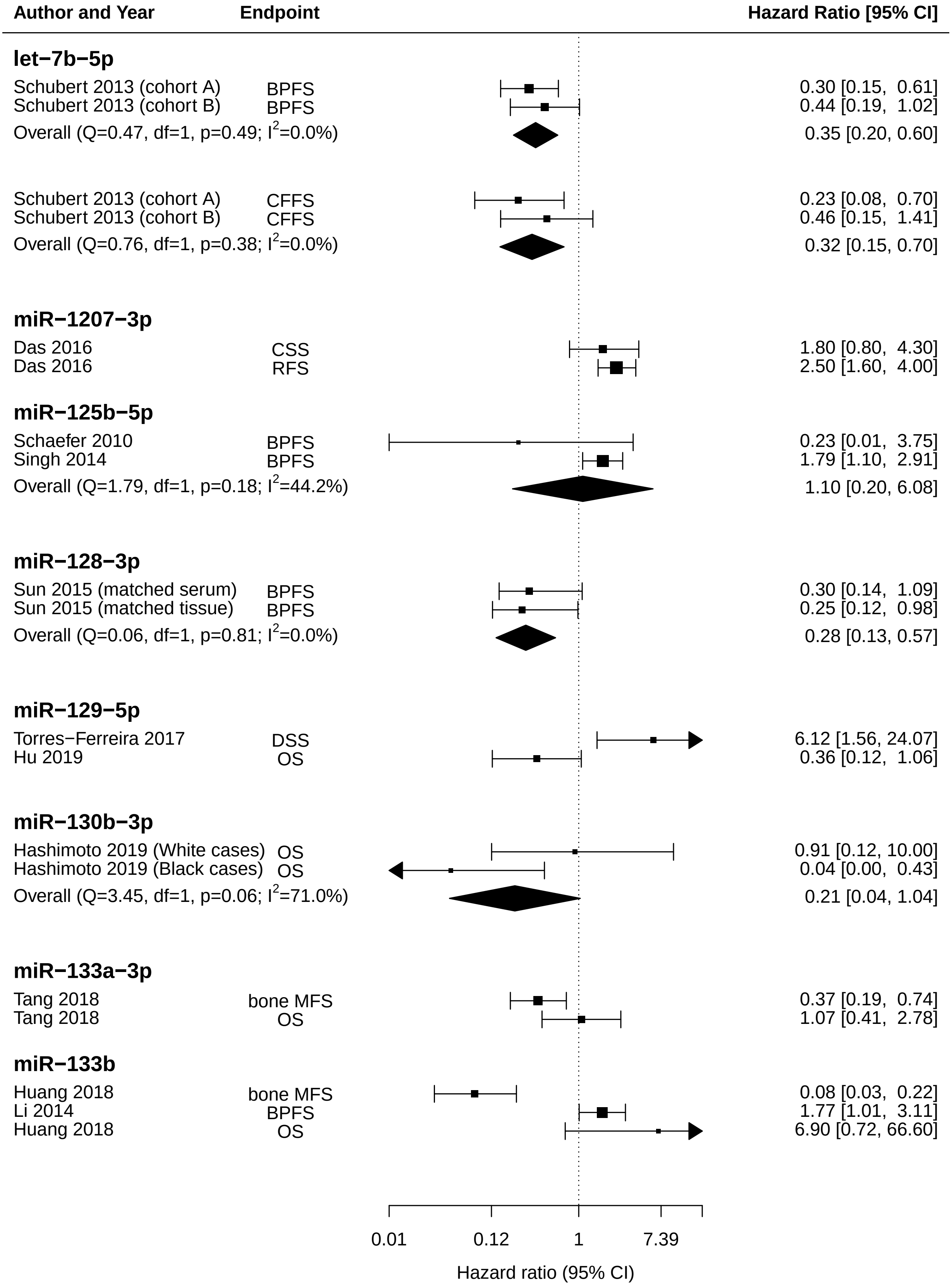

Figure S1: Figure continued in next page.

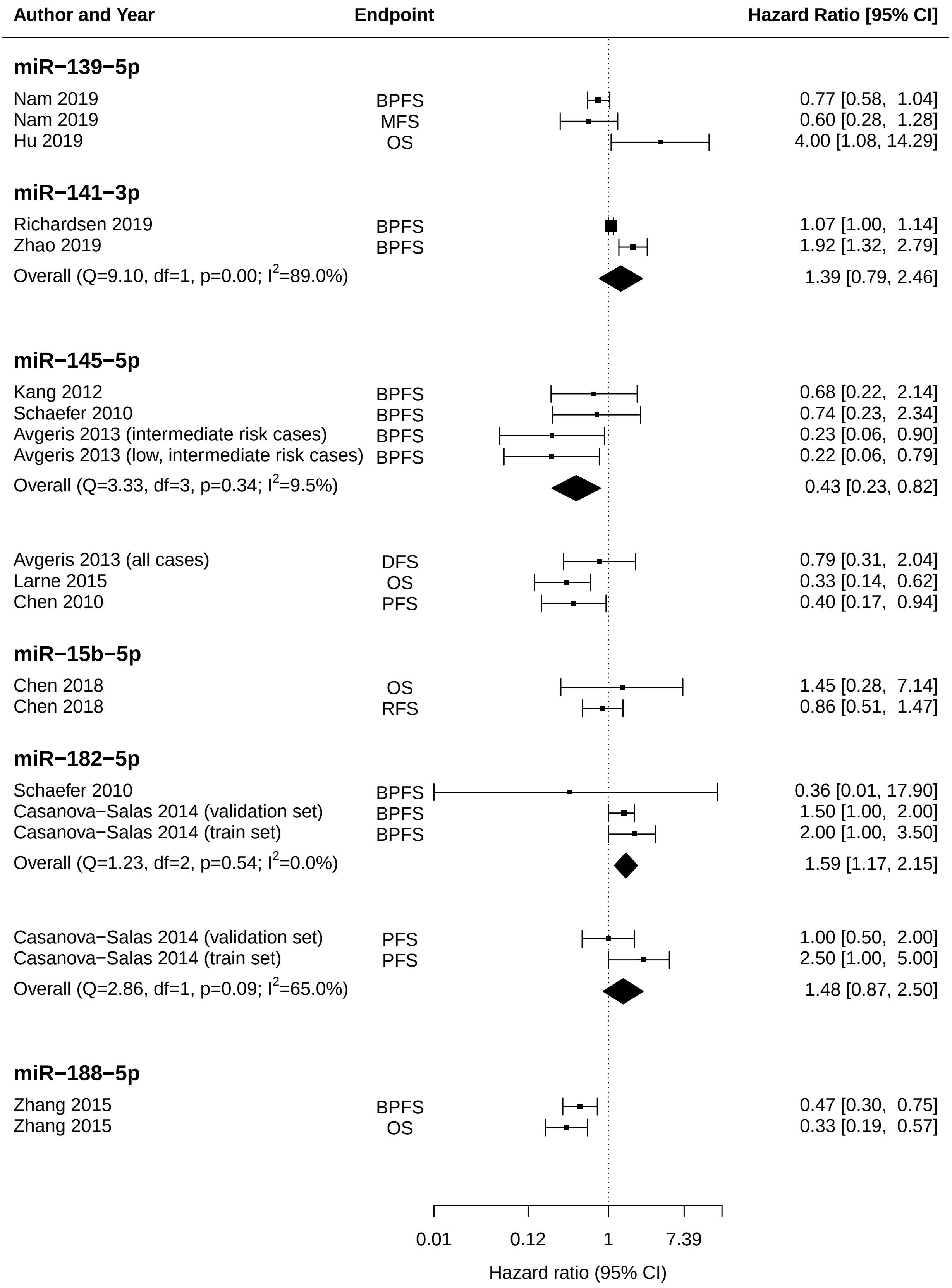

Figure S1: Figure continued in next page.

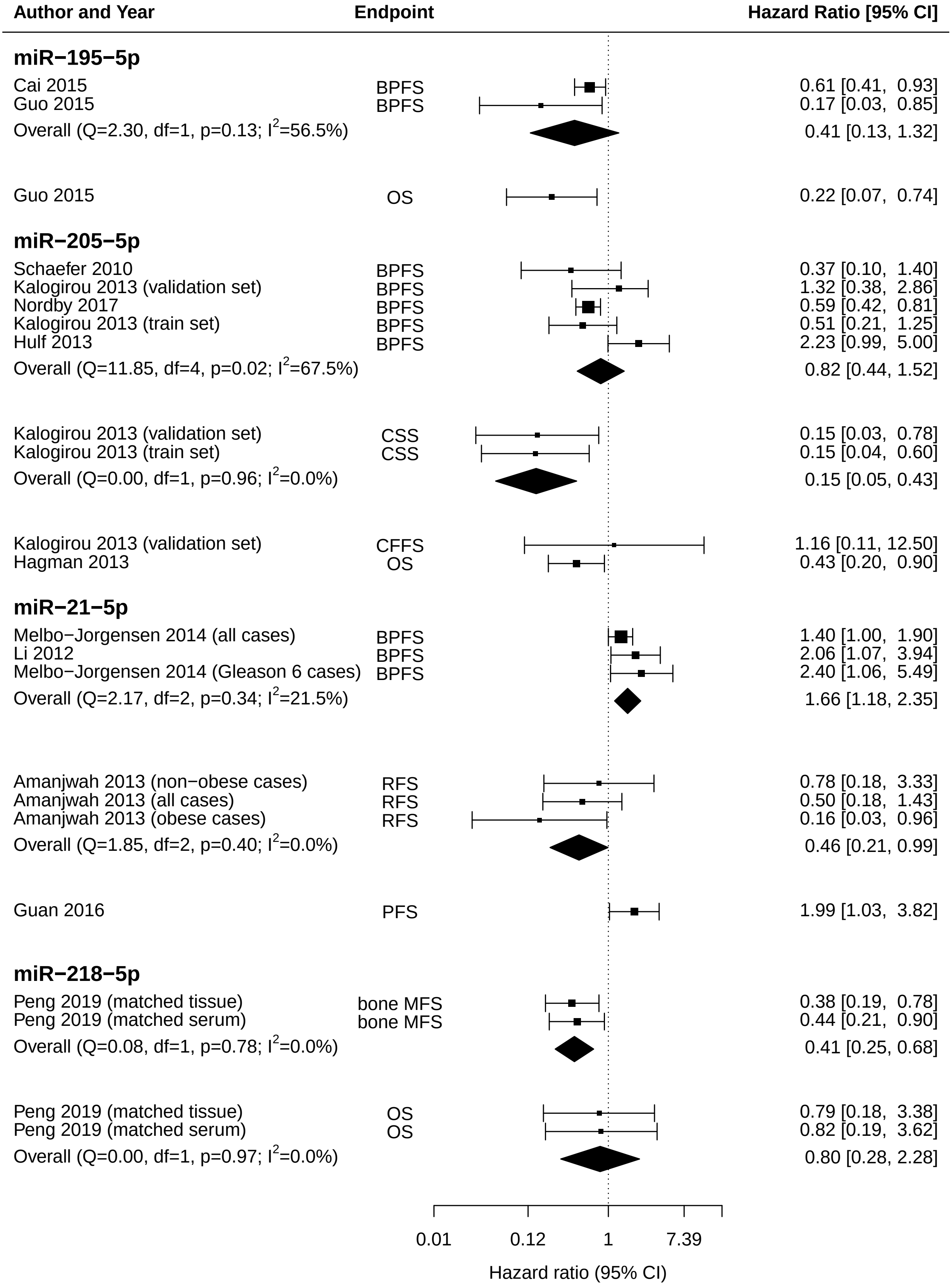

Figure S1: Figure continued in next page.

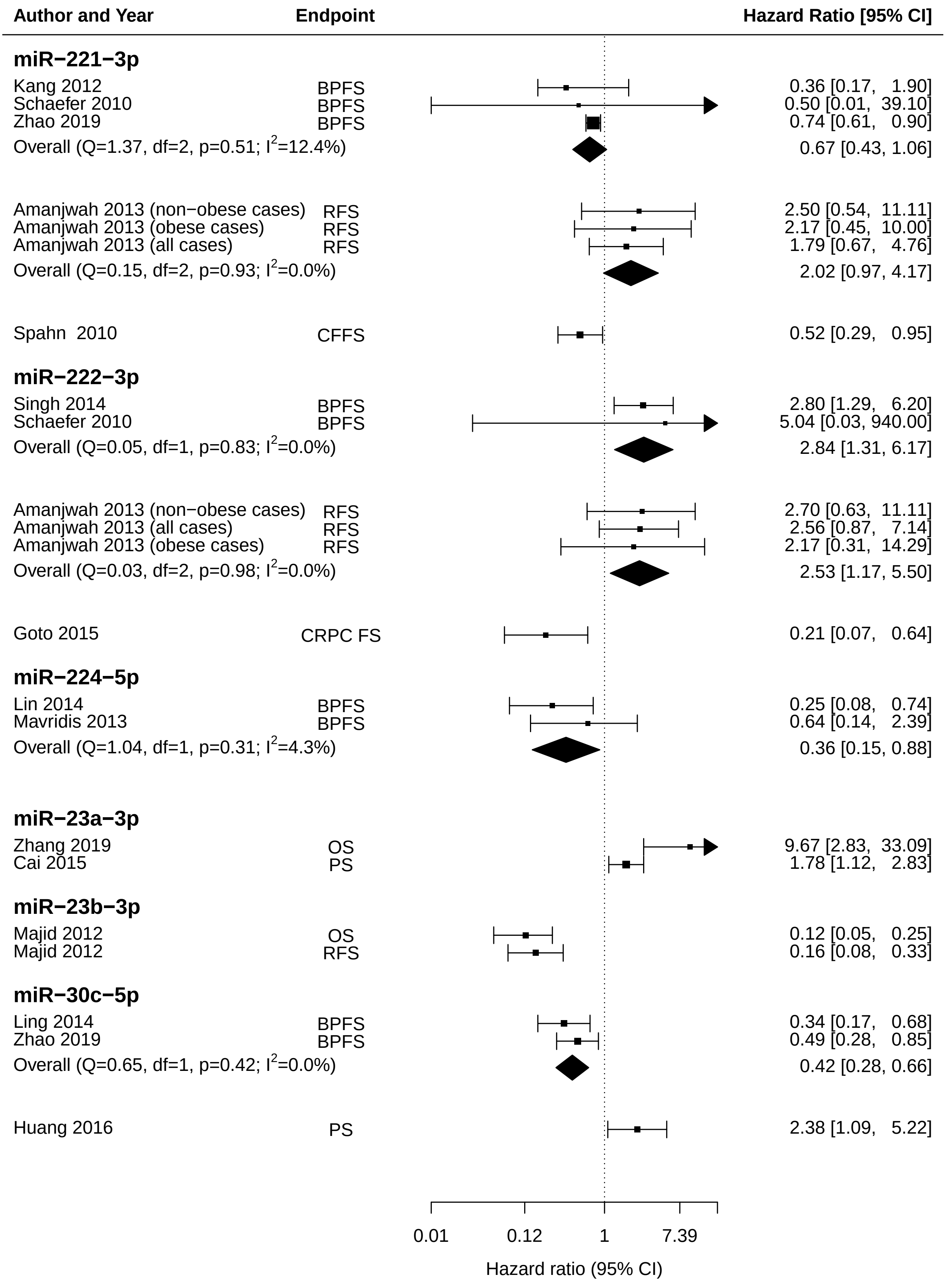

Figure S1: Figure continued in next page.

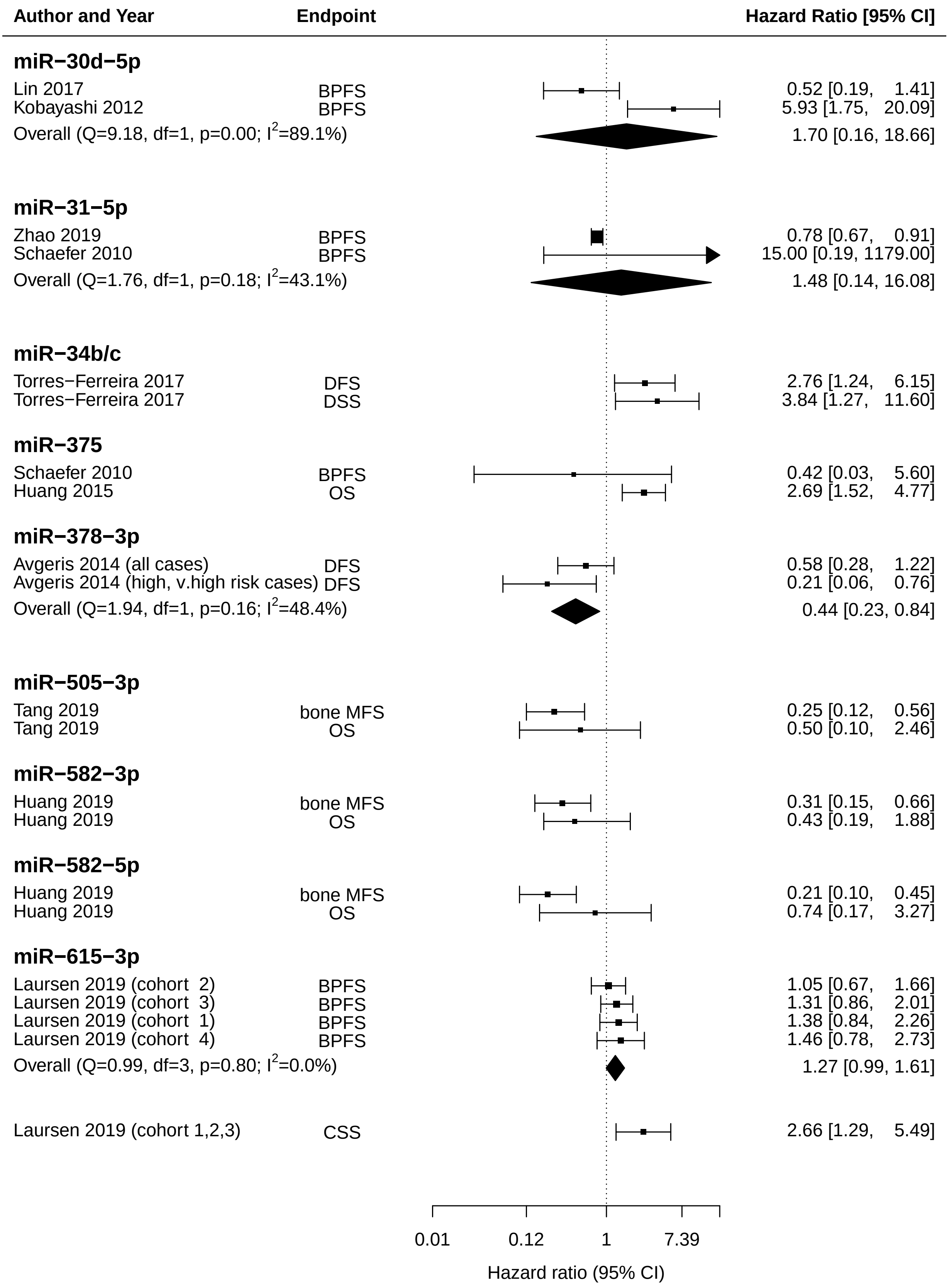

Figure S1: Figure continued in next page.

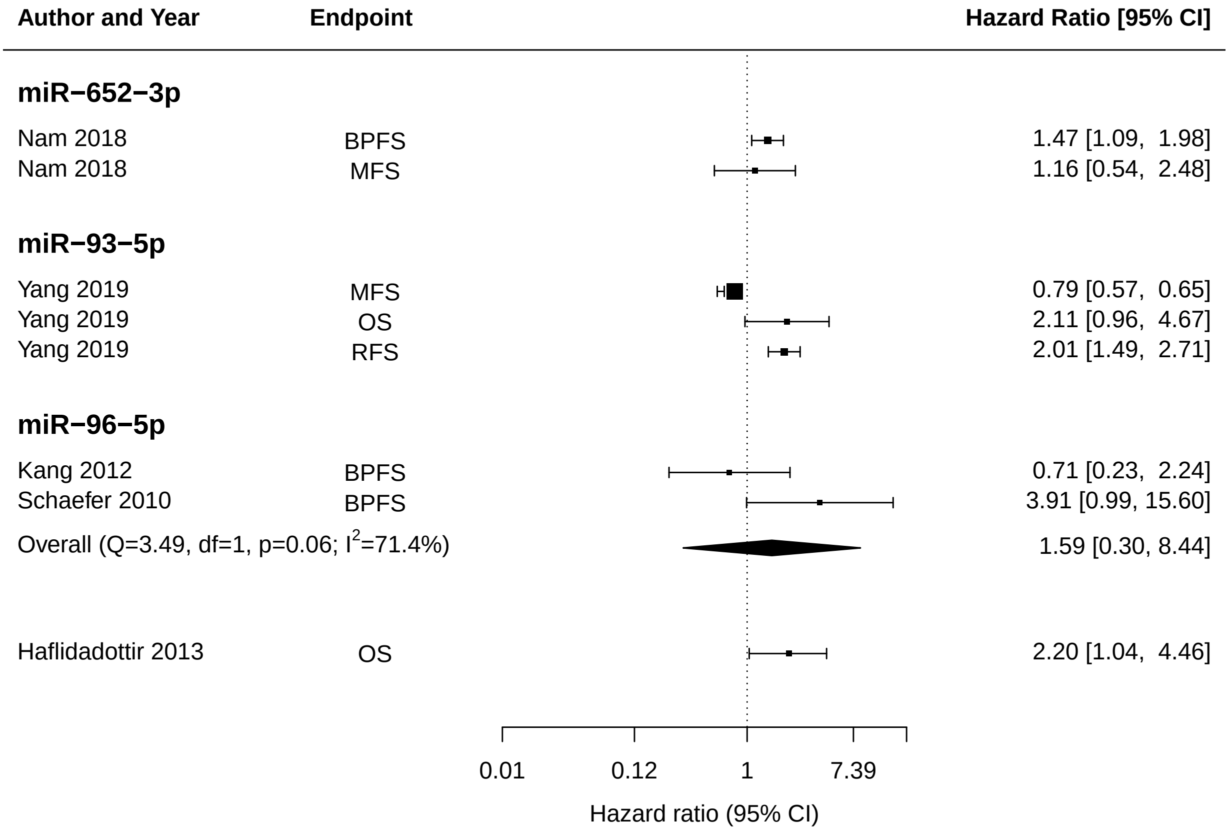

Figure S1: Forest plots for the miRs with multiple entries. For miRs that had multiple entries and the same progression endpoints, a random-effects model was performed to get an overall effect estimate (pooled hazard ratio). Hazard ratio was standardised by setting the "low" miR expression as the reference group in all the studies.

Author and Year	miRNAs	endpoint	Hazard Ratio [95% CI]
Schubert 2013	let−7c	CFFS	0.53 [0.19, 1.48]
Hudson 2012	miR−1−3p	DFS	0.29 [0.10, 0.90]
Leite 2011	miR−100−5p	BPFS	3.65 [1.38, 9.62]
Singh 2014	miR−103a−3p	BPFS	0.41 [0.21, 0.79]
Hudson 2013	miR−106b−5p	DFS	2.70 [1.10, 7.30]
Fendler 2011	miR−10b−5p	BPFS	2.15 [1.02, 4.51]
Bell 2015	miR−1193	BPFS	5.00 [1.60, 15.60]
Wang 2019	miR−1231	OS	2.17 [1.03, 4.56]
Sun 2013	miR−126−3p	BPFS	0.27 [0.15, 1.01]
Huang 2015	miR−1290	OS	0.12 [0.04, 0.42]
Zedan 2017	miR−143−5p	RFS	0.32 [0.12, 0.81]
Selth 2013	miR−146−3p	BPFS	1.16 [1.03, 1.31]
Chen 2014	miR−146a−5p	BPFS	0.83 [0.30, 2.32]
Zhao 2019	miR−148a−3p	BPFS	0.60 [0.44, 0.81]
Schaefer 2010	miR−149−5p	BPFS	0.68 [0.22, 2.15]
Ramalho−Carvalho 2018	miR−152−3p	DFS	0.23 [0.07, 0.72]
Bi 2019	miR−153	OS	2.48 [1.58, 10.73]
Schaefer 2010	miR−16−5p	BPFS	48.40 [1.26, 1858.00]
Schaefer 2010	miR−181b−5p	BPFS	1.97 [0.21, 18.70]
Schaefer 2010	miR−182−3p	BPFS	0.43 [0.01, 17.80]
Schaefer 2010	miR−183−3p	BPFS	27.40 [0.46, 1622.00]
Schaefer 2010	miR−184	BPFS	3.48 [0.16, 73.90]
Liu 2019	miR−191−5p	OS	2.31 [1.67, 9.01]
Selth 2013	miR−194−5p	BPFS	1.08 [0.91, 1.28]
Huang 2016	miR−203a−3p	PS	2.52 [1.14, 5.55]
Wa 2019	miR−204−5p	bone MFS	0.25 [0.14, 0.48]
Andersen 2016	miR−210−3p	CFFS	2.76 [1.25, 6.09]
Goto 2014	miR−27b−3p	CRPC FS	0.26 [0.07, 0.94]
Nam 2016	miR−301a−3p	BPFS	1.42 [1.06, 1.90]
Bell 2015	miR−320e	BPFS	3.20 [1.10, 9.60]
Bakkar 2016	miR−338−3p	BPFS	0.78 [0.54, 1.14]
Majid 2013	miR−34b−3p	BPFS	0.30 [0.11, 0.77]
He 2013	miR−374b−5p	BPFS	0.38 [0.17, 0.85]
Bao 2011	miR−423−3p	CSS	0.64 [0.40, 1.01]
Richardsen 2019	miR−424−3p	CFFS	0.44 [0.22, 0.87]
Mortensen 2014	miR−449b−5p	BPFS	1.90 [1.25, 2.85]
Bell 2015	miR−4516	BPFS	3.60 [1.30, 10.00]
Nip 2016	miR−4534	OS	6.00 [3.00, 17.00]
Bell 2015	miR−508−3p	BPFS	3.00 [1.10, 8.00]
Bell 2015	miR−563	BPFS	0.30 [0.10, 0.80]
Bell 2015	miR−598	BPFS	0.30 [0.10, 0.90]
Bell 2015	miR−601	BPFS	4.60 [1.60, 12.70]
Huang 2014	miR−605	BPFS	0.51 [0.30, 0.86]
Bell 2015	miR−626	BPFS	0.30 [0.10, 0.90]
Bell 2015	miR−628−3p	BPFS	6.60 [1.90, 23.50]
Jiao 2014	miR−663a	PFS	2.92 [1.98, 4.32]
Saini 2012	miR−708−5p	PS	0.17 [0.06, 0.45]
Guan 2017	miR−744−5p	BPFS	8.27 [1.85, 37.06]

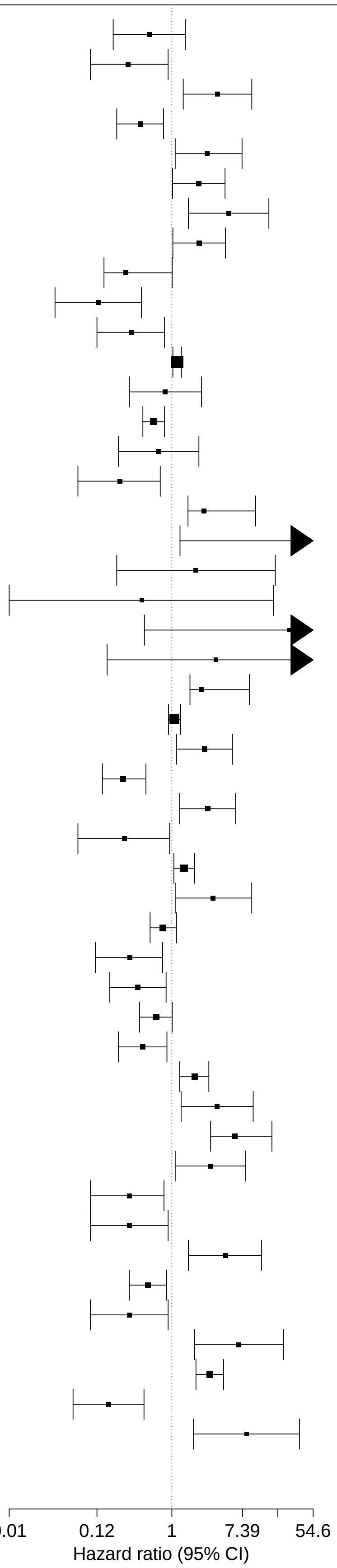

Figure S2: Forest plot for miRs with single entries in the systematic review. Hazard ratio was standardised by setting the “low” miR expression as the reference group in all the studies.

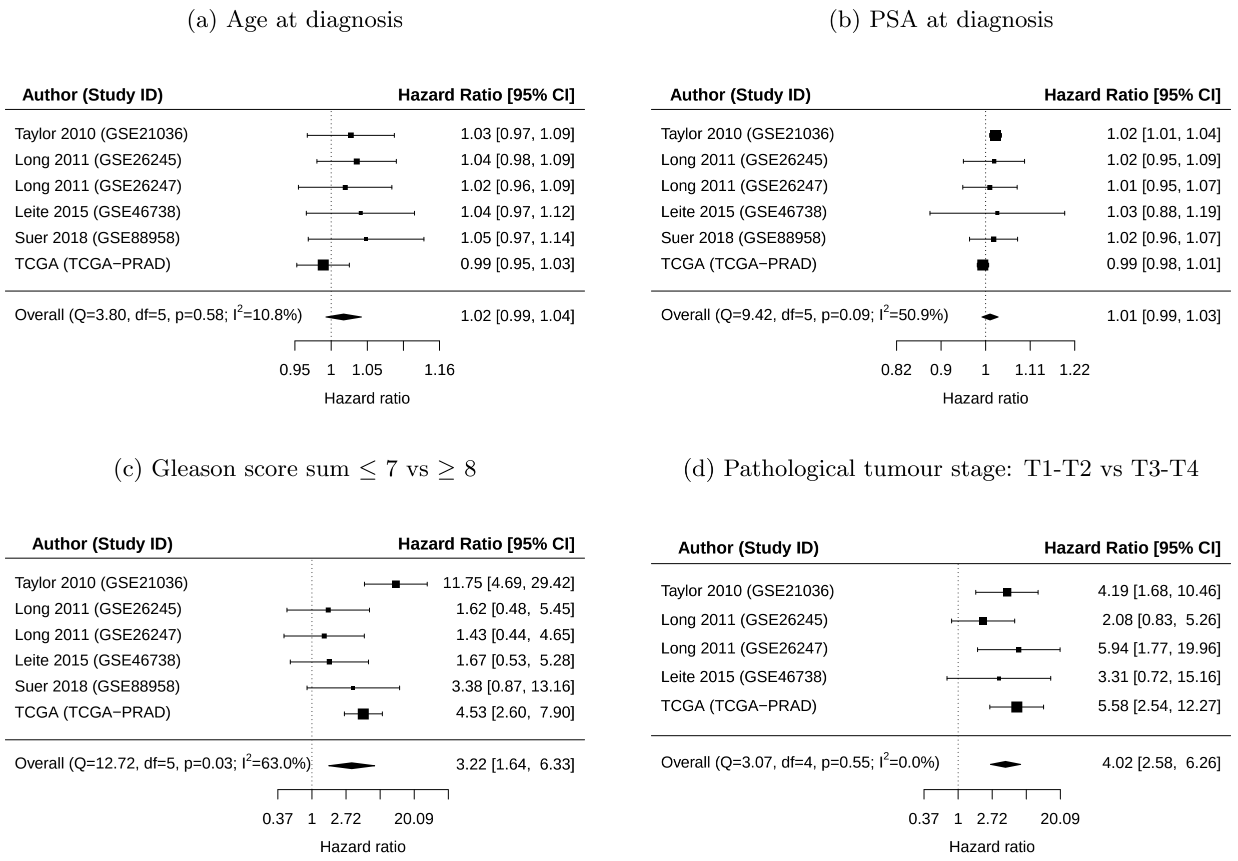

Figure S3: Association of clinical characteristics with biochemical recurrence. Whilst age and PSA show a non-significant association with biochemical recurrence, there were significant overall associations of higher Gleason score and tumour stage with biochemical recurrence. "Overall" refers to the overall effect estimate, i.e. pooled hazard ratio. The "Q", "df" and "p" values refer to the statistics for Q-test for heterogeneity and the "I^2" value refers to the outcome of I^2 test for heterogeneity. Abbreviations: PSA=Prostate specific antigen.

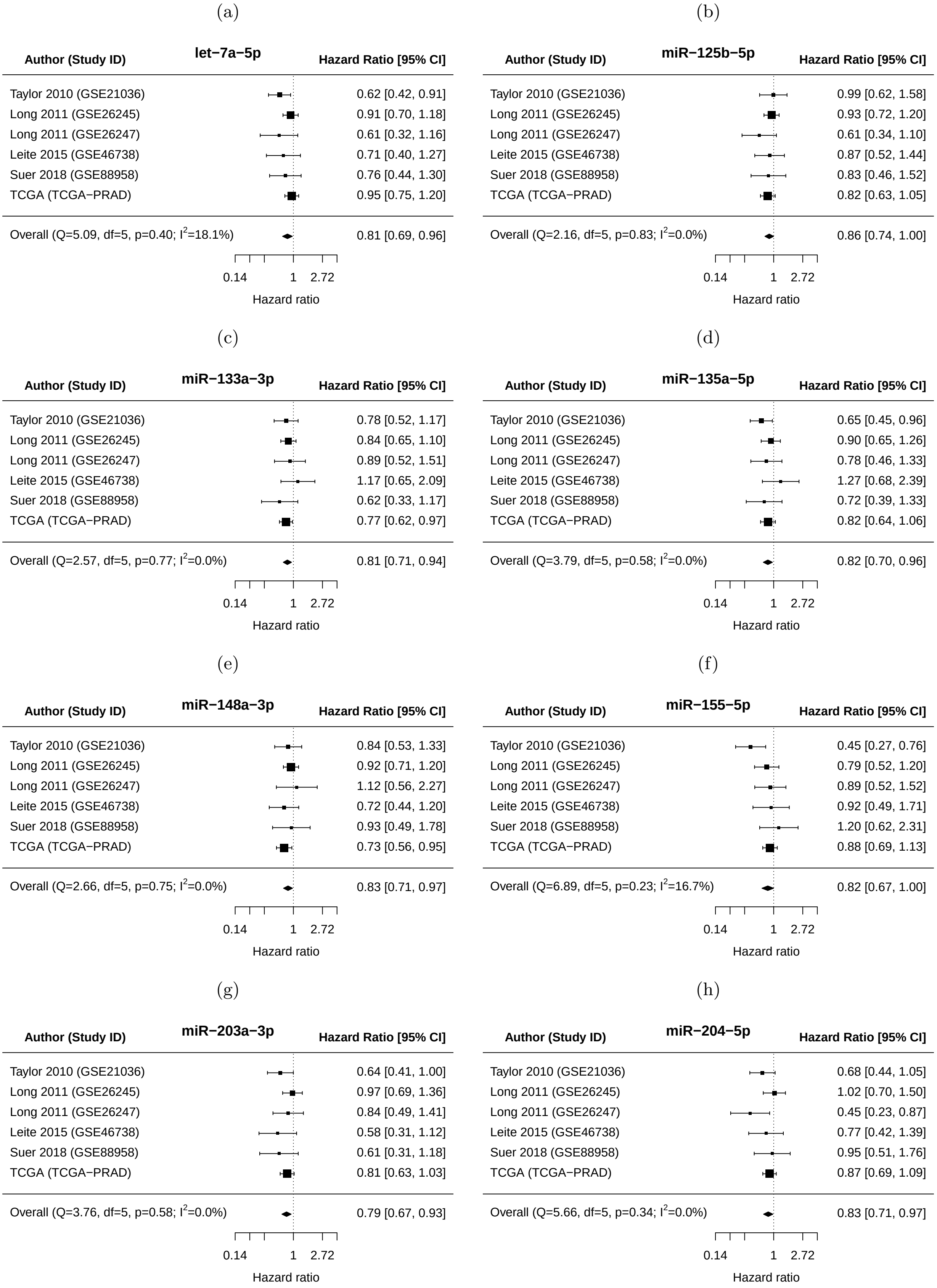

Figure S4: Figure continued in next page.

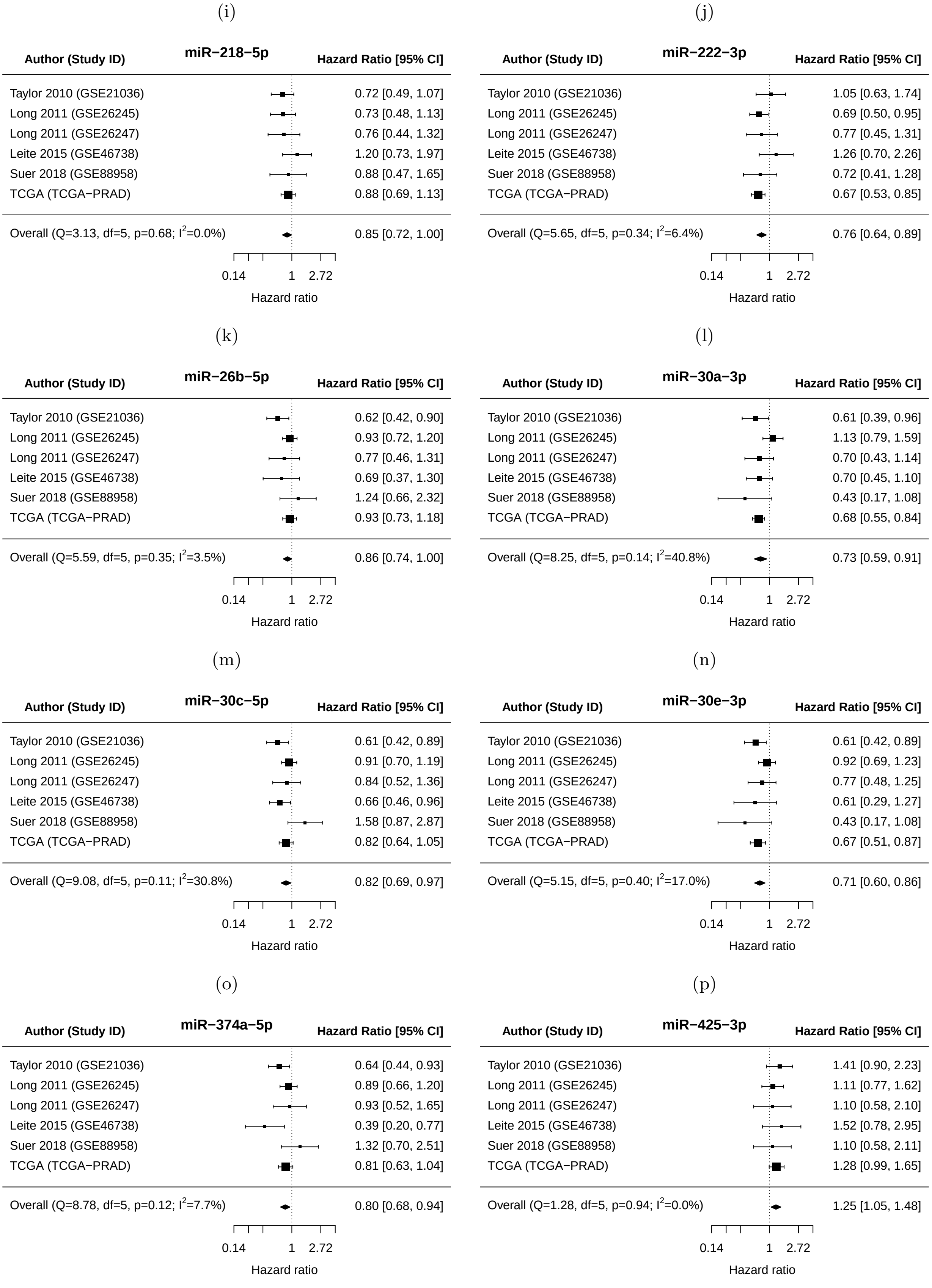

Figure S4: Figure continued in next page.

(q)

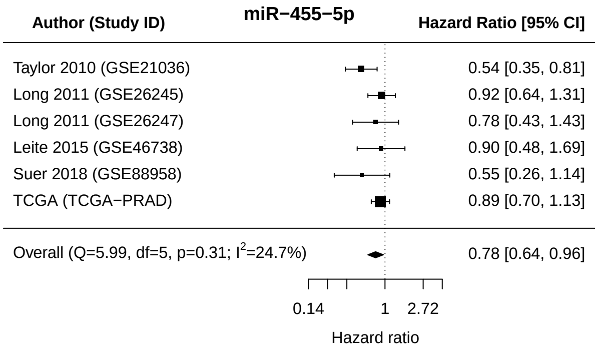

(r)

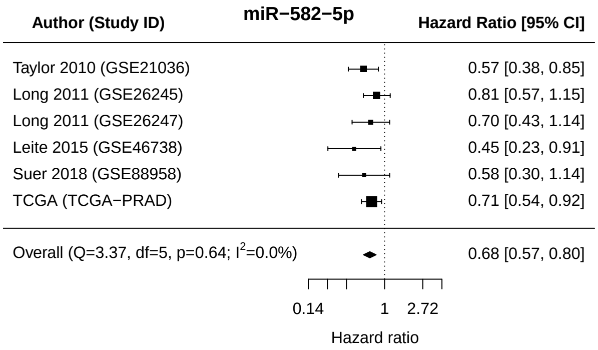

Figure S4: Association of miR expression with biochemical recurrence: an univariate analysis. The overall associations of these 18 miRs were significantly associated with biochemical recurrence. The expression of all but miR-425-3p associated negatively with recurrence. "Overall" refers to the overall effect estimate, i.e. pooled hazard ratio. The "Q", "df" and "p" values refer to the statistics for Q-test for heterogeneity and the "I^2" value refers to the outcome of I^2 test for heterogeneity.

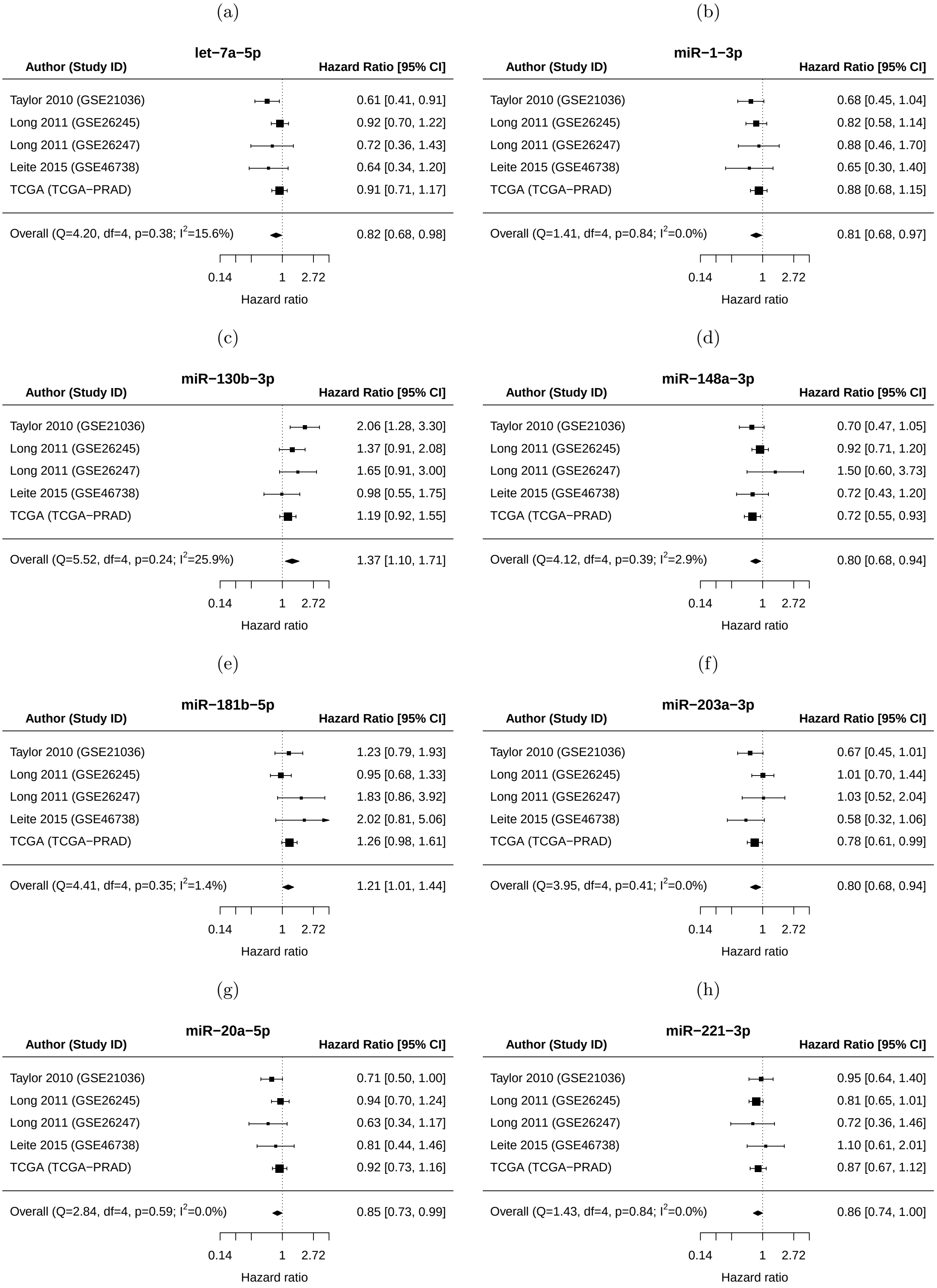

Figure S5: Figure continued in next page.

(i)

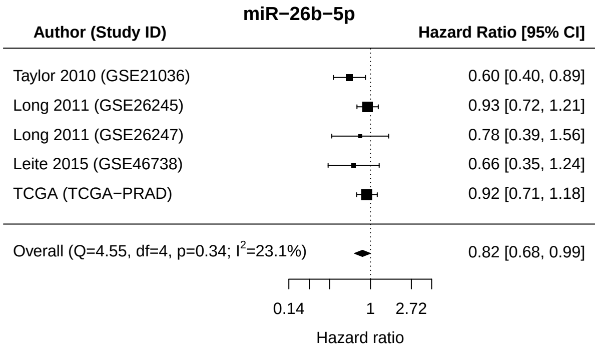

(j)

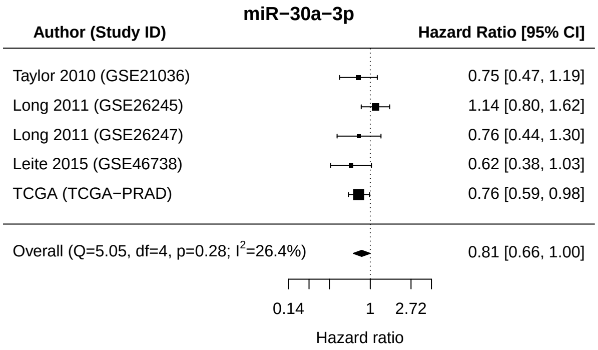

(k)

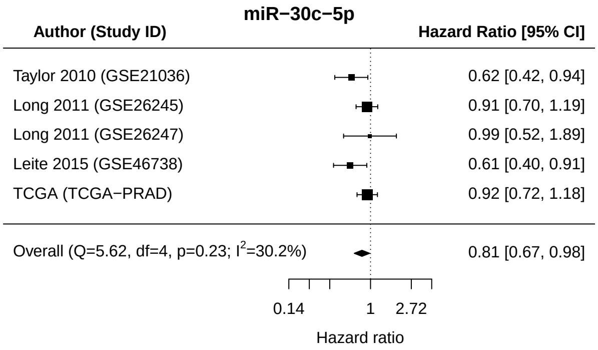

(l)

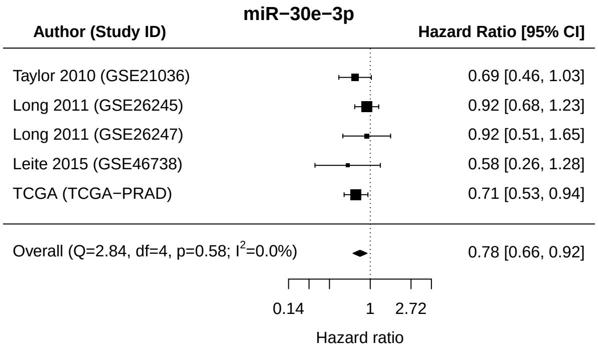

(m)

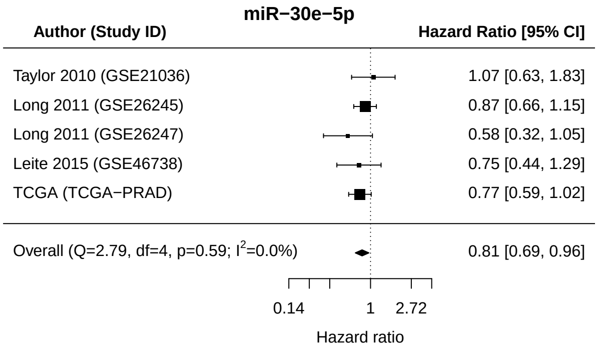

(n)

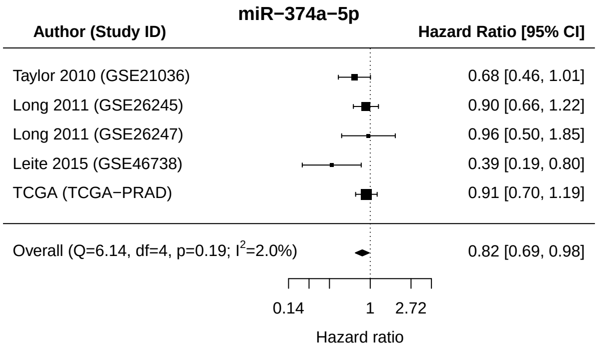

(o)

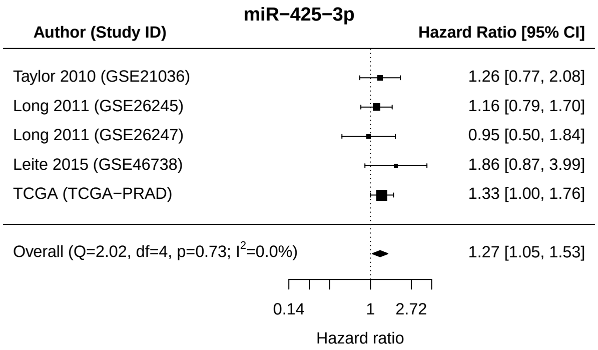

(p)

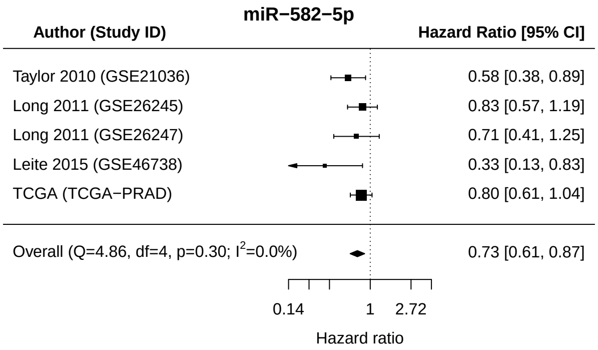

Figure S5: Association of miR expression with biochemical recurrence: a multivariate analysis. In total 16 miRs were significantly associated with biochemical recurrence even after adjusting for prognostic clinical markers Gleason score, tumour stage and serum PSA at diagnosis. The expression of all but three miRs (miR-130b-3p, miR-181b-5p, miR-425-3p) associated negatively with recurrence. "Overall" refers to the overall effect estimate, i.e. pooled hazard ratio. The "Q", "df" and "p" values refer to the statistics for Q-test for heterogeneity and the "I^2" value refers to the outcome of I^2 test for heterogeneity.

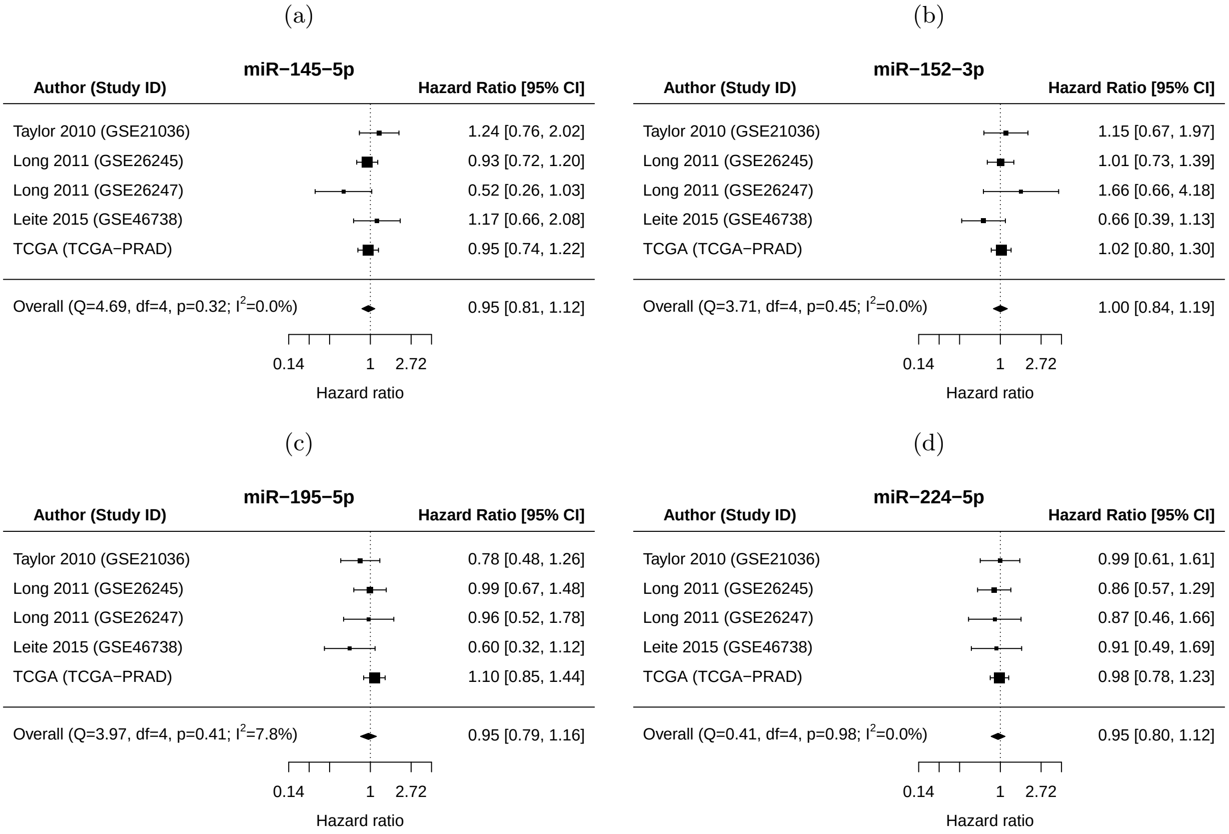

Figure S6: Association of the four miRs identified as prognostic in the systematic review with biochemical recurrence (multivariate analysis). let-7b-5p was not evaluated in the meta-analysis as it was not profiled in all five datasets.

References

[1] Maria Schubert, Martin Spahn, Susanne Kneitz, Claus Jürgen Scholz, Steven Joniau, Philipp Stroebel, et al. "Distinct microRNA Expression Profile in Prostate Cancer Patients with Early Clinical Failure and the Impact of let-7 as Prognostic Marker in High-Risk Prostate Cancer". In: *PLoS ONE* 8.6 (2013). ISSN: 19326203. DOI: 10.1371/journal.pone.0065064.

[2] Robert S. Hudson, Ming Yi, Dominic Esposito, Stephanie K. Watkins, Arthur A. Hurwitz, Harris G. Yfantis, et al. "MicroRNA-1 is a candidate tumor suppressor and prognostic marker in human prostate cancer". In: *Nucleic Acids Research* 40.8 (2012), pp. 3689–3703. ISSN: 03051048. DOI: 10.1093/nar/gkr1222.

[3] Katia R.M. Leite, Alberto Tomiyama, Sabrina T. Reis, Juliana M. Sousa-Canavez, Adriana Saudo, Marcos F. Dall'Oglio, et al. "MicroRNA-100 expression is independently related to biochemical recurrence of prostate cancer". In: *Journal of Urology* 185.3 (2011), pp. 1118–1122. ISSN: 00225347. DOI: 10.1016/j.juro.2010.10.035. URL: http://dx.doi.org/10.1016/j.juro.2010.10.035.

[4] Prashant K Singh, Leah Preus, Qiang Hu, Li Yan, Mark D Long, Carl D Morrison, et al. "Serum microRNA expression patterns that predict early treatment failure in prostate cancer patients." In: *Oncotarget* 5.3 (2014), pp. 824–40. ISSN: 1949-2553. DOI: 10.18632/oncotarget.1776. URL: http://www.pubmedcentral.nih.gov/articlerender.fcgi?artid=3996656%7B%5C&%7Dtool=pmcentrez%7B%5C&%7Drendertype=abstract.

[5] R. S. Hudson, M. Yi, D. Esposito, S. A. Glynn, A. M. Starks, Y. Yang, et al. "MicroRNA-106b-25 cluster expression is associated with early disease recurrence and targets caspase-7 and focal adhesion in human prostate cancer". In: *Oncogene* 32.35 (2013), pp. 4139–4147. ISSN: 09509232. DOI: 10.1038/onc.2012.424.

[6] Annika Fendler, Monika Jung, Carsten Stephan, Richardson J. Honey, Robert J. Stewart, Kenneth T. Pace, et al. "miRNAs can predict prostate cancer biochemical relapse and are

involved in tumor progression". In: *International Journal of Oncology* 39.5 (2011), pp. 1183–1192. ISSN: 10196439. DOI: 10.3892/ijo.2011.1128.

[7] Erica Hlavin Bell, Simon Kirste, Jessica L. Fleming, Petra Stegmaier, Vanessa Drendel, Xiaokui Mo, et al. "A novel MiRNA-based predictive model for biochemical failure following post-prostatectomy salvage radiation therapy". In: *PLoS ONE* 10.3 (2015), pp. 1–19. ISSN: 19326203. DOI: 10.1371/journal.pone.0118745.

[8] Dibash K. Das, Joseph R. Osborne, Hui Yi Lin, Jong Y. Park, and Olorunseun O. Ogunwobi. "miR-1207-3p is a novel prognostic biomarker of prostate cancer". In: *Translational Oncology* 9.3 (2016), pp. 236–241. ISSN: 19365233. DOI: 10.1016/j.tranon.2016.04.005. URL: http://dx.doi.org/10.1016/j.tranon.2016.04.005.

[9] Yitao Wang, Qingling Zhang, Baowei Guo, Jiao Feng, and Dan Zhao. "MiR-1231 Is Downregulated in Prostate Cancer with Prognostic and Functional Implications". In: *Oncology Research and Treatment* (2019). ISSN: 22965262. DOI: 10.1159/000504606.

[10] Annika Schaefer, Monika Jung, Hans Joachim Mollenkopf, Ina Wagner, Carsten Stephan, Florian Jentzmik, et al. "Diagnostic and prognostic implications of microRNA profiling in prostate carcinoma". In: *International Journal of Cancer* 126.5 (2010), pp. 1166–1176. ISSN: 00207136. DOI: 10.1002/ijc.24827.

[11] X Sun, Z Liu, Z Yang, L Xiao, F Wang, Y He, et al. "Association of microRNA-126 expression with clinicopathological features and the risk of biochemical recurrence in prostate cancer patients undergoing radical prostatectomy". In: *Diagnostic Pathology* 8 (2013), p. 208. URL: http://ovidsp.ovid.com/ovidweb.cgi?T=JS%7B%5C&%7DCSC=Y%7B%5C&%7DNEWS=N%7B%5C&%7DPAGE=fulltext%7B%5C&%7DD=medl%7B%5C&%7DAN=24350576%7B%5C%%7D5Cnhttp://digitaal.uba.uva.nl:9003/uva-linker?sid=OVID:medline%7B%5C&%7Did=pmid:24350576%7B%5C&%7Did=doi:10.1186%7B%5C%%7D2F1746-1596-8-208%7B%5C&%7Dissn=1746-1596%7B%5C&%7Disbn=%7B%5C&%7Dvolume=8%7B%5C&%7Dissue=%7B%5C&%7Dspage=208%7B%5C&%7Dpages=208.

[12] Xiaoke Sun, Zhen Yang, Yu Zhang, Jing He, Feng Wang, Pengxiao Su, et al. "Prognostic implications of tissue and serum levels of microRNA-128 in human prostate cancer". In: *International Journal of Clinical and Experimental Pathology* 8.7 (2015), pp. 8394–8401. ISSN: 19362625.

[13] Zhongchun Hu, Junjie Guo, Ming Zhao, Tao Jiang, and Xiaofeng Yang. "Predictive values of miR-129 and miR-139 for efficacy on patients with prostate cancer after chemotherapy and prognostic correlation". In: *Oncology Letters* 18.6 (2019), pp. 6187–6195. ISSN: 17921082. DOI: 10.3892/ol.2019.10950.

[14] Jorge Torres-Ferreira, João Ramalho-Carvalho, Antonio Gomez, Francisco Duarte Menezes, Rui Freitas, Jorge Oliveira, et al. "MiR-193b promoter methylation accurately detects prostate cancer in urine sediments and miR-34b/c or miR-129-2 promoter methylation define subsets of clinically aggressive tumors". In: *Molecular Cancer* 16.1 (2017), pp. 1–12. ISSN: 14764598. DOI: 10.1186/s12943-017-0604-0.

[15] Xiaoyi Huang, Tiezheng Yuan, Meihua Liang, Meijun Du, Shu Xia, Rachel Dittmar, et al. "Exosomal miR-1290 and miR-375 as prognostic markers in castration-resistant prostate cancer". In: *European Urology* 67.1 (2015), pp. 33–41. ISSN: 18737560. DOI: 10.1016/j.eururo.2014.07.035. URL: http://dx.doi.org/10.1016/j.eururo.2014.07.035.

[16] Bo Liu, Weidong Zhou, Huiyang Jiang, Zhendong Xiang, and Lei Wang. "miR-1303 promotes the proliferation, migration and invasion of prostate cancer cells through regulating the Wnt/β-catenin pathway by targeting DKK3". In: *Experimental and Therapeutic Medicine* 18.7 (2019), pp. 4747–4757. ISSN: 1792-0981. DOI: 10.3892/etm.2019.8120.

[17] Yutaka Hashimoto, Marisa Shiina, Pritha Dasgupta, Priyanka Kulkarni, Taku Kato, Ryan K. Wong, et al. "Upregulation of MIR-130b contributes to risk of poor prognosis and racial disparity in African-American Prostate Cancer". In: *Cancer Prevention Research* 12.9 (2019), pp. 585–598. ISSN: 19406215. DOI: 10.1158/1940-6207.CAPR-18-0509.

[18] Yubo Tang, Jincheng Pan, Shuai Huang, Xinsheng Peng, Xuenong Zou, Yongxiang Luo, et al. "Downregulation of miR-133a-3p promotes prostate cancer bone metastasis via activating

PI3K/AKT signaling”. In: *Journal of Experimental and Clinical Cancer Research* 37.1 (2018), pp. 1–16. ISSN: 17569966. DOI: 10.1186/s13046-018-0813-4.

[19] Xia Li, Xuechao Wan, Hongbing Chen, Shu Yang, Yiyang Liu, Wenjuan Mo, et al. “Identification of miR-133b and RB1CC1 as independent predictors for biochemical recurrence and potential therapeutic targets for prostate cancer”. In: *Clinical Cancer Research* 20.9 (2014), pp. 2312–2325. ISSN: 15573265. DOI: 10.1158/1078-0432.CCR-13-1588.

[20] Shuai Huang, Qingde Wa, Jincheng Pan, Xinsheng Peng, Dong Ren, Qiji Li, et al. “Transcriptional downregulation of miR-133b by REST promotes prostate cancer metastasis to bone via activating TGF-β signaling article”. In: *Cell Death and Disease* 9.7 (2018). ISSN: 20414889. DOI: 10.1038/s41419-018-0807-3.

[21] Robert K. Nam, Tania Benatar, Christopher J.D. Wallis, Elizabeth Kobylecky, Yutaka Amemiya, Christopher Sherman, et al. “MicroRNA-139 is a predictor of prostate cancer recurrence and inhibits growth and migration of prostate cancer cells through cell cycle arrest and targeting IGF1R and AXL”. In: *Prostate* 79.12 (2019), pp. 1422–1438. ISSN: 10970045. DOI: 10.1002/pros.23871.

[22] Elin Richardsen, Sigve Andersen, Christian Melbø-Jørgensen, Mehrdad Rakaee, Nora Ness, Samer Al-Saad, et al. “MicroRNA 141 is associated to outcome and aggressive tumor characteristics in prostate cancer”. In: *Scientific Reports* 9.1 (2019), pp. 1–9. ISSN: 20452322. DOI: 10.1038/s41598-018-36854-7.

[23] Zhongwei Zhao, Sabine Weickmann, Monika Jung, Michael Lein, Ergin Kilic, Carsten Stephan, et al. “A novel predictor tool of biochemical recurrence after radical prostatectomy based on a five-microRNA tissue signature”. In: *Cancers* 11.10 (2019). ISSN: 20726694. DOI: 10.3390/cancers11101603.

[24] Ahmed Hussein Zedan, Søren Garm Blavnsfeldt, Torben Frøstrup Hansen, Boye Schnack Nielsen, Niels Marcussen, Mindaugas Pleckaitis, et al. “Heterogeneity of miRNA expression in localized

prostate cancer with clinicopathological correlations". In: *PLoS ONE* 12.6 (2017), pp. 1–17. ISSN: 19326203. DOI: 10.1371/journal.pone.0179113.

[25] Dongyang Li, Xuanyu Hao, and Yongsheng Song. "Identification of the Key MicroRNAs and the miRNA-mRNA Regulatory Pathways in Prostate Cancer by Bioinformatics Methods". In: *BioMed Research International* 2018 (2018). ISSN: 23146141. DOI: 10.1155/2018/6204128.

[26] M. Avgeris, K. Stravodimos, E. G. Fragoulis, and A. Scorilas. "The loss of the tumour-suppressor miR-145 results in the shorter disease-free survival of prostate cancer patients". In: *British Journal of Cancer* 108.12 (2013), pp. 2573–2581. ISSN: 00070920. DOI: 10.1038/bjc.2013.250.

[27] Xueqin Chen, Jing Gong, Hao Zeng, Ni Chen, Rui Huang, Ying Huang, et al. "MicroRNA145 targets BNIP3 and suppresses prostate cancer progression". In: *Cancer Research* 70.7 (2010), pp. 2728–2738. ISSN: 00085472. DOI: 10.1158/0008-5472.CAN-09-3718.

[28] Olivia Larne, Zandra Hagman, Hans Lilja, Anders Bjartell, Anders Edsjö, and Yvonne Ceder. "miR-145 suppress the androgen receptor in prostate cancer cells and correlates to prostate cancer prognosis". In: *Carcinogenesis* 36.8 (2015), pp. 858–866. ISSN: 14602180. DOI: 10.1093/carcin/bgv063.

[29] Sung Gu Kang, Young Ran Ha, Seo Jin Kim, Seok Ho Kang, Hong Seok Park, Jeong Gu Lee, et al. "Do microRNA 96, 145 and 221 expressions really aid in the prognosis of prostate carcinoma?" In: *Asian Journal of Andrology* 14.5 (2012), pp. 752–757. ISSN: 1008682X. DOI: 10.1038/aja.2012.68.

[30] L. A. Selth, S. L. Townley, A. G. Bert, P. D. Stricker, P. D. Sutherland, L. G. Horvath, et al. "Circulating microRNAs predict biochemical recurrence in prostate cancer patients". In: *British Journal of Cancer* 109.3 (2013), pp. 641–650. ISSN: 00070920. DOI: 10.1038/bjc.2013.369.

[31] Bin Xu, Yeqing Huang, Xiaobing Niu, Tao Tao, Liang Jiang, Na Tong, et al. "Hsa-miR-146a-5p modulates androgen-independent prostate cancer cells apoptosis by targeting ROCK1". In: *Prostate* 75.16 (2015), pp. 1896–1903. ISSN: 10970045. DOI: 10.1002/pros.23068.

[32] M. Chen, Z. Y. Zhou, J. G. Chen, N. Tong, S. Q. Chen, Y. Yang, et al. "Effect of miR-146a polymorphism on biochemical recurrence risk after radical prostatectomy in southern Chinese population". In: *Genetics and Molecular Research* 13.4 (2014), pp. 10615–10621. ISSN: 16765680. DOI: 10.4238/2014.December.18.3.

[33] Jingsong Yu, Yue Feng, Yan Wang, and Ruihua An. "Aryl hydrocarbon receptor enhances the expression of miR-150-5p to suppress in prostate cancer progression by regulating MAP3K12". In: *Archives of Biochemistry and Biophysics* 654.23 (2018), pp. 47–54. ISSN: 10960384. DOI: 10.1016/j.abb.2018.07.010. URL: https://doi.org/10.1016/j.abb.2018.07.010.

[34] Shaniece C Theodore, Melissa Davis, Fu Zhao, Honghe Wang, Dongquan Chen, Johng Rhim, et al. "MicroRNA profiling of novel African American and Caucasian Prostate Cancer cell lines reveals a reciprocal regulatory relationship of miR-152 and DNA methyltranferase 1". In: *Oncotarget* 5.11 (2014), pp. 3512–25.

[35] João Ramalho-Carvalho, Céline S. Gonçalves, Inês Graça, David Bidarra, Eva Pereira-Silva, Sofia Salta, et al. "A multiplatform approach identifies miR-152-3p as a common epigenetically regulated onco-suppressor in prostate cancer targeting TMEM97". In: *Clinical Epigenetics* 10.1 (2018), pp. 1–15. ISSN: 18687083. DOI: 10.1186/s13148-018-0475-2.

[36] Cheng Wei Bi, Guo Ying Zhang, Yu Bai, Bin Zhao, and Hong Yang. "Increased expression of miR-153 predicts poor prognosis for patients with prostate cancer". In: *Medicine (United States)* 98.36 (2019), pp. 1–4. ISSN: 15365964. DOI: 10.1097/MD.0000000000016705.

[37] Ran Chen, Lu Sheng, Hao Jie Zhang, Ming Ji, and Wei Qing Qian. "miR-15b-5p facilitates the tumorigenicity by targeting RECK and predicts tumour recurrence in prostate cancer". In: *Journal of Cellular and Molecular Medicine* 22.3 (2018), pp. 1855–1863. ISSN: 15821838. DOI: 10.1111/jcmm.13469.

[38] Zhen Tao, Shaohua Xu, Hailong Ruan, Tao Wang, Wen Song, Li Qian, et al. "MiR-195/-16 Family Enhances Radiotherapy via T Cell Activation in the Tumor Microenvironment by Block-

ing the PD-L1 Immune Checkpoint”. In: *Cellular Physiology and Biochemistry* 48.2 (2018), pp. 801–814. ISSN: 14219778. DOI: 10.1159/000491909.

[39] C. Hoey, M. Ahmed, A. Fotouhi Ghiam, D. Vesprini, X. Huang, K. Commisso, et al. “Circulating miRNAs as non-invasive biomarkers to predict aggressive prostate cancer after radical prostatectomy”. In: *Journal of Translational Medicine* 17.1 (2019), pp. 1–11. ISSN: 14795876. DOI: 10.1186/s12967-019-1920-5.

[40] Yanan Sun, Xiaopeng Jia, Lianguo Hou, and Xing Liu. “Screening of Differently Expressed miRNA and mRNA in Prostate Cancer by Integrated Analysis of Transcription Data”. In: *Urology* 94 (2016), 313.e1–313.e6. ISSN: 15279995. DOI: 10.1016/j.urology.2016.04.041. URL: http://dx.doi.org/10.1016/j.urology.2016.04.041.

[41] Hiroshi Hirata, Koji Ueno, Varahram Shahryari, Guoren Deng, Yuichiro Tanaka, Z. Laura Tabatabai, et al. “MicroRNA-182-5p Promotes Cell Invasion and Proliferation by Down Regulating FOXF2, RECK and MTSS1 Genes in Human Prostate Cancer”. In: *PLoS ONE* 8.1 (2013). ISSN: 19326203. DOI: 10.1371/journal.pone.0055502.

[42] Irene Casanova-Salas, José Rubio-Briones, Ana Calatrava, Caterina Mancarella, Esther Masiá, Juan Casanova, et al. “Identification of miR-187 and miR-182 as biomarkers of early diagnosis and prognosis in patients with prostate cancer treated with radical prostatectomy”. In: *Journal of Urology* 192.1 (2014), pp. 252–259. ISSN: 15273792. DOI: 10.1016/j.juro.2014.01.107.

[43] Xian Zhao, Yanli Wang, Rong Deng, Hailong Zhang, Jinzhuo Dou, Haihua Yuan, et al. “miR186 suppresses prostate cancer progression by targeting Twist1”. In: *Oncotarget* 7.22 (2016), pp. 33136–33151. ISSN: 19492553. DOI: 10.18632/oncotarget.8887.

[44] Hongtuan Zhang, Shiyong Qi, Tao Zhang, Andi Wang, Ranlu Liu, Jia Guo, et al. “miR-188-5p inhibits tumour growth and metastasis in prostate cancer by repressing LAPTM4B expression”. In: *Oncotarget* 6.8 (2015), pp. 6092–6104. ISSN: 19492553. DOI: 10.18632/oncotarget.3341.

[45] Shaohua Xu, Tao Wang, Wen Song, Tao Jiang, Feng Zhang, Yu Yin, et al. “The inhibitory effects of AR/miR-190a/YB-1 negative feedback loop on prostate cancer and underlying mecha-

nism". In: *Scientific Reports* 5.July (2015), pp. 1–14. ISSN: 20452322. DOI: 10.1038/srep13528. URL: http://dx.doi.org/10.1038/srep13528.

[46] Jing Bo Liu, Yong Ji Yan, Jing Shi, Ya Bing Wu, Yan Feng Li, Lin Feng Dai, et al. "Upregulation of microRNA-191 can serve as an independent prognostic marker for poor survival in prostate cancer". In: *Medicine* 98.29 (2019), e16193. ISSN: 15365964. DOI: 10.1097/MD.0000000000016193.

[47] Zhong Jun Chen, You Ji Yan, Hao Shen, Jia Jie Zhou, Guang Hua Yang, Yi Xiang Liao, et al. "MiR-192 Is Overexpressed and Promotes Cell Proliferation in Prostate Cancer". In: *Medical Principles and Practice* 28.2 (2019), pp. 124–132. ISSN: 14230151. DOI: 10.1159/000496206.

[48] Jia Guo, Min Wang, and Xiuheng Liu. "MicroRNA-195 suppresses tumor cell proliferation and metastasis by directly targeting BCOX1 in prostate carcinoma". In: *Journal of Experimental and Clinical Cancer Research* 34.1 (2015), pp. 1–8. ISSN: 17569966. DOI: 10.1186/s13046-015-0209-7. URL: http://dx.doi.org/10.1186/s13046-015-0209-7.

[49] Chao Cai, Qing Biao Chen, Zhao Dong Han, Yan Qiong Zhang, Hui Chan He, Jia Hong Chen, et al. "miR-195 inhibits tumor progression by targeting RPS6KB1 in human prostate cancer". In: *Clinical Cancer Research* 21.21 (2015), pp. 4922–4934. ISSN: 15573265. DOI: 10.1158/1078-0432.CCR-15-0217.

[50] Chunhui Liu, Han Guan, Yiduo Wang, Ming Chen, Bin Xu, Lei Zhang, et al. "MIR-195 inhibits emt by targeting FGF2 in prostate cancer cells". In: *PLoS ONE* 10.12 (2015), pp. 1–13. ISSN: 19326203. DOI: 10.1371/journal.pone.0144073.

[51] Xiaowen Zhang, Tao Tao, Chunhui Liu, Han Guan, Yeqing Huang, Bin Xu, et al. "Downregulation of miR-195 promotes prostate cancer progression by targeting HMGA1". In: *Oncology Reports* 36.1 (2016), pp. 376–382. ISSN: 17912431. DOI: 10.3892/or.2016.4797.

[52] Fangqiu Fu, Xuechao Wan, Dan Wang, Zhe Kong, Yalong Zhang, Wenhua Huang, et al. "MicroRNA-19a acts as a prognostic marker and promotes prostate cancer progression via

inhibiting VPS37A expression". In: *Oncotarget* 9.2 (2018), pp. 1931–1943. ISSN: 19492553. DOI: 10.18632/oncotarget.23026.

[53] Betina Katz, Sabrina T. Reis, Nayara I. Viana, Denis R. Morais, Caio M. Moura, Nelson Dip, et al. "Comprehensive study of gene and microRNA expression related to epithelial-mesenchymal transition in prostate cancer". In: *PLoS ONE* 9.11 (2014). ISSN: 19326203. DOI: 10.1371/journal.pone.0113700.

[54] Ziling Huang, Long Zhang, Xianghua Yi, and Xiaoting Yu. "Diagnostic and prognostic values of tissue hsa-miR-30c and hsa-miR-203 in prostate carcinoma". In: *Tumor Biology* 37.4 (2016), pp. 4359–4365. ISSN: 14230380. DOI: 10.1007/s13277-015-4262-9.

[55] Qingde Wa, Sheng Huang, Jincheng Pan, Yubo Tang, Shaofu He, Xiaodong Fu, et al. "miR-204-5p Represses Bone Metastasis via Inactivating NF-κB Signaling in Prostate Cancer". In: *Molecular Therapy - Nucleic Acids* 18.December (2019), pp. 567–579. ISSN: 21622531. DOI: 10.1016/j.omtn.2019.09.008. URL: https://doi.org/10.1016/j.omtn.2019.09.008.

[56] Yngve Nordby, Elin Richardsen, Nora Ness, Tom Donnem, Hiten R.H. Patel, Lill Tove Busund, et al. "High miR-205 expression in normal epithelium is associated with biochemical failure - An argument for epithelial crosstalk in prostate cancer?" In: *Scientific Reports* 7.1 (2017), pp. 1–10. ISSN: 20452322. DOI: 10.1038/s41598-017-16556-2. URL: http://dx.doi.org/10.1038/s41598-017-16556-2.

[57] Z. Hagman, B. S. Haflidadóttir, J. A. Ceder, O. Larne, A. Bjartell, H. Lilja, et al. "MiR-205 negatively regulates the androgen receptor and is associated with adverse outcome of prostate cancer patients". In: *British Journal of Cancer* 108.8 (2013), pp. 1668–1676. ISSN: 00070920. DOI: 10.1038/bjc.2013.131.

[58] T. Hulf, T. Sibbritt, E. D. Wiklund, K. Patterson, J. Z. Song, C. Stirzaker, et al. "Epigenetic-induced repression of microRNA-205 is associated with MED1 activation and a poorer prognosis in localized prostate cancer". In: *Oncogene* 32.23 (2013), pp. 2891–2899. ISSN: 09509232. DOI: 10.1038/onc.2012.300.

[59] Charis Kalogirou, Martin Spahn, Markus Krebs, Steven Joniau, Evelyne Lerut, Maximilian Burger, et al. "MiR-205 is progressively down-regulated in lymph node metastasis but fails as a prognostic biomarker in high-risk prostate cancer". In: *International Journal of Molecular Sciences* 14.11 (2013), pp. 21414–21434. ISSN: 16616596. DOI: 10.3390/ijms141121414.

[60] Christian Melbø-Jørgensen, Sigve Andersen, Andrej Valkov, Tom Dønnem, Samer Al-Saad, Yury Kiselev, et al. "Stromal expression of miR-21 predicts biochemical failure in prostate cancer patients with Gleason score 6". In: *PLoS ONE* 9.11 (2014). ISSN: 19326203. DOI: 10.1371/journal.pone.0113039.

[61] Tao Li, Run Sheng Li, Yu Hua Li, Shang Zhong, Yu Ying Chen, Cun Ming Zhang, et al. "MiR-21 as an independent biochemical recurrence predictor and potential therapeutic target for prostate cancer". In: *Journal of Urology* 187.4 (2012), pp. 1466–1472. ISSN: 00225347. DOI: 10.1016/j.juro.2011.11.082.

[62] Yangbo Guan, You Wu, Yifei Liu, Jian Ni, and Shaojun Nong. "Association of microRNA-21 expression with clinicopathological characteristics and the risk of progression in advanced prostate cancer patients receiving androgen deprivation therapy". In: *The Prostate* 76.11 (2016), pp. 986–993. ISSN: 02704137. DOI: 10.1002/pros.23187. URL: http://doi.wiley.com/10.1002/pros.23187.

[63] Ernest K. Amankwah, Evelyn Anegbe, Hyun Park, Julio Pow-Sang, Ardeshir Hakam, and Jong Y. Park. "MiR-21, miR-221 and miR-222 expression and prostate cancer recurrence among obese and non-obese cases". In: *Asian Journal of Andrology* 15.2 (2013), pp. 226–230. ISSN: 1008682X. DOI: 10.1038/aja.2012.160. URL: http://dx.doi.org/10.1038/aja.2012.160.

[64] Sigve Andersen, Elin Richardsen, Line Moi, Tom Donnem, Yngve Nordby, Nora Ness, et al. "Fibroblast miR-210 overexpression is independently associated with clinical failure in Prostate Cancer - A multicenter (in situ hybridization) study". In: *Scientific Reports* 6.October (2016), pp. 1–9. ISSN: 20452322. DOI: 10.1038/srep36573.

[65] H. W. Qu, Y. Jin, Z. L. Cui, and X. B. Jin. "MicroRNA-212 participates in the development of prostate cancer by upregulating BMI1 via NF-κB pathway". In: *European review for medical and pharmacological sciences* 22.11 (2018), pp. 3348–3356. ISSN: 22840729. DOI: 10.26355/eurrev_201806_15155.

[66] Peng Peng, Tao Chen, Qing Wang, Yixi Zhang, Fangfang Zheng, Shuai Huang, et al. "Decreased miR-218-5p Levels as a Serum Biomarker in Bone Metastasis of Prostate Cancer". In: *Oncology Research and Treatment* 42.4 (2019), pp. 165–180. ISSN: 22965262. DOI: 10.1159/000495473.

[67] Martin Spahn, Susanne Kneitz, Claus Jürgen Scholz, Nico Stenger, Thomas Rüdiger, Philipp Ströbel, et al. "Expression of microRNA-221 is progressively reduced in aggressive prostate cancer and metastasis and predicts clinical recurrence". In: *International Journal of Cancer* 127.2 (2010), pp. 394–403. ISSN: 00207136. DOI: 10.1002/ijc.24715.

[68] Juliana I. Santos, Ana L. Teixeira, Francisca Dias, Joaquina Maurício, Francisco Lobo, António Morais, et al. "Influence of peripheral whole-blood microRNA-7 and microRNA-221 high expression levels on the acquisition of castration-resistant prostate cancer: Evidences from in vitro and in vivo studies". In: *Tumor Biology* 35.7 (2014), pp. 7105–7113. ISSN: 14230380. DOI: 10.1007/s13277-014-1918-9.

[69] Yusuke Goto, Satoko Kojima, Rika Nishikawa, Akira Kurozumi, Mayuko Kato, Hideki Enokida, et al. "MicroRNA expression signature of castration-resistant prostate cancer: The microRNA-221/222 cluster functions as a tumour suppressor and disease progression marker". In: *British Journal of Cancer* 113.7 (2015), pp. 1055–1065. ISSN: 15321827. DOI: 10.1038/bjc.2015.300.

[70] Zhuo Yuan Lin, Ya Qiang Huang, Yan Qiong Zhang, Zhao Dong Han, Hui Chan He, Xiao Hui Ling, et al. "MicroRNA-224 inhibits progression of human prostate cancer by downregulating TRIB1". In: *International Journal of Cancer* 135.3 (2014), pp. 541–550. ISSN: 10970215. DOI: 10.1002/ijc.28707.

[71] Konstantinos Mavridis, Konstantinos Stravodimos, and Andreas Scorilas. "Downregulation and prognostic performance of microRNA 224 expression in prostate cancer". In: *Clinical Chemistry* 59.1 (2013), pp. 261–269. ISSN: 00099147. DOI: 10.1373/clinchem.2012.191502.

[72] Songwang Cai, Ruihan Chen, Xiaojuan Li, Yi Cai, Zhiqiang Ye, Shigeng Li, et al. "Down-regulation of microRNA-23a suppresses prostate cancer metastasis by targeting the PAK6-LIMK1 signaling pathway". In: *Oncotarget* 6.6 (2015), pp. 3904–3917. ISSN: 19492553. DOI: 10.18632/oncotarget.2880.

[73] Minhao Zhang, Yali Wang, Can Wang, Zonghao You, Shuqiu Chen, Qingfang Kong, et al. "Association of Hsa-miR-23a rs3745453 variation with prostate cancer risk among Chinese Han population: A case-control study". In: *Medicine (United States)* 98.52 (2019). ISSN: 15365964. DOI: 10.1097/MD.0000000000018523.

[74] Shahana Majid, Altaf A. Dar, Sharanjot Saini, Sumit Arora, Varahram Shahryari, Mohd Saif Zaman, et al. "miR-23b represses proto-oncogene Src kinase and functions as methylation-silenced tumor suppressor with diagnostic and prognostic significance in prostate cancer". In: *Cancer Research* 72.24 (2012), pp. 6435–6446. ISSN: 00085472. DOI: 10.1158/0008-5472.CAN-12-2181.

[75] Kai Guo, Shaobo Zheng, Yawen Xu, Abai Xu, Binshen Chen, and Yong Wen. "Loss of miR-26a-5p promotes proliferation, migration, and invasion in prostate cancer through negatively regulating SERBP1". In: *Tumor Biology* 37.9 (2016), pp. 12843–12854. ISSN: 14230380. DOI: 10.1007/s13277-016-5158-z. URL: http://dx.doi.org/10.1007/s13277-016-5158-z.

[76] Weiyin Gao, Zhengdong Hong, Hongwei Huang, Anyi Zhu, Shuangquan Lin, Cheng Cheng, et al. "miR-27a in serum acts as biomarker for prostate cancer detection and promotes cell proliferation by targeting Sprouty2". In: *Oncology Letters* 16.4 (2018), pp. 5291–5298. ISSN: 17921082. DOI: 10.3892/ol.2018.9274.

[77] Yusuke Goto, Satoko Kojima, Rika Nishikawa, Hideki Enokida, Takeshi Chiyomaru, Takashi Kinoshita, et al. "The microRNA-23b/27b/24-1 cluster is a disease progression marker and tu-

mor suppressor in prostate cancer". In: *Oncotarget* 5.17 (2014), pp. 7748–7759. ISSN: 19492553. DOI: 10.18632/oncotarget.2294.

[78] Robert K. Nam, Tania Benatar, Christopher J.D. Wallis, Yutaka Amemiya, Wenyi Yang, Alaina Garbens, et al. "MiR-301a regulates E-cadherin expression and is predictive of prostate cancer recurrence". In: *Prostate* 76.10 (2016), pp. 869–884. ISSN: 10970045. DOI: 10.1002/pros.23177.

[79] Xiao Hui Ling, Zhao Dong Han, Dan Xia, Hui Chan He, Fu Neng Jiang, Zhuo Yuan Lin, et al. "MicroRNA-30c serves as an independent biochemical recurrence predictor and potential tumor suppressor for prostate cancer". In: *Molecular Biology Reports* 41.5 (2014), pp. 2779–2788. ISSN: 15734978. DOI: 10.1007/s11033-014-3132-7.

[80] Naohito Kobayashi, Hiroji Uemura, Kiyotaka Nagahama, Koji Okudela, Mitsuko Furuya, Yoko Ino, et al. "Identification of miR-30d as a novel prognostic maker of prostate cancer". In: *Oncotarget* 3.11 (2012), pp. 1455–1471. ISSN: 19492553. DOI: 10.18632/oncotarget.696.

[81] Zhuo yuan Lin, Guo Chen, Yan qiong Zhang, Hui chan He, Yu xiang Liang, Jian heng Ye, et al. "MicroRNA-30d promotes angiogenesis and tumor growth via MYPT1/c-JUN/VEGFA pathway and predicts aggressive outcome in prostate cancer". In: *Molecular Cancer* 16.1 (2017), pp. 1–14. ISSN: 14764598. DOI: 10.1186/s12943-017-0615-x.

[82] Xuan Liang, Zhaolun Li, Qunli Men, Yongwei Li, Hechen Li, and Tie Chong. "miR-326 functions as a tumor suppressor in human prostatic carcinoma by targeting Mucin1". In: *Biomedicine and Pharmacotherapy* 108.May (2018), pp. 574–583. ISSN: 19506007. DOI: 10.1016/j.biopha.2018.09.053. URL: https://doi.org/10.1016/j.biopha.2018.09.053.

[83] Si Wei Xiong, Tian Xin Lin, Ke Wei Xu, Wen Dong, Xiao Hui Ling, Fu Neng Jiang, et al. "MicroRNA-335 acts as a candidate tumor suppressor in prostate cancer". In: *Pathology and Oncology Research* 19.3 (2013), pp. 529–537. ISSN: 12194956. DOI: 10.1007/s12253-013-9613-5.

[84] Ashraf Bakkar, Mohammed Alshalalfa, Lars F. Petersen, Hatem Abou-Ouf, Amal Al-Mami, Samar A. Hegazy, et al. "microRNA 338-3p exhibits tumor suppressor role and its down-

regulation is associated with adverse clinical outcome in prostate cancer patients". In: *Molecular Biology Reports* 43.4 (2016), pp. 229–240. ISSN: 15734978. DOI: 10.1007/s11033-016-3948-4.

[85] Shahana Majid, Altaf A. Dar, Sharanjot Saini, Varahram Shahryari, Sumit Arora, Mohd Saif Zaman, et al. "miRNA-34b inhibits prostate cancer through demethylation, active chromatin modifications, and AKT pathways". In: *Clinical Cancer Research* 19.1 (2013), pp. 73–84. ISSN: 10780432. DOI: 10.1158/1078-0432.CCR-12-2952.

[86] Zandra Hagman, Olivia Larne, Anders Edsjö, Anders Bjartell, Roy A. Ehrnström, David Ulmert, et al. "MiR-34c is downregulated in prostate cancer and exerts tumor suppressive functions". In: *International Journal of Cancer* 127.12 (2010), pp. 2768–2776. ISSN: 00207136. DOI: 10.1002/ijc.25269.

[87] Sharanjot Saini, Shahana Majid, Varahram Shahryari, Z. Laura Tabatabai, Sumit Arora, Soichiro Yamamura, et al. "Regulation of SRC kinases by microRNA-3607 located in a frequently deleted locus in prostate cancer". In: *Molecular Cancer Therapeutics* 13.7 (2014), pp. 1952–1963. ISSN: 15388514. DOI: 10.1158/1535-7163.MCT-14-0017.

[88] Nathan Bucay, Divya Bhagirath, Kirandeep Sekhon, Thao Yang, Shinichiro Fukuhara, Shahana Majid, et al. "A novel microRNA regulator of prostate cancer epithelial-mesenchymal transition". In: *Cell Death and Differentiation* 24.7 (2017), pp. 1263–1274. ISSN: 14765403. DOI: 10.1038/cdd.2017.69.

[89] Nathan Bucay, Kirandeep Sekhon, Shahana Majid, Soichiro Yamamura, Varahram Shahryari, Z. Laura Tabatabai, et al. "Novel tumor suppressor microRNA at frequently deleted chromosomal region 8p21 regulates Epidermal Growth Factor Receptor in prostate cancer". In: *Oncotarget* 7.43 (2016), pp. 70388–70403. ISSN: 19492553. DOI: 10.18632/oncotarget.11865.

[90] H. W. Qu, Y. Jin, Z. L. Cui, and X. B. Jin. "MicroRNA-373-3p inhibits prostate cancer progression by targeting AKT1". In: *European Review for Medical and Pharmacological Sciences* 22.19 (2018), pp. 6252–6259. ISSN: 22840729. DOI: 10.26355/eurrev-201810-16032.

[91] Hui-chan He, Zhao-dong Han, Qi-shan Dai, Xiao-hui Ling, Xin Fu, Zhuo-yuan Lin, et al. "Global analysis of the differentially expressed miRNAs of prostate cancer in Chinese patients". In: *BMC Genomics* 14.1 (2013), p. 757. ISSN: 1471-2164. DOI: 10.1186/1471-2164-14-757. URL: http://bmcgenomics.biomedcentral.com/articles/10.1186/1471-2164-14-757.

[92] Margaritis Avgeris, Konstantinos Stravodimos, and Andreas Scorilas. "Loss of miR-378 in prostate cancer, a common regulator of KLK2 and KLK4, correlates with aggressive disease phenotype and predicts the short-term relapse of the patients". In: *Biological Chemistry* 395.9 (2014), pp. 1095–1104. ISSN: 14374315. DOI: 10.1515/hsz-2014-0150.

[93] Murali Gururajan, Sajni Josson, Gina Chia Yi Chu, Chia Lun Lu, Yi Tsung Lu, Christopher L. Haga, et al. "MiR-154* and miR-379 in the DLK1-DIO3 MicroRNA mega-cluster regulate epithelial to mesenchymal transition and bone metastasis of prostate cancer". In: *Clinical Cancer Research* 20.24 (2014), pp. 6559–6569. ISSN: 15573265. DOI: 10.1158/1078-0432.CCR-14-1784.

[94] Sajni Josson, Murali Gururajan, Peizhen Hu, Chen Shao, Gina Chia Yi Chu, Haiyen E. Zhau, et al. "miR-409-3p/-5p promotes tumorigenesis, epithelial-to-mesenchymal transition, and bone metastasis of human prostate cancer". In: *Clinical cancer research : an official journal of the American Association for Cancer Research* 20.17 (2014), pp. 4636–4646. ISSN: 10780432. DOI: 10.1158/1078-0432.CCR-14-0305.

[95] Yuelong Zhang, Dahong Zhang, Jia Lv, Shuai Wang, and Qi Zhang. "miR-410–3p promotes prostate cancer progression via regulating PTEN/AKT/mTOR signaling pathway". In: *Biochemical and Biophysical Research Communications* 503.4 (2018), pp. 2459–2465. ISSN: 10902104. DOI: 10.1016/j.bbrc.2018.06.176. URL: https://doi.org/10.1016/j.bbrc.2018.06.176.

[96] Bo Ying Bao, Jiunn Bey Pao, Chun Nung Huang, Yeong Shiau Pu, Ta Yuan Chang, Yu Hsuan Lan, et al. "Polymorphisms inside MicroRNAs and MicroRNA target sites predict clinical outcomes in prostate cancer patients receiving androgen-deprivation therapy". In: *Clinical Cancer Research* 17.4 (2011), pp. 928–936. ISSN: 10780432. DOI: 10.1158/1078-0432.CCR-10-2648.

[97] E. Richardsen, S. Andersen, S. Al-Saad, M. Rakaee, Y. Nordby, M. I. Pedersen, et al. "Low Expression of miR-424-3p is Highly Correlated with Clinical Failure in Prostate Cancer". In: *Scientific Reports* 9.1 (2019), pp. 1–10. ISSN: 20452322. DOI: 10.1038/s41598-019-47234-0.

[98] DIvya Bhagirath, Thao Ly Yang, Z. Laura Tabatabai, Varahram Shahryari, Shahana Majid, Rajvir Dahiya, et al. "Role of a novel race-related tumor suppressor microRNA located in frequently deleted chromosomal locus 8p21 in prostate cancer progression". In: *Carcinogenesis* 40.5 (2019), pp. 633–642. ISSN: 14602180. DOI: 10.1093/carcin/bgz058.

[99] X. Lin and Y. Wang. "Re-expression of microRNA-4319 inhibits growth of prostate cancer via Her-2 suppression". In: *Clinical and Translational Oncology* 20.11 (2018), pp. 1400–1407. ISSN: 16993055. DOI: 10.1007/s12094-018-1871-y. URL: https://doi.org/10.1007/s12094-018-1871-y.

[100] Martin Mørck Mortensen, Søren Høyer, Torben Falck Ørntoft, Karina Dalsgaard Sørensen, Lars Dyrskjøt, and Michael Borre. "High miR-449b expression in prostate cancer is associated with biochemical recurrence after radical prostatectomy". In: *BMC Cancer* 14.1 (2014), p. 859. ISSN: 1471-2407. DOI: 10.1186/1471-2407-14-859. URL: http://bmccancer.biomedcentral.com/articles/10.1186/1471-2407-14-859.

[101] Yusuke Goto, Satoko Kojima, Akira Kurozumi, Mayuko Kato, Atsushi Okato, Ryosuke Matsushita, et al. "Regulation of E3 ubiquitin ligase-1 (WWP1) by microRNA-452 inhibits cancer cell migration and invasion in prostate cancer". In: *British Journal of Cancer* 114.10 (2016), pp. 1135–1144. ISSN: 15321827. DOI: 10.1038/bjc.2016.95.

[102] Hannah Nip, Altaf A. Dar, Sharanjot Saini, Melissa Colden, Shahryari Varahram, Harshika Chowdhary, et al. "Oncogenic microRNA-4534 regulates PTEN pathway in prostate cancer". In: *Oncotarget* 7.42 (2016), pp. 68371–68384. ISSN: 19492553. DOI: 10.18632/ONCOTARGET.12031.

[103] Qianwei Xing, Huyang Xie, Bingye Zhu, Zhiwei Sun, and Yeqing Huang. "MiR-455-5p suppresses the progression of prostate cancer by targeting CCR5". In: *BioMed Research International* 2019 (2019). ISSN: 23146141. DOI: 10.1155/2019/6394784.

[104] Melissa Colden, Altaf A. Dar, Sharanjot Saini, Priya V. Dahiya, Varahram Shahryari, Soichiro Yamamura, et al. "MicroRNA-466 inhibits tumor growth and bone metastasis in prostate cancer by direct regulation of osteogenic transcription factor RUNX2". In: *Cell Death and Disease* 8.1 (2017), pp. 1–11. ISSN: 20414889. DOI: 10.1038/cddis.2017.15. URL: http://dx.doi.org/10.1038/cddis.2017.15.

[105] Sumit Arora, Sharanjot Saini, Shinichiro Fukuhara, Shahana Majid, Varahram Shahryari, Soichiro Yamamura, et al. "MicroRNA-4723 inhibits prostate cancer growth through inactivation of the abelson family of nonreceptor protein tyrosine kinases". In: *PLoS ONE* 8.11 (2013). ISSN: 19326203. DOI: 10.1371/journal.pone.0078023.

[106] Bing Cai, Wei Chen, Yue Pan, Hongde Chen, Yirong Zhang, Zhiliang Weng, et al. "Inhibition of microRNA-500 has anti-cancer effect through its conditional downstream target of TFPI in human prostate cancer". In: *Prostate* 77.10 (2017), pp. 1057–1065. ISSN: 10970045. DOI: 10.1002/pros.23361.

[107] Xingkang Jiang, Yue Chen, E. Du, Kuo Yang, Zhihong Zhang, Shiyong Qi, et al. "GATA3-driven expression of miR-503 inhibits prostate cancer progression by repressing ZNF217 expression". In: *Cellular Signalling* 28.9 (2016), pp. 1216–1224. ISSN: 18733913. DOI: 10.1016/j.cellsig.2016.06.002.

[108] Yubo Tang, Bowen Wu, Shuai Huang, Xinsheng Peng, Xing Li, Xiufang Huang, et al. "Downregulation of miR-505-3p predicts poor bone metastasis-free survival in prostate cancer". In: *Oncology Reports* 41.1 (2019), pp. 57–66. ISSN: 17912431. DOI: 10.3892/or.2018.6826.

[109] Xiyan Zhang, Jian Zhou, Dongwei Xue, Zhi Li, Yili Liu, and Liming Dong. "MiR-515-5p acts as a tumor suppressor via targeting TRIP13 in prostate cancer". In: *International Journal of*

Biological Macromolecules 129 (2019), pp. 227–232. ISSN: 18790003. DOI: 10.1016/j.ijbiomac.2019.01.127. URL: https://doi.org/10.1016/j.ijbiomac.2019.01.127.

[110] Jayant K. Rane, Mauro Scaravilli, Antti Ylipää, Davide Pellacani, Vincent M. Mann, Matthew S. Simms, et al. "MicroRNA expression profile of primary prostate cancer stem cells as a source of biomarkers and therapeutic targets". In: *European Urology* 67.1 (2015), pp. 7–10. ISSN: 18737560. DOI: 10.1016/j.eururo.2014.09.005.

[111] Lin Wang, Guanhua Song, Weiwei Tan, Mei Qi, Lili Zhang, Jonathan Chan, et al. "miR-573 inhibits prostate cancer metastasis by regulating epithelial-mesenchymal transition". In: *Oncotarget* 6.34 (2015). URL: www.impactjournals.com/oncotarget/.

[112] Shuai Huang, Changye Zou, Yubo Tang, Qingde Wa, Xinsheng Peng, Xiao Chen, et al. "miR-582-3p and miR-582-5p Suppress Prostate Cancer Metastasis to Bone by Repressing TGF-β Signaling". In: *Molecular Therapy - Nucleic Acids* 16.June (2019), pp. 91–104. ISSN: 21622531. DOI: 10.1016/j.omtn.2019.01.004. URL: https://doi.org/10.1016/j.omtn.2019.01.004.

[113] Shu Pin Huang, Eric Levesque, Chantal Guillemette, Chia Cheng Yu, Chao Yuan Huang, Victor C. Lin, et al. "Genetic variants in microRNAs and microRNA target sites predict biochemical recurrence after radical prostatectomy in localized prostate cancer". In: *International Journal of Cancer* 135.11 (2014), pp. 2661–2667. ISSN: 10970215. DOI: 10.1002/ijc.28904.

[114] Emma B. Laursen, Jacob Fredsøe, Linnéa Schmidt, Siri H. Strand, Helle Kristensen, Anne K.I. Rasmussen, et al. "Elevated miR-615-3p Expression Predicts Adverse Clinical Outcome and Promotes Proliferation and Migration of Prostate Cancer Cells". In: *American Journal of Pathology* 189.12 (2019), pp. 2377–2388. ISSN: 15252191. DOI: 10.1016/j.ajpath.2019.08.007. URL: https://doi.org/10.1016/j.ajpath.2019.08.007.

[115] Robert K. Nam, Tania Benatar, Yutaka Amemiya, Christopher J.D. Wallis, Joan Miguel Romero, Melina Tsagaris, et al. "MicroRNA-652 induces NED in LNCaP and EMT in PC3 prostate cancer cells". In: *Oncotarget* 9.27 (2018), pp. 19159–19176. ISSN: 19492553. DOI: 10.18632/oncotarget.24937.

[116] Li Jiao, Zhen Deng, Chuanliang Xu, Yongwei Yu, Yun Li, Chun Yang, et al. "miR-663 induces castration-resistant prostate cancer transformation and predicts clinical recurrence". In: *Journal of Cellular Physiology* 229.7 (2014), pp. 834–844. ISSN: 10974652. DOI: 10.1002/jcp.24510.

[117] Sharanjot Saini, Shahana Majid, Varahram Shahryari, Sumit Arora, Soichiro Yamamura, Inik Chang, et al. "MiRNA-708 control of CD44+ prostate cancer-initiating cells". In: *Cancer Research* 72.14 (2012), pp. 3618–3630. ISSN: 15387445. DOI: 10.1158/0008-5472.CAN-12-0540.

[118] Han Guan, Chunhui Liu, Fang Fang, Yeqing Huang, Tao Tao, Zhixin Ling, et al. "MicroRNA-744 promotes prostate cancer progression through aberrantly activating Wnt/β-catenin signaling". In: *Oncotarget* 8.9 (2017), pp. 14693–14707. ISSN: 19492553. DOI: 10.18632/oncotarget.14711.

[119] Yuemei Yang, Binghan Jia, Xiaoling Zhao, Yao Wang, and Weiliang Ye. "miR-93-5p may be an important oncogene in prostate cancer by bioinformatics analysis". In: *Journal of Cellular Biochemistry* 120.6 (2019), pp. 10463–10483. ISSN: 10974644. DOI: 10.1002/jcb.28332.

[120] Benedikta S. Haflidadóttir, Olivia Larne, Myriam Martin, Margareta Persson, Anders Edsjö, Anders Bjartell, et al. "Upregulation of miR-96 Enhances Cellular Proliferation of Prostate Cancer Cells through FOXO1". In: *PLoS ONE* 8.8 (2013), pp. 1–11. ISSN: 19326203. DOI: 10.1371/journal.pone.0072400.

[121] Helle Kristensen, Anni R. Thomsen, Christa Haldrup, Lars Dyrskjøt, Søren Høyer, Michael Borre, et al. "Novel diagnostic and prognostic classifiers for prostate cancer identified by genome-wide microRNA profiling". In: *Oncotarget* 7.21 (2016). ISSN: 1949-2553. DOI: 10.18632/oncotarget.8953. URL: http://www.oncotarget.com/fulltext/8953.

[122] Jacob Fredsøe, Anne K.I. Rasmussen, Anni R. Thomsen, Peter Mouritzen, Søren Høyer, Michael Borre, et al. "Diagnostic and Prognostic MicroRNA Biomarkers for Prostate Cancer in Cell-free Urine". In: *European Urology Focus* 4.6 (2017), pp. 825–833. ISSN: 24054569. DOI: 10.1016/j.euf.2017.02.018. URL: http://dx.doi.org/10.1016/j.euf.2017.02.018.

[123] L Schmidt, J Fredsøe, H Kristensen, S H Strand, A Rasmussen, S Høyer, et al. "Training and validation of a novel 4-miRNA ratio model (MiCaP) for prediction of postoperative outcome in prostate cancer patients". In: *Annals of Oncology* 29.9 (2018), pp. 2003–2009. DOI: 10.1093/annonc/mdy243.

[124] Olivia Larne, Elena Martens-Uzunova, Zandra Hagman, Anders Edsjö, Giuseppe Lippolis, Mirella S.Vredenbregt Van Den Berg, et al. "MiQ - A novel microRNA based diagnostic and prognostic tool for prostate cancer". In: *International Journal of Cancer* 132.12 (2013), pp. 2867–2875. ISSN: 00207136. DOI: 10.1002/ijc.27973.

[125] Robert K. Nam, Yutaka Amemiya, Tania Benatar, Christopher J.D. Wallis, Jessica Stojcic-Bendavid, Stephanie Bacopulos, et al. "Identification and validation of a five MicroRNA signature predictive of prostate cancer recurrence and metastasis: A cohort study". In: *Journal of Cancer* 6.11 (2015), pp. 1160–1171. ISSN: 18379664. DOI: 10.7150/jca.13397.

[126] Hui Ming Lin, Kate L. Mahon, Calan Spielman, Howard Gurney, Girish Mallesara, Martin R. Stockler, et al. "Phase 2 study of circulating microRNA biomarkers in castration-resistant prostate cancer". In: *British Journal of Cancer* 116.8 (2017), pp. 1002–1011. ISSN: 15321827. DOI: 10.1038/bjc.2017.50.

[127] Sujuan Feng, Xiaosong Qian, Han Li, and Xiaodong Zhang. "Combinations of elevated tissue miRNA-17-92 cluster expression and serum prostate-specific antigen as potential diagnostic biomarkers for prostate cancer". In: *Oncology Letters* 14.6 (2017), pp. 6943–6949. ISSN: 17921082. DOI: 10.3892/ol.2017.7026.

[128] Brittany L. Mihelich, Joseph C. Maranville, Rosalie Nolley, Donna M. Peehl, and Larisa Nonn. "Elevated serum microRNA levels associate with absence of high-grade prostate cancer in a retrospective cohort". In: *PLoS ONE* 10.4 (2015), pp. 1–15. ISSN: 19326203. DOI: 10.1371/journal.pone.0124245.
